# Supplementary material for: Mapping the O‐glycoproteome using site‐specific extraction of O‐linked glycopeptides (EXoO)
Source: Mol Syst Biol. 2018 Nov 20;14(11):e8486. doi: 10.15252/msb.20188486 (PMC6243375; doi:10.15252/msb.20188486)
Supplement: Supplementary file 1 — Appendix [file MSB-14-e8486-s001.pdf]

Table of Contents

Appendix Figure S1: Identification of the O-linked glycan at a new O-linked glycosylation site at Ser-290 in bovine fetuin.

Appendix Figure S2: Supplemental annotated spectra from analysis of human kidney tissue.

Appendix Figure S3: Motif analysis of the O-linked glycosylation sites identified from different samples.

Appendix Figure S4: Biological process and molecular functions of the identified O-linked glycoproteins.

Appendix Figure S5: Localization of O-linked glycosylation sites to protein structural and topological features.

Appendix Table S1: Differentially expressed O-linked glycoproteins in tumor vs normal kidney tissues.

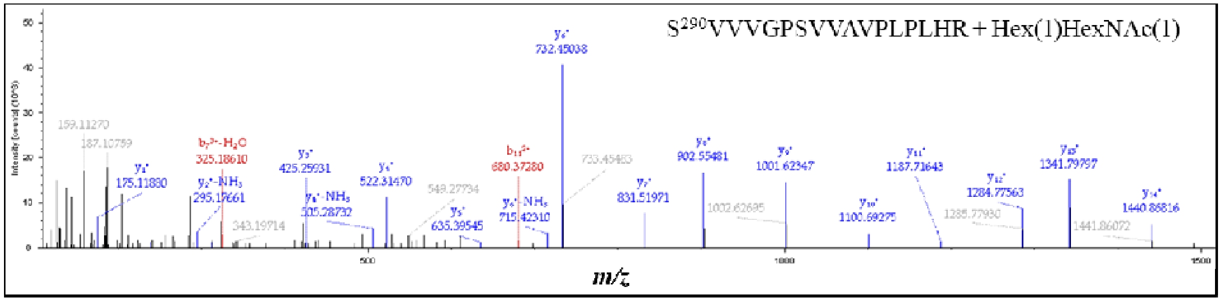

Appendix Figure S1: Identification of the O-linked glycan at a new O-linked glycosylation site at Ser-290 in bovine fetuin.

Appendix Figure S2: Supplemental annotated spectra from analysis of human kidney tissue.

Sequence: TYWPFYPETESS, T1-Hex(1)HexNAc(1) (365.13220 Da)

Charge: +2,   Monoisotopic m/z: 936.39147 Da (+0.56 mmu/+0.6 ppm),   MH+: 1871.77566 Da,   RT: 97.3593 min,

Identified with: Sequest HT (v1.17); XCorr:1.39, Percolator q-Value:7.3e-4, Percolator PEP:3.2e-2, Ions matched by search engine: 0/0

Fragment match tolerance used for search: 0.06 Da

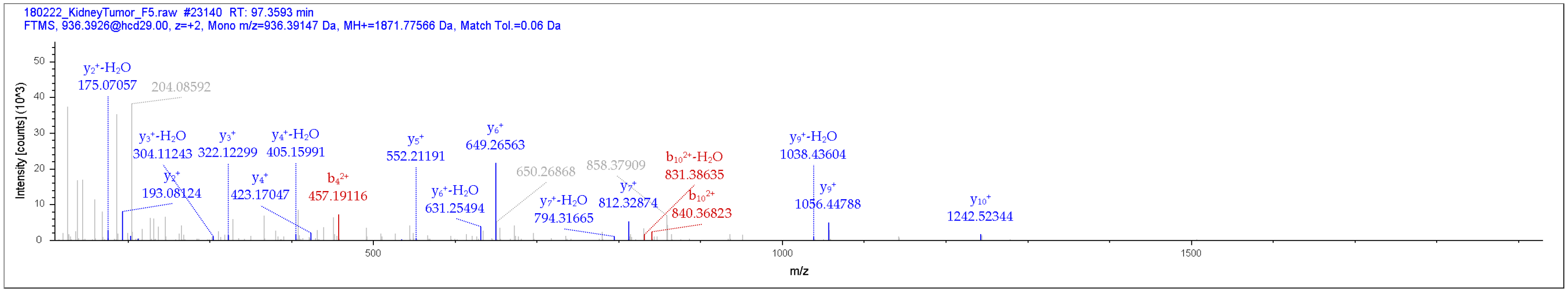

Sequence: TYSVCVMPLGPGR, C5-Carbamidomethyl (57.02146 Da), M7-Oxidation (15.99492 Da), T1-Hex(1)HexNAc(1) (365.13220 Da)

Charge: +3,   Monoisotopic m/z: 606.61496 Da (+0.25 mmu/+0.42 ppm),   MH+: 1817.83034 Da,   RT: 65.5469 min,

Identified with: Sequest HT (v1.17); XCorr:2.73, Percolator q-Value:9.2e-5, Percolator PEP:6.9e-3, Ions matched by search engine: 0/0

Fragment match tolerance used for search: 0.06 Da

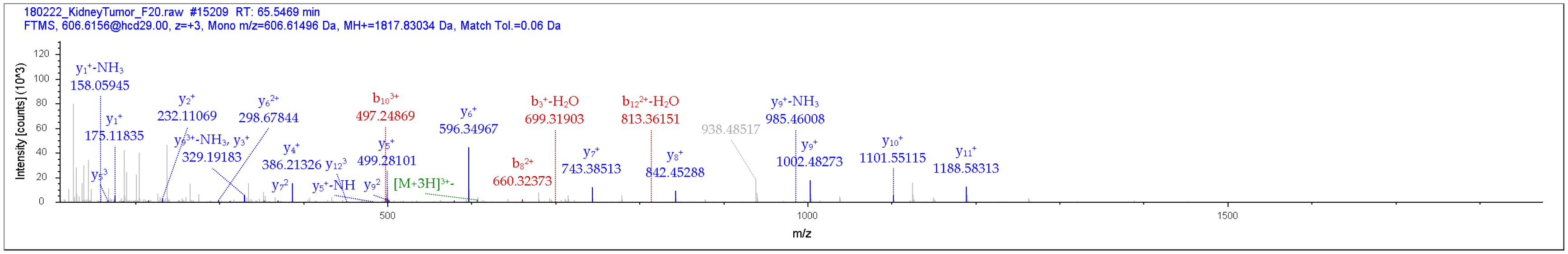

Sequence: TYSAMCPFGCHCHLR, C6-Carbamidomethyl (57.02146 Da), C10-Carbamidomethyl (57.02146 Da), C12-Carbamidomethyl (57.02146 Da), M5-Oxidation (15.99492 Da), T1-Hex(1)HexNAc(1) (365.13220 Da)

Charge: +3,   Monoisotopic m/z: 759.97265 Da (+0.08 mmu/+0.1 ppm),   MH+: 2277.90340 Da,   RT: 48.7438 min,

Identified with: Sequest HT (v1.17); XCorr:4.08, Percolator q-Value:0.0e0, Percolator PEP:1.2e-5, Ions matched by search engine: 0/0

Fragment match tolerance used for search: 0.06 Da

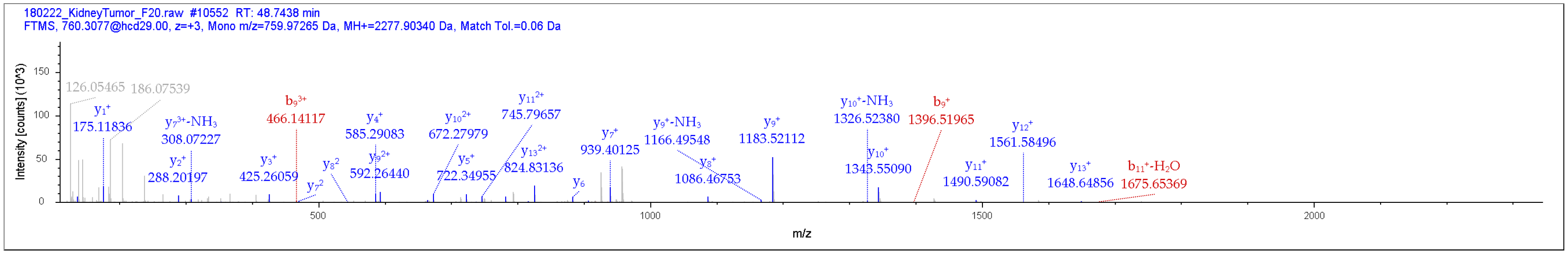

Sequence: TYQVPDALEWEQQNQGLVR, T1-Hex(1)HexNAc(1) (365.13220 Da)

Charge: +2,   Monoisotopic m/z: 1320.12895 Da (+1.74 mmu/+1.32 ppm),   MH+: 2639.25062 Da,   RT: 94.0356 min,

Identified with: Sequest HT (v1.17); XCorr:3.62, Percolator q-Value:0.0e0, Percolator PEP:8.1e-6, Ions matched by search engine: 0/0

Fragment match tolerance used for search: 0.06 Da

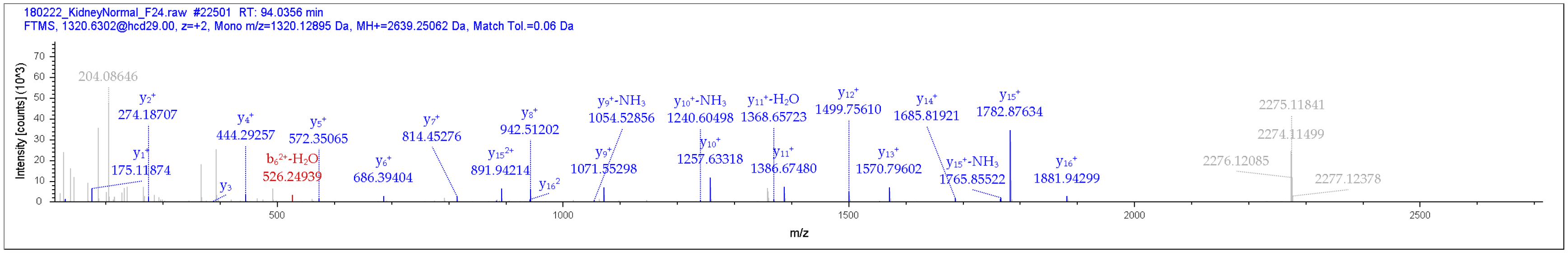

Sequence: TWPDMELPLPR, M5-Oxidation (15.99492 Da), T1-Hex(1)HexNAc(1) (365.13220 Da)

Charge: +3,   Monoisotopic m/z: 579.27558 Da (+0.89 mmu/+1.54 ppm),   MH+: 1735.81217 Da,   RT: 92.9942 min,

Identified with: Sequest HT (v1.17); XCorr:2.24, Percolator q-Value:5.0e-5, Percolator PEP:6.6e-3, Ions matched by search engine: 0/0

Fragment match tolerance used for search: 0.06 Da

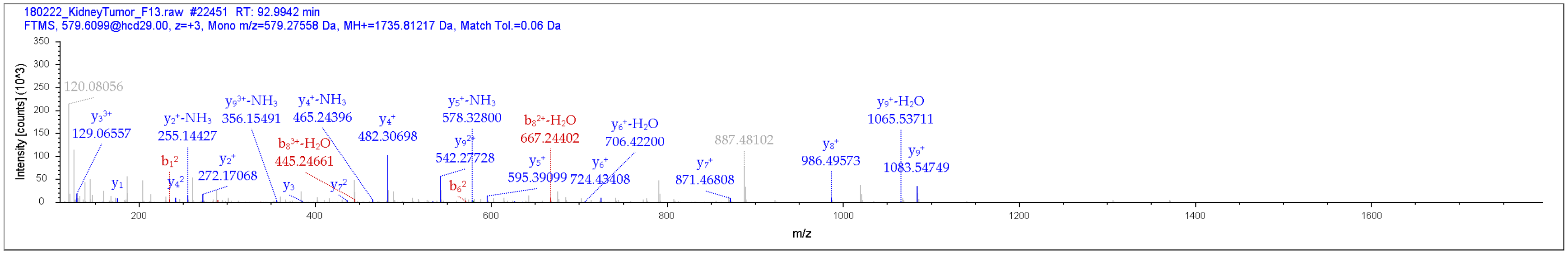

Sequence: TWPDMELPLPR, M5-Oxidation (15.99492 Da), T1-Hex(1)HexNAc(1) (365.13220 Da)

Charge: +3,   Monoisotopic m/z: 579.27591 Da (+1.22 mmu/+2.11 ppm),   MH+: 1735.81316 Da,   RT: 82.6916 min,

Identified with: Sequest HT (v1.17); XCorr:2.32, Percolator q-Value:0.0e0, Percolator PEP:1.0e-3, Ions matched by search engine: 0/0

Fragment match tolerance used for search: 0.06 Da

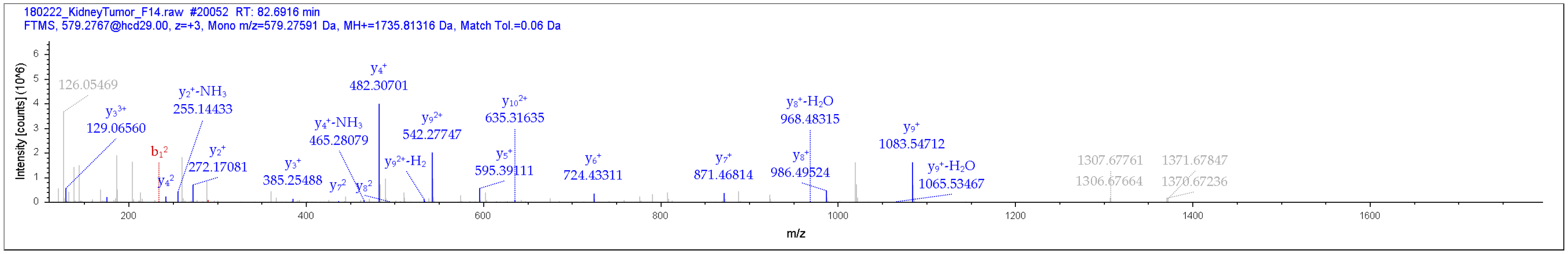

Sequence: TWPDMELPLPR, M5-Oxidation (15.99492 Da), T1-Hex(1)HexNAc(1) (365.13220 Da)

Charge: +2,   Monoisotopic m/z: 868.40914 Da (+0.76 mmu/+0.87 ppm),   MH+: 1735.81101 Da,   RT: 83.6742 min,

Identified with: Sequest HT (v1.17); XCorr:1.30, Percolator q-Value:5.4e-4, Percolator PEP:3.0e-2, Ions matched by search engine: 0/0

Fragment match tolerance used for search: 0.06 Da

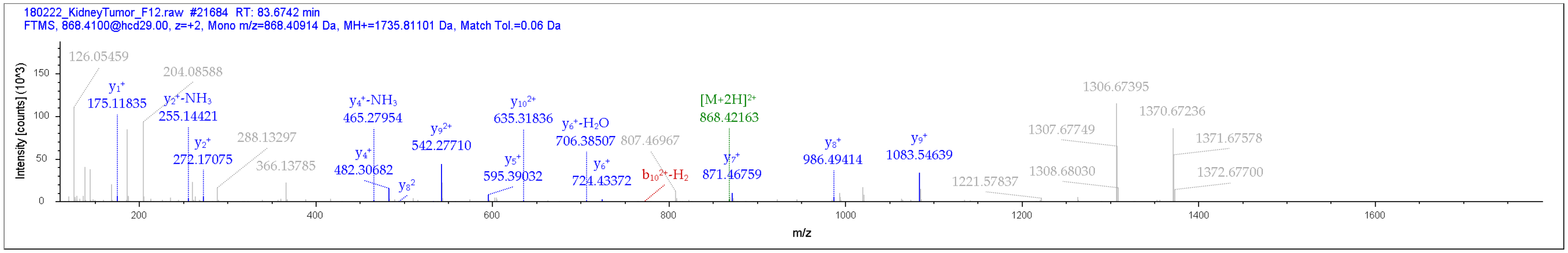

Sequence: TWPDMELPLPR, M5-Oxidation (15.99492 Da), T1-Hex(1)HexNAc(1) (365.13220 Da)

Charge: +3,   Monoisotopic m/z: 579.27395 Da (-0.73 mmu/-1.26 ppm),   MH+: 1735.80730 Da,   RT: 95.2596 min,

Identified with: Sequest HT (v1.17); XCorr:2.26, Percolator q-Value:0.0e0, Percolator PEP:1.3e-3, Ions matched by search engine: 0/0

Fragment match tolerance used for search: 0.06 Da

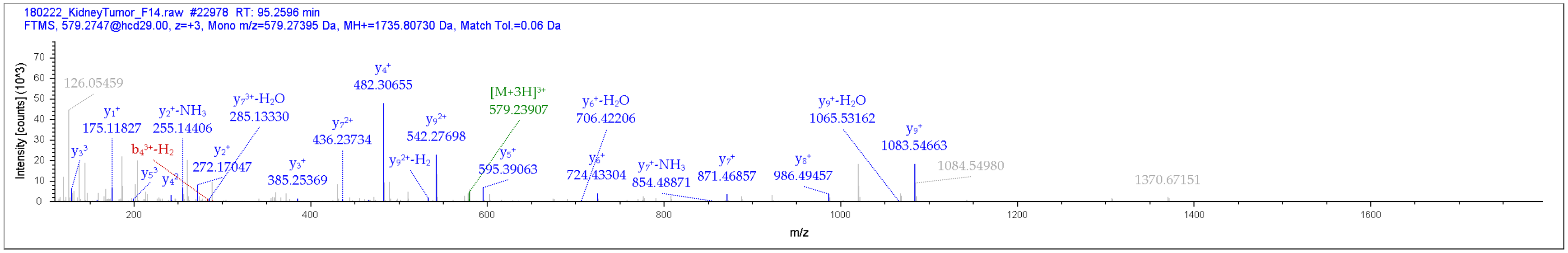

Sequence: TWPDMELPLPR, M5-Oxidation (15.99492 Da), T1-Hex(1)HexNAc(1) (365.13220 Da)

Charge: +2,   Monoisotopic m/z: 868.40736 Da (-1.03 mmu/-1.18 ppm),   MH+: 1735.80744 Da,   RT: 82.6101 min,

Identified with: Sequest HT (v1.17); XCorr:1.38, Percolator q-Value:0.0e0, Percolator PEP:2.0e-3, Ions matched by search engine: 0/0

Fragment match tolerance used for search: 0.06 Da

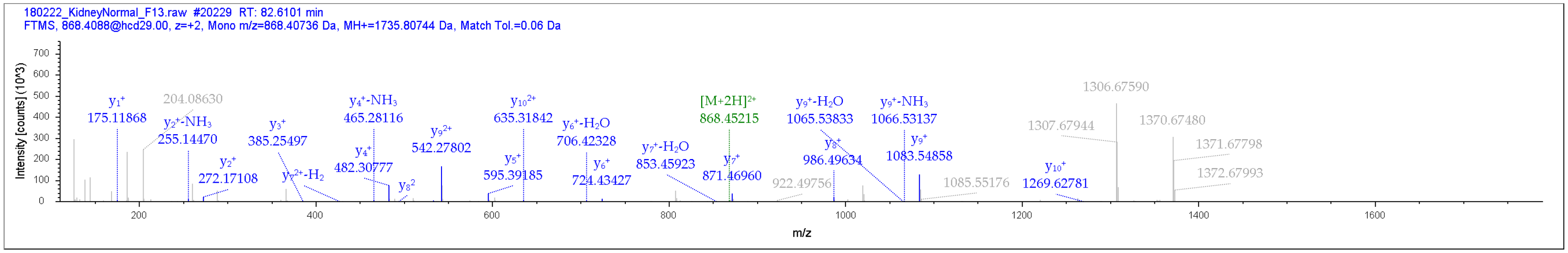

Sequence: TWPDMELPLPR, M5-Oxidation (15.99492 Da), T1-Hex(1)HexNAc(1) (365.13220 Da)

Charge: +2,   Monoisotopic m/z: 868.40775 Da (-0.64 mmu/-0.73 ppm),   MH+: 1735.80822 Da,   RT: 87.6011 min,

Identified with: Sequest HT (v1.17); XCorr:1.48, Percolator q-Value:0.0e0, Percolator PEP:5.6e-4, Ions matched by search engine: 0/0

Fragment match tolerance used for search: 0.06 Da

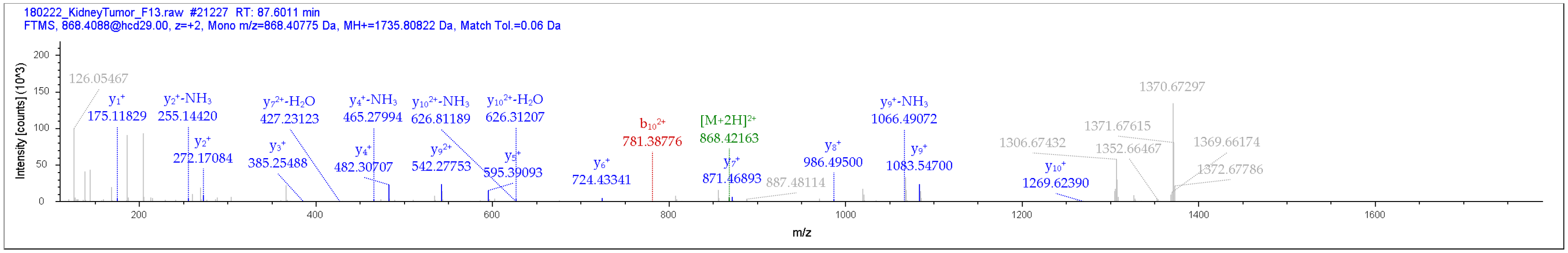

Sequence: TWPDMELPLPR, M5-Oxidation (15.99492 Da), T1-Hex(1)HexNAc(1) (365.13220 Da)

Charge: +3, Monoisotopic m/z: 579.27492 Da (+0.23 mmu/+0.4 ppm), MH+: 1735.81020 Da, RT: 85.1609 min,

Identified with: Sequest HT (v1.17); XCorr:2.05, Percolator q-Value:5.0e-5, Percolator PEP:2.1e-3, Ions matched by search engine: 0/0

Fragment match tolerance used for search: 0.06 Da

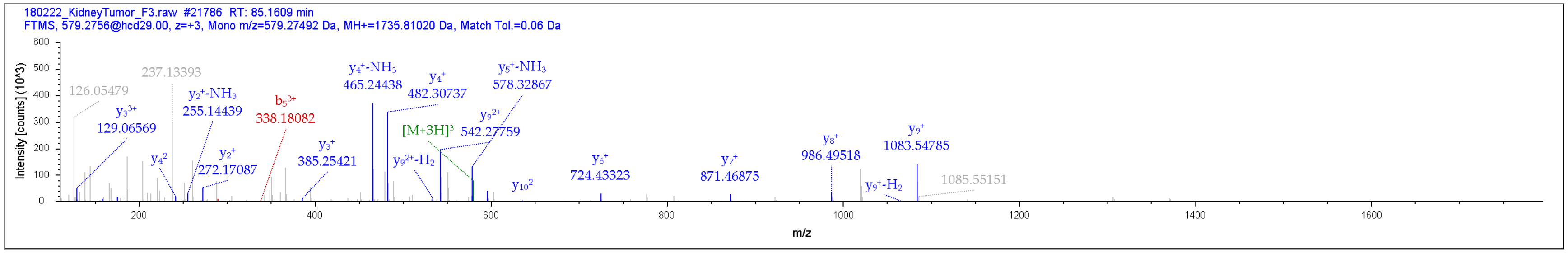

Sequence: TWNPGSSER, T1-Hex(1)HexNAc(1) (365.13220 Da)

Charge: +2, Monoisotopic m/z: 699.80421 Da (-0.4 mmu/-0.57 ppm), MH+: 1398.60115 Da, RT: 34.9253 min,

Identified with: Sequest HT (v1.17); XCorr:1.44, Percolator q-Value:2.1e-4, Percolator PEP:1.4e-2, Ions matched by search engine: 0/0

Fragment match tolerance used for search: 0.06 Da

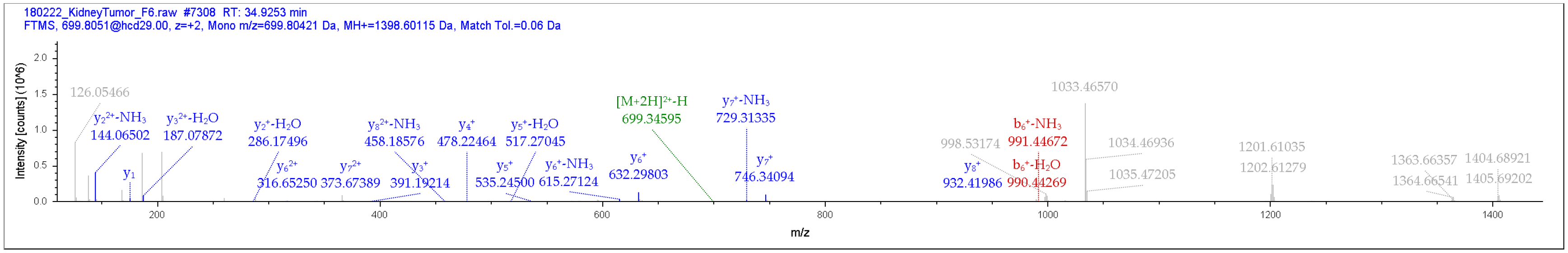

Sequence: TWLNPDPSQK, K10-Guanidinyl (42.02180 Da), T1-Hex(1)HexNAc(1) (365.13220 Da)

Charge: +2, Monoisotopic m/z: 796.87429 Da (-1.28 mmu/-1.6 ppm), MH+: 1592.74131 Da, RT: 57.4758 min,

Identified with: Sequest HT (v1.17); XCorr:0.95, Percolator q-Value:3.6e-3, Percolator PEP:1.2e-1, Ions matched by search engine: 0/0

Fragment match tolerance used for search: 0.06 Da

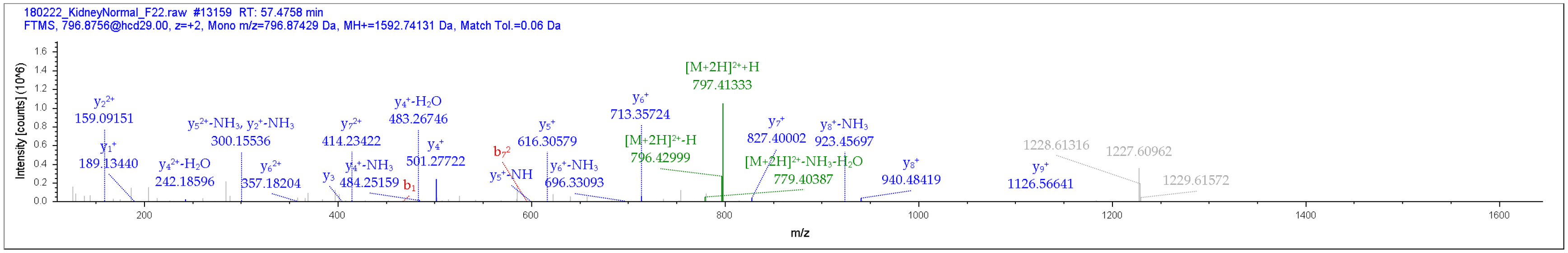

Sequence: TWKPGSSGPGSTGSWN, K3-Guanidinyl (42.02180 Da), T1-Hex(1)HexNAc(1) (365.13220 Da)

Charge: +2, Monoisotopic m/z: 1006.94605 Da (+0.81 mmu/+0.81 ppm), MH+: 2012.88483 Da, RT: 56.0408 min,

Identified with: Sequest HT (v1.17); XCorr:2.34, Percolator q-Value:0.0e0, Percolator PEP:3.1e-3, Ions matched by search engine: 0/0

Fragment match tolerance used for search: 0.06 Da

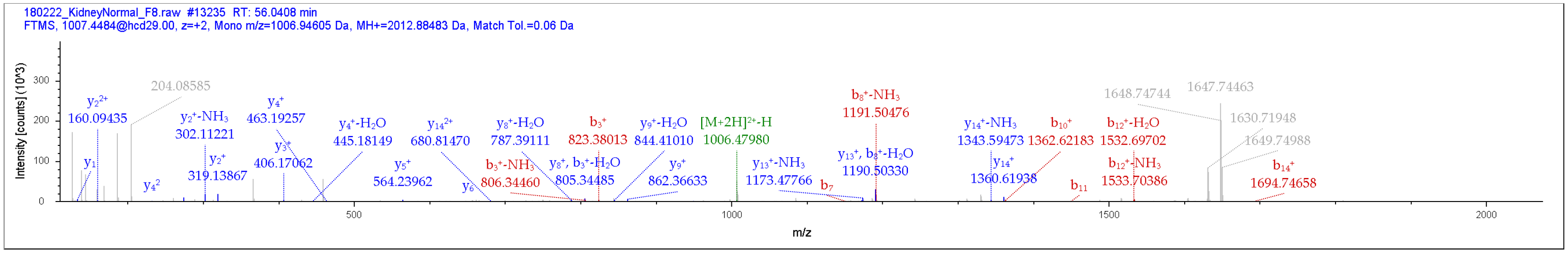

Sequence: TWEEQQTSVLHLVGDNFR, T1-Hex(1)HexNAc(1) (365.13220 Da)

Charge: +3, Monoisotopic m/z: 842.06713 Da (+1.01 mmu/+1.2 ppm), MH+: 2524.18684 Da, RT: 89.6926 min,

Identified with: Sequest HT (v1.17); XCorr:4.82, Percolator q-Value:0.0e0, Percolator PEP:2.0e-6, Ions matched by search engine: 0/0

Fragment match tolerance used for search: 0.06 Da

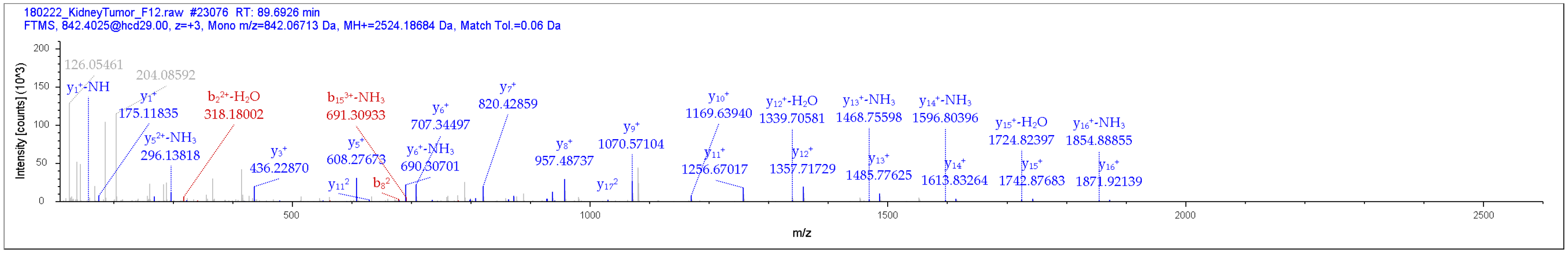

Sequence: TWDGSVR, T1-Hex(1)HexNAc(1) (365.13220 Da)

Charge: +2, Monoisotopic m/z: 593.26722 Da (+0.09 mmu/+0.15 ppm), MH+: 1185.52717 Da, RT: 32.2525 min,

Identified with: Sequest HT (v1.17); XCorr:1.02, Percolator q-Value:4.0e-4, Percolator PEP:3.7e-2, Ions matched by search engine: 0/0

Fragment match tolerance used for search: 0.06 Da

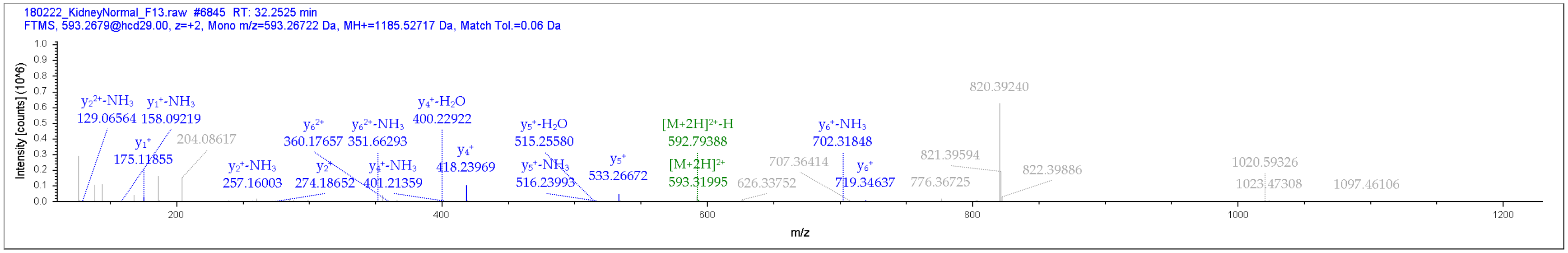

Sequence: TVYPPEEETGER, T1-Hex(1)HexNAc(1) (365.13220 Da)

Charge: +2,   Monoisotopic m/z: 886.39178 Da (+0.34 mmu/+0.38 ppm),   MH+: 1771.77629 Da,   RT: 38.9056 min,

Identified with: Sequest HT (v1.17); XCorr:1.49, Percolator q-Value:9.2e-5, Percolator PEP:8.4e-3, Ions matched by search engine: 0/0

Fragment match tolerance used for search: 0.06 Da

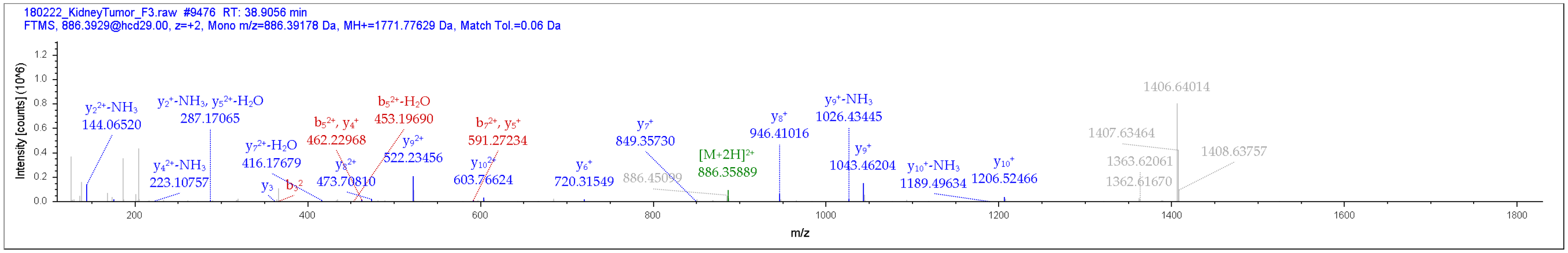

Sequence: TVYPPEEETGER, T1-Hex(1)HexNAc(1) (365.13220 Da)

Charge: +2,   Monoisotopic m/z: 886.39108 Da (-0.37 mmu/-0.41 ppm),   MH+: 1771.77488 Da,   RT: 37.2371 min,

Identified with: Sequest HT (v1.17); XCorr:1.30, Percolator q-Value:0.0e0, Percolator PEP:4.7e-4, Ions matched by search engine: 0/0

Fragment match tolerance used for search: 0.06 Da

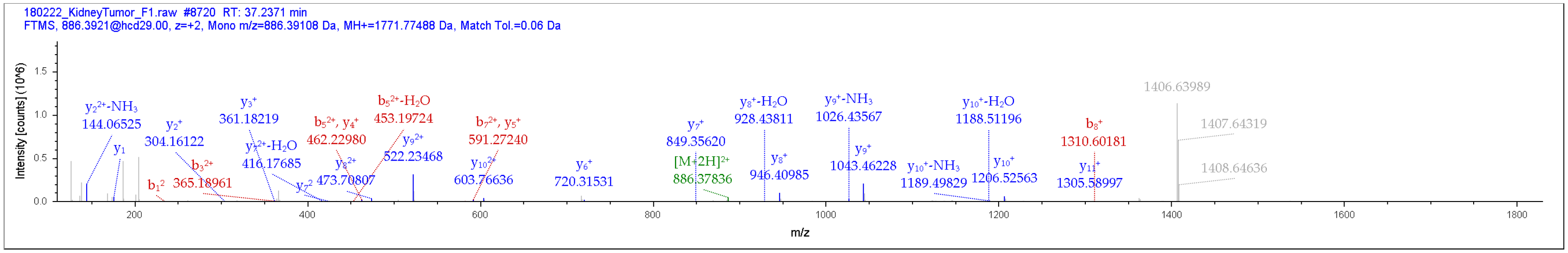

Sequence: TVVGIPDGTAVVGR, T1-HexNAc (203.07937 Da)

Charge: +3, Monoisotopic m/z: 515.28214 Da (-0.25 mmu/-0.49 ppm), MH+: 1543.83186 Da, RT: 69.5726 min,

Identified with: Sequest HT (v1.17); XCorr:3.66, Percolator q-Value:0.0e0, Percolator PEP:6.4e-3, Ions matched by search engine: 0/0

Fragment match tolerance used for search: 0.06 Da

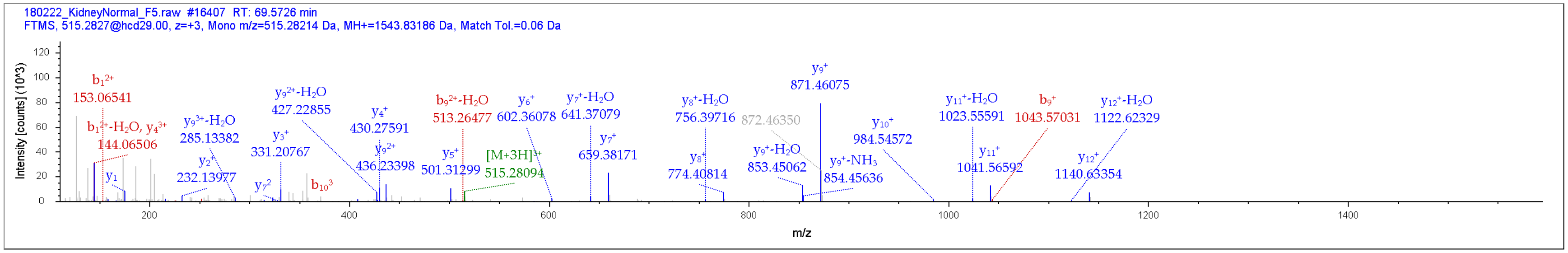

Sequence: TVTVPEGDTAR, T1-Hex(1)HexNAc(1) (365.13220 Da)

Charge: +2, Monoisotopic m/z: 755.85941 Da (-0.17 mmu/-0.23 ppm), MH+: 1510.71154 Da, RT: 36.2377 min,

Identified with: Sequest HT (v1.17); XCorr:1.57, Percolator q-Value:0.0e0, Percolator PEP:6.9e-3, Ions matched by search engine: 0/0

Fragment match tolerance used for search: 0.06 Da

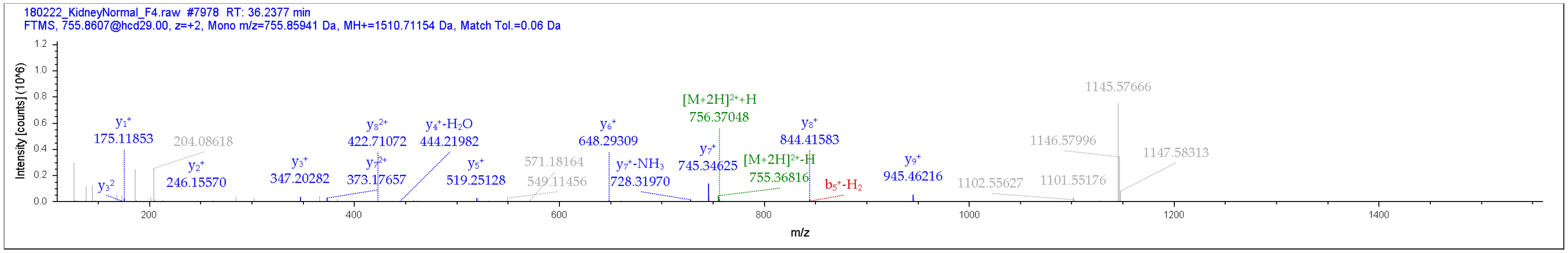

Sequence: TVTTVGFMPLSTQVR, M8-Oxidation (15.99492 Da), T1-Hex(1)HexNAc(1) (365.13220 Da)

Charge: +3, Monoisotopic m/z: 673.33821 Da (+0.08 mmu/+0.13 ppm), MH+: 2018.00007 Da, RT: 75.2133 min,

Identified with: Sequest HT (v1.17); XCorr:3.35, Percolator q-Value:0.0e0, Percolator PEP:7.0e-4, Ions matched by search engine: 0/0

Fragment match tolerance used for search: 0.06 Da

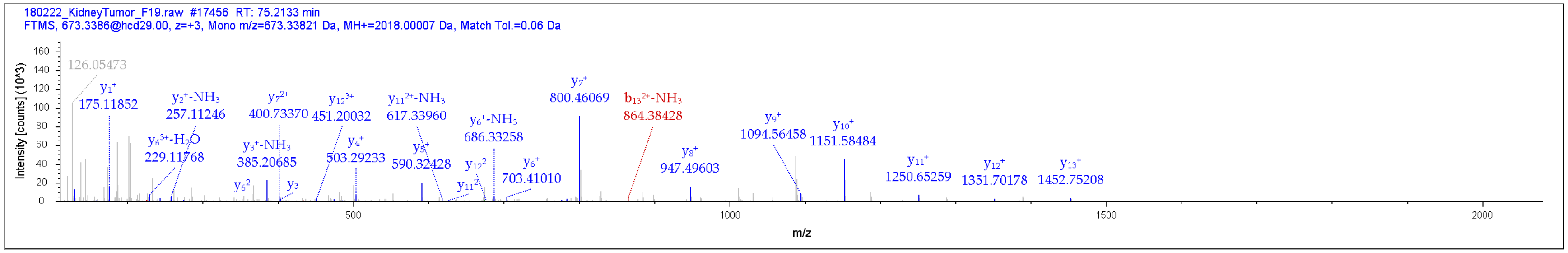

Sequence: TVSTNFPQTIEPAK, K14-Guanidinyl (42.02180 Da), T1-Hex(1)HexNAc(1) (365.13220 Da), S3-Hex(1)HexNAc(1) (365.13220 Da), T4-Hex(1)HexNAc(1) (365.13220 Da)

Charge: +3, Monoisotopic m/z: 890.74507 Da (+2.26 mmu/+2.53 ppm), MH+: 2670.22065 Da, RT: 57.9131 min,

Identified with: Sequest HT (v1.17); XCorr:1.94, Percolator q-Value:2.8e-4, Percolator PEP:2.0e-2, Ions matched by search engine: 0/0

Fragment match tolerance used for search: 0.06 Da

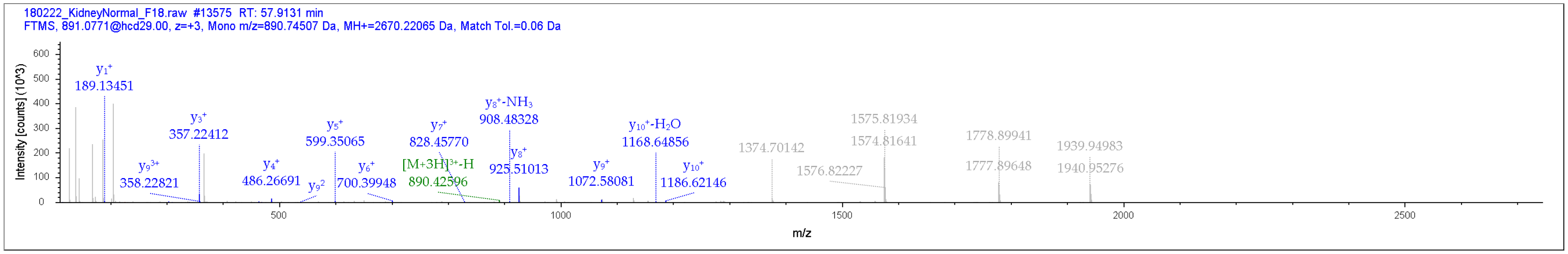

Sequence: TVSSFSLNVEYAIQAEK, K17-Guanidinyl (42.02180 Da), S3-Hex(1)HexNAc(1) (365.13220 Da), S4-Hex(1)HexNAc(1) (365.13220 Da)

Charge: +3,   Monoisotopic m/z: 886.75320 Da (+1.56 mmu/+1.76 ppm),   MH+: 2658.24506 Da,   RT: 94.0757 min,

Identified with: Sequest HT (v1.17); XCorr:3.23, Percolator q-Value:0.0e0, Percolator PEP:4.1e-4, Ions matched by search engine: 0/0

Fragment match tolerance used for search: 0.06 Da

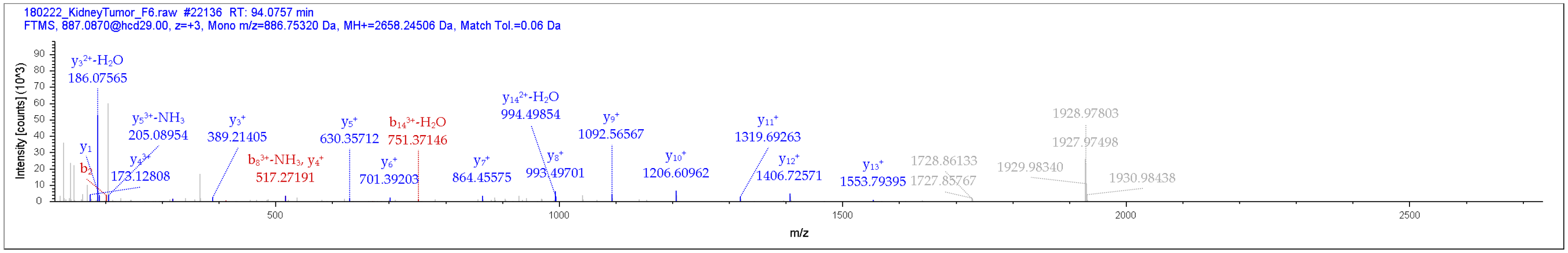

Sequence: TVSHPIAK, K8-Guanidinyl (42.02180 Da), T1-Hex(1)HexNAc(1) (365.13220 Da)

Charge: +3,   Monoisotopic m/z: 420.55359 Da (-0.52 mmu/-1.24 ppm),   MH+: 1259.64621 Da,   RT: 22.7310 min,

Identified with: Sequest HT (v1.17); XCorr:1.91, Percolator q-Value:1.4e-4, Percolator PEP:1.2e-2, Ions matched by search engine: 0/0

Fragment match tolerance used for search: 0.06 Da

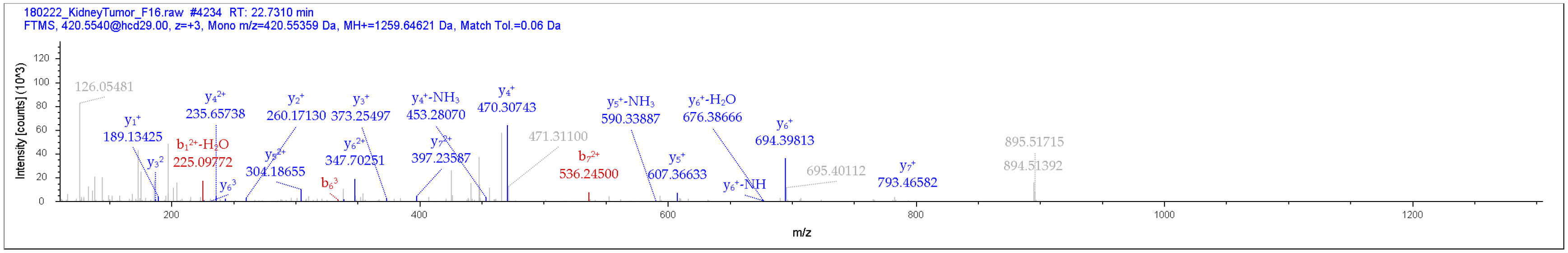

Sequence: TVQSGGSHTALGDR, T1-Hex(1)HexNAc(1) (365.13220 Da)

Charge: +3, Monoisotopic m/z: 584.27483 Da (+0.32 mmu/+0.55 ppm), MH+: 1750.80993 Da, RT: 22.5171 min,

Identified with: Sequest HT (v1.17); XCorr:3.09, Percolator q-Value:0.0e0, Percolator PEP:4.9e-4, Ions matched by search engine: 0/0

Fragment match tolerance used for search: 0.06 Da

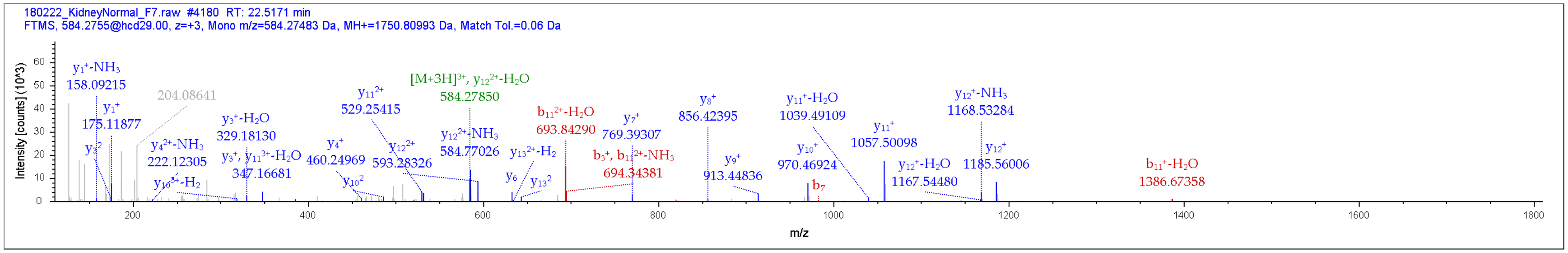

Sequence: TVPPSGLSVPFSTWDQDHDLR, T1-Hex(1)HexNAc(1) (365.13220 Da)

Charge: +3, Monoisotopic m/z: 907.09584 Da (-0.12 mmu/-0.13 ppm), MH+: 2719.27298 Da, RT: 89.5883 min,

Identified with: Sequest HT (v1.17); XCorr:4.73, Percolator q-Value:0.0e0, Percolator PEP:5.0e-9, Ions matched by search engine: 0/0

Fragment match tolerance used for search: 0.06 Da

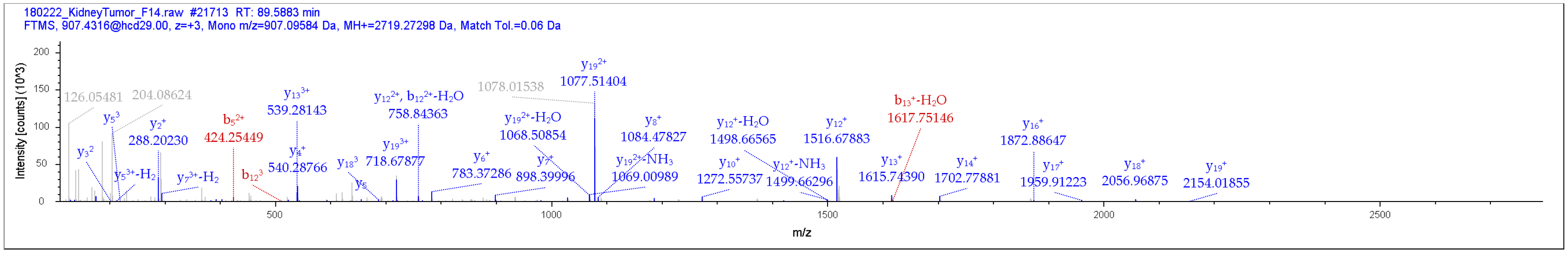

Sequence: TVPPLQPFVGR, T1-Hex(1)HexNAc(1) (365.13220 Da)

Charge: +3, Monoisotopic m/z: 525.94700 Da (-0.01 mmu/-0.02 ppm), MH+: 1575.82643 Da, RT: 74.2783 min,

Identified with: Sequest HT (v1.17); XCorr:2.79, Percolator q-Value:0.0e0, Percolator PEP:5.4e-3, Ions matched by search engine: 0/0

Fragment match tolerance used for search: 0.06 Da

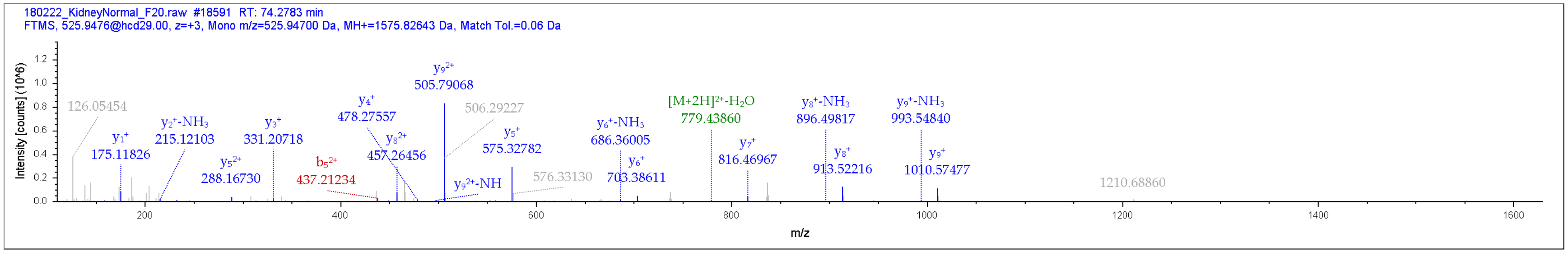

Sequence: TVPFHEEFESGTAK, K14-Guanidinyl (42.02180 Da), T1-Hex(1)HexNAc(1) (365.13220 Da)

Charge: +3, Monoisotopic m/z: 662.63883 Da (+1.5 mmu/+2.26 ppm), MH+: 1985.90194 Da, RT: 60.6562 min,

Identified with: Sequest HT (v1.17); XCorr:3.32, Percolator q-Value:5.0e-5, Percolator PEP:3.5e-3, Ions matched by search engine: 0/0

Fragment match tolerance used for search: 0.06 Da

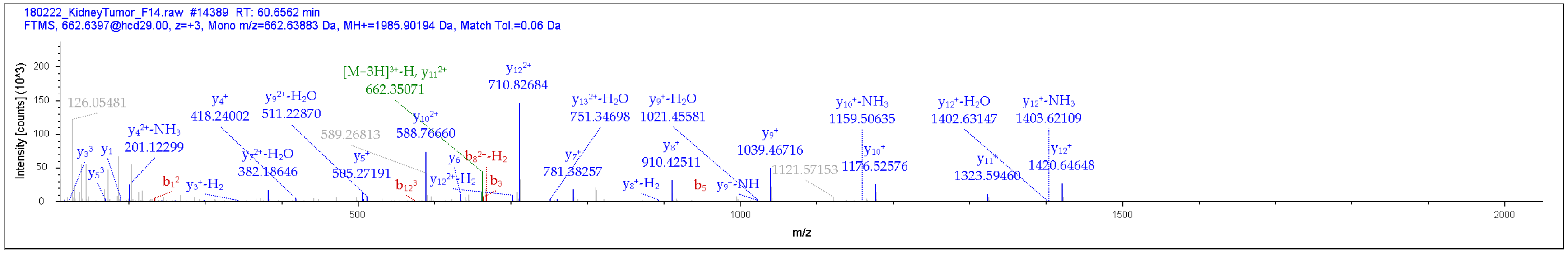

Sequence: TVPEGQFDHFLVQYK, T1-HexNAc (203.07937 Da), K15-Guanidinyl (42.02180 Da)

Charge: +3, Monoisotopic m/z: 685.00578 Da (+0.08 mmu/+0.12 ppm), MH+: 2053.00278 Da, RT: 86.6504 min,

Identified with: Sequest HT (v1.17); XCorr:4.11, Percolator q-Value:0.0e0, Percolator PEP:8.6e-5, Ions matched by search engine: 0/0

Fragment match tolerance used for search: 0.06 Da

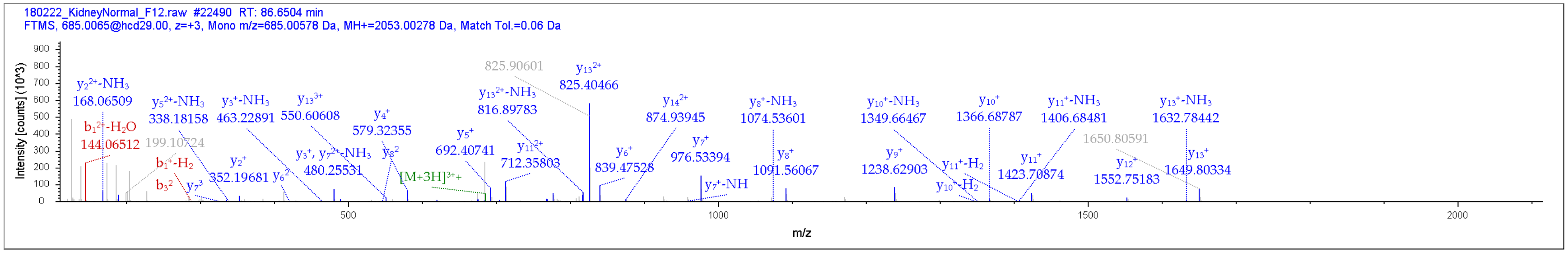

Sequence: TVMPILSSGMECLTR, C12-Carbamidomethyl (57.02146 Da), T1-Hex(1)HexNAc(1) (365.13220 Da)

Charge: +3, Monoisotopic m/z: 687.32547 Da (+0.75 mmu/+1.09 ppm), MH+: 2059.96185 Da, RT: 94.4202 min,

Identified with: Sequest HT (v1.17); XCorr:4.05, Percolator q-Value:0.0e0, Percolator PEP:8.2e-5, Ions matched by search engine: 0/0

Fragment match tolerance used for search: 0.06 Da

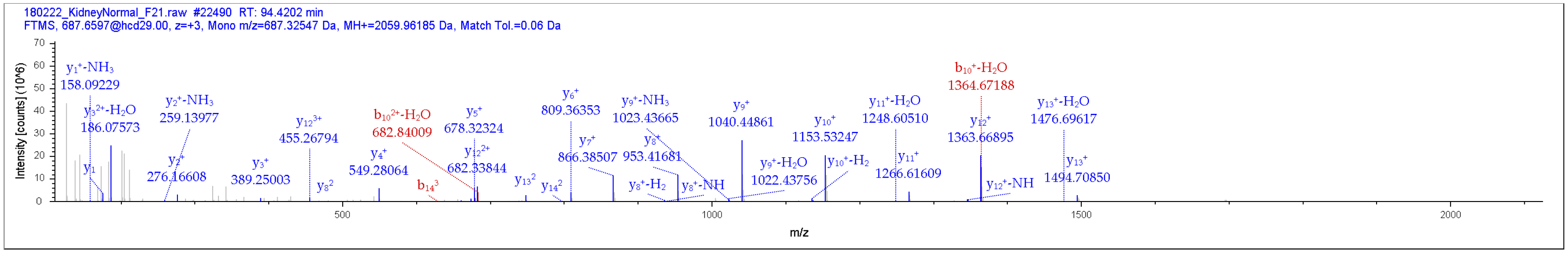

Sequence: TVAPPMVRP, M6-Oxidation (15.99492 Da), T1-HexNAc (203.07937 Da)

Charge: +3,   Monoisotopic m/z: 396.20948 Da (+0.08 mmu/+0.21 ppm),   MH+: 1186.61389 Da,   RT: 42.9222 min,

Identified with: Sequest HT (v1.17); XCorr:2.20, Percolator q-Value:1.7e-4, Percolator PEP:1.1e-2, Ions matched by search engine: 0/0

Fragment match tolerance used for search: 0.06 Da

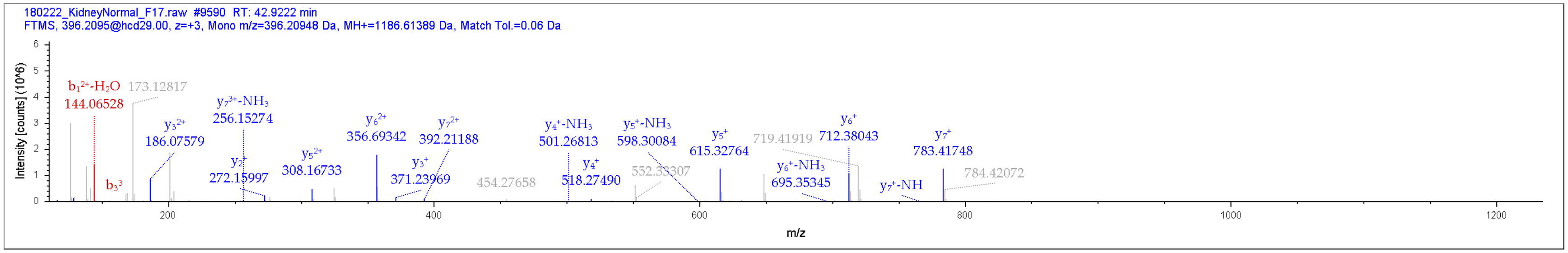

Sequence: TVAPPMVRP, M6-Oxidation (15.99492 Da), T1-Hex(1)HexNAc(1) (365.13220 Da)

Charge: +2,   Monoisotopic m/z: 674.83835 Da (+1.48 mmu/+2.2 ppm),   MH+: 1348.66942 Da,   RT: 43.6263 min,

Identified with: Sequest HT (v1.17); XCorr:1.53, Percolator q-Value:2.9e-4, Percolator PEP:2.0e-2, Ions matched by search engine: 0/0

Fragment match tolerance used for search: 0.06 Da

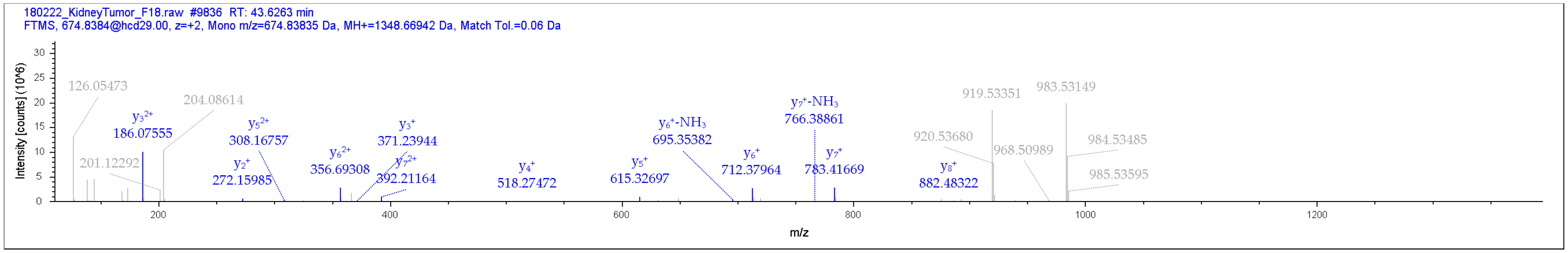

Sequence: TVAPPMVRP, M6-Oxidation (15.99492 Da), T1-HexNAc (203.07937 Da)

Charge: +3, Monoisotopic m/z: 396.20887 Da (-0.53 mmu/-1.34 ppm), MH+: 1186.61204 Da, RT: 48.4719 min,

Identified with: Sequest HT (v1.17); XCorr:2.42, Percolator q-Value:4.9e-4, Percolator PEP:4.0e-2, Ions matched by search engine: 0/0

Fragment match tolerance used for search: 0.06 Da

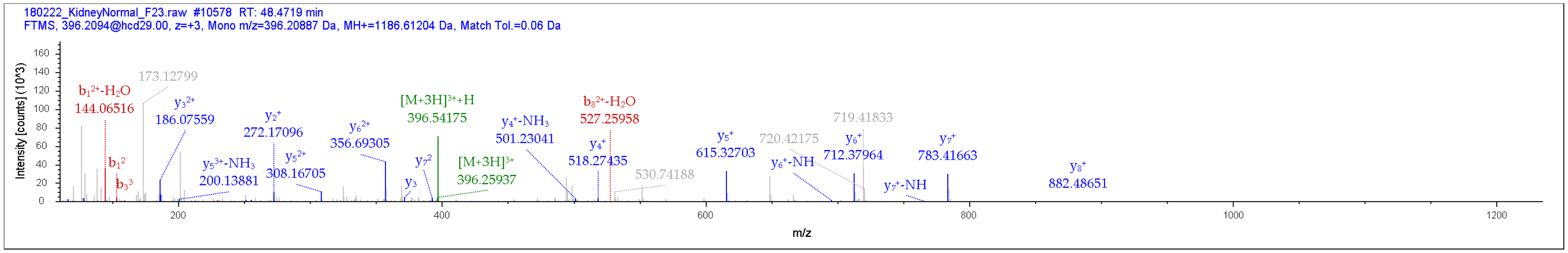

Sequence: TVAPPIHQGPAVPTAVIPLPPGTHLLFAQTGK, T14-HexNAc (203.07937 Da), K32-Guanidinyl (42.02180 Da), T1-Hex(1)HexNAc(1) (365.13220 Da)

Charge: +5, Monoisotopic m/z: 768.01596 Da (+0.53 mmu/+0.69 ppm), MH+: 3836.05069 Da, RT: 102.3925 min,

Identified with: Sequest HT (v1.17); XCorr:3.26, Percolator q-Value:6.9e-3, Percolator PEP:1.4e-1, Ions matched by search engine: 0/0

Fragment match tolerance used for search: 0.06 Da

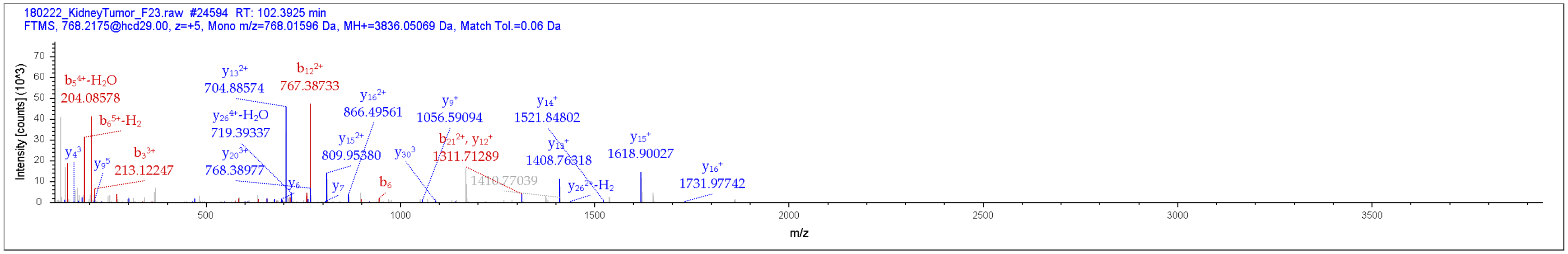

Sequence: TVAPDNTAIPSLR, T1-Hex(1)HexNAc(1) (365.13220 Da)

Charge: +3,   Monoisotopic m/z: 573.95953 Da (-0.22 mmu/-0.39 ppm),   MH+: 1719.86403 Da,   RT: 60.1894 min,

Identified with: Sequest HT (v1.17); XCorr:3.76, Percolator q-Value:1.4e-4, Percolator PEP:1.2e-2, Ions matched by search engine: 0/0

Fragment match tolerance used for search: 0.06 Da

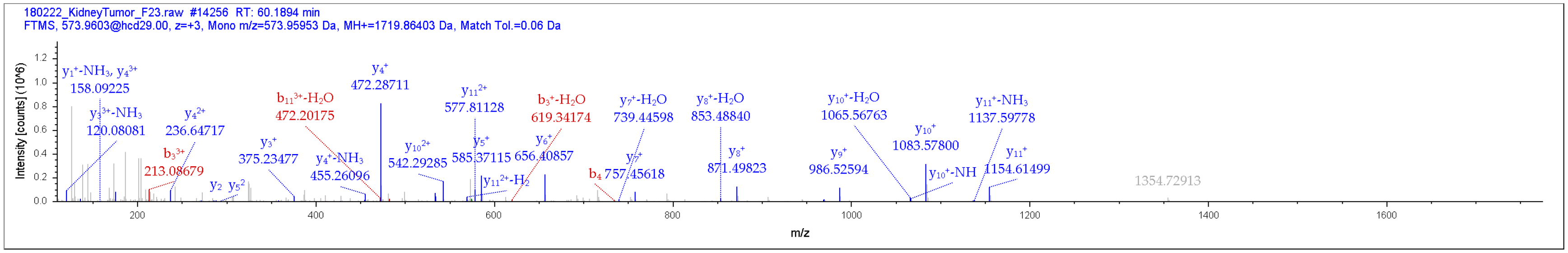

Sequence: TVAPAEDETLQNEADNQENVLSQLLGDYDK, K30-Guanidinyl (42.02180 Da), T9-Hex(1)HexNAc(1) (365.13220 Da)

Charge: +3,   Monoisotopic m/z: 1242.90406 Da (-0.4 mmu/-0.32 ppm),   MH+: 3726.69761 Da,   RT: 115.9834 min,

Identified with: Sequest HT (v1.17); XCorr:1.84, Percolator q-Value:5.0e-5, Percolator PEP:2.5e-3, Ions matched by search engine: 0/0

Fragment match tolerance used for search: 0.06 Da

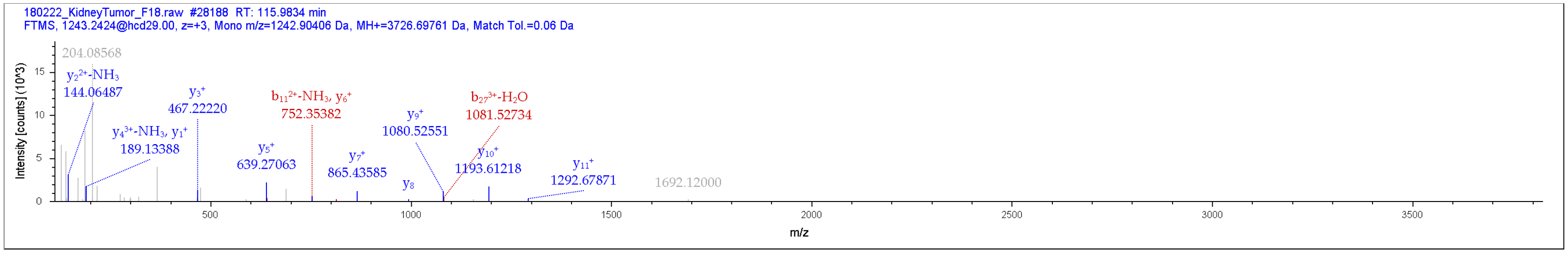

Sequence: TVAHESNWAK, T1-HexNAc (203.07937 Da), K10-Guanidinyl (42.02180 Da)

Charge: +3, Monoisotopic m/z: 463.22434 Da (-0.54 mmu/-1.16 ppm), MH+: 1387.65846 Da, RT: 29.6670 min,

Identified with: Sequest HT (v1.17); XCorr:3.20, Percolator q-Value:9.2e-5, Percolator PEP:9.0e-3, Ions matched by search engine: 0/0

Fragment match tolerance used for search: 0.06 Da

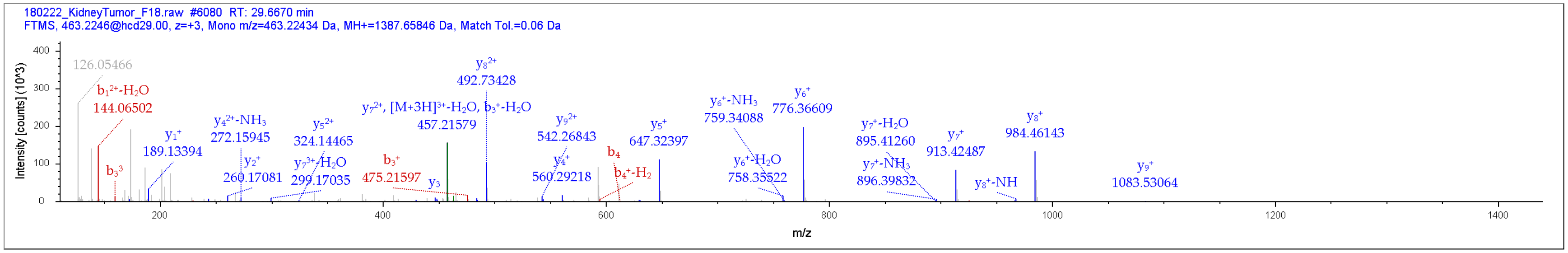

Sequence: TVAHESNWAK, T1-HexNAc (203.07937 Da), K10-Guanidinyl (42.02180 Da)

Charge: +3, Monoisotopic m/z: 463.22438 Da (-0.49 mmu/-1.06 ppm), MH+: 1387.65860 Da, RT: 30.4268 min,

Identified with: Sequest HT (v1.17); XCorr:3.35, Percolator q-Value:2.2e-4, Percolator PEP:1.2e-2, Ions matched by search engine: 0/0

Fragment match tolerance used for search: 0.06 Da

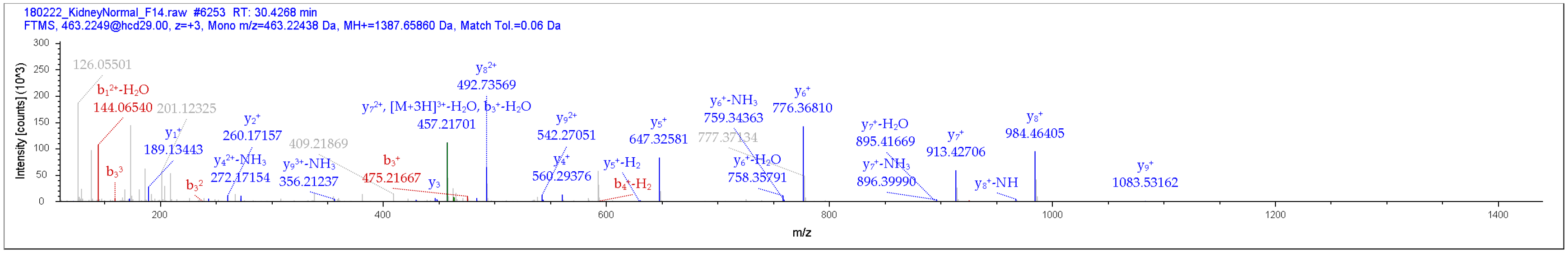

Sequence: TTVPPSGLSVPFSTWDQDHDLR, T2-Hex(1)HexNAc(1) (365.13220 Da)

Charge: +3, Monoisotopic m/z: 940.77955 Da (+1.03 mmu/+1.09 ppm), MH+: 2820.32410 Da, RT: 91.2756 min,

Identified with: Sequest HT (v1.17); XCorr:4.88, Percolator q-Value:0.0e0, Percolator PEP:8.2e-9, Ions matched by search engine: 0/0

Fragment match tolerance used for search: 0.06 Da

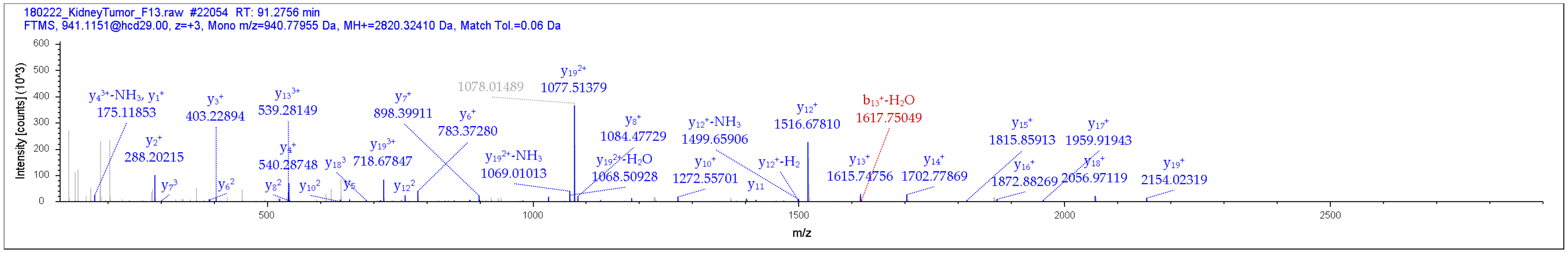

Sequence: TTVPPSGLSVPFSTWDQDHDLR, T2-Hex(1)HexNAc(1) (365.13220 Da)

Charge: +3, Monoisotopic m/z: 940.77949 Da (+0.97 mmu/+1.03 ppm), MH+: 2820.32392 Da, RT: 91.0300 min,

Identified with: Sequest HT (v1.17); XCorr:5.35, Percolator q-Value:0.0e0, Percolator PEP:3.4e-8, Ions matched by search engine: 0/0

Fragment match tolerance used for search: 0.06 Da

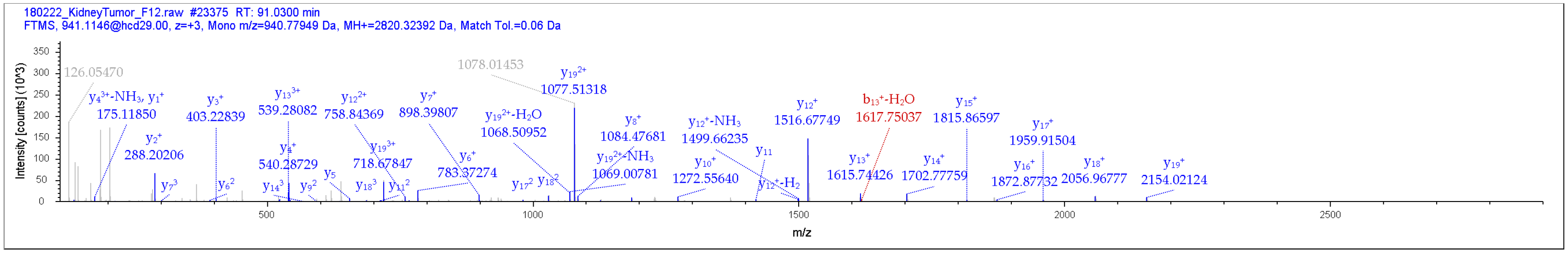

Sequence: TTVPPSGLSVPFSTWDQDHDLR, T2-Hex(1)HexNAc(1) (365.13220 Da)

Charge: +3,   Monoisotopic m/z: 940.77968 Da (+1.15 mmu/+1.22 ppm),   MH+: 2820.32447 Da,   RT: 90.7326 min,

Identified with: Sequest HT (v1.17); XCorr:5.61, Percolator q-Value:0.0e0, Percolator PEP:2.1e-8, Ions matched by search engine: 0/0

Fragment match tolerance used for search: 0.06 Da

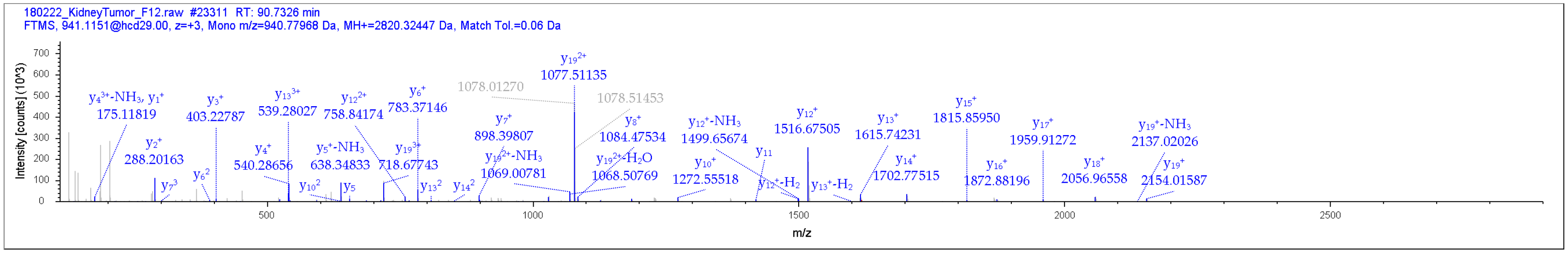

Sequence: TTVASLGAQ, T1-HexNAc (203.07937 Da), T2-Hex(1)HexNAc(1) (365.13220 Da)

Charge: +3,   Monoisotopic m/z: 472.55928 Da (-0.09 mmu/-0.18 ppm),   MH+: 1415.66328 Da,   RT: 27.1167 min,

Identified with: Sequest HT (v1.17); XCorr:1.74, Percolator q-Value:6.1e-4, Percolator PEP:4.5e-2, Ions matched by search engine: 0/0

Fragment match tolerance used for search: 0.06 Da

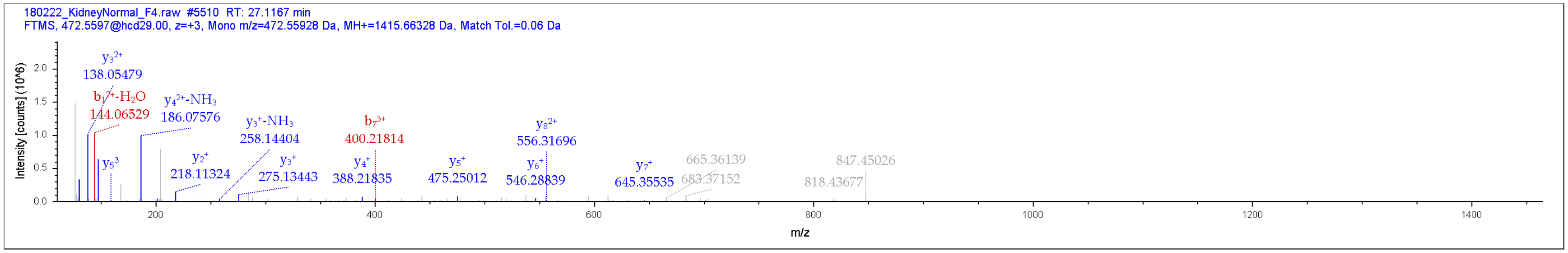

Sequence: TTTTSAGAEAAEGQFDR, T1-Hex(1)HexNAc(1) (365.13220 Da)

Charge: +3, Monoisotopic m/z: 693.30562 Da (-0.7 mmu/-1 ppm), MH+: 2077.90230 Da, RT: 48.5495 min,

Identified with: Sequest HT (v1.17); XCorr:5.16, Percolator q-Value:0.0e0, Percolator PEP:8.7e-4, Ions matched by search engine: 0/0

Fragment match tolerance used for search: 0.06 Da

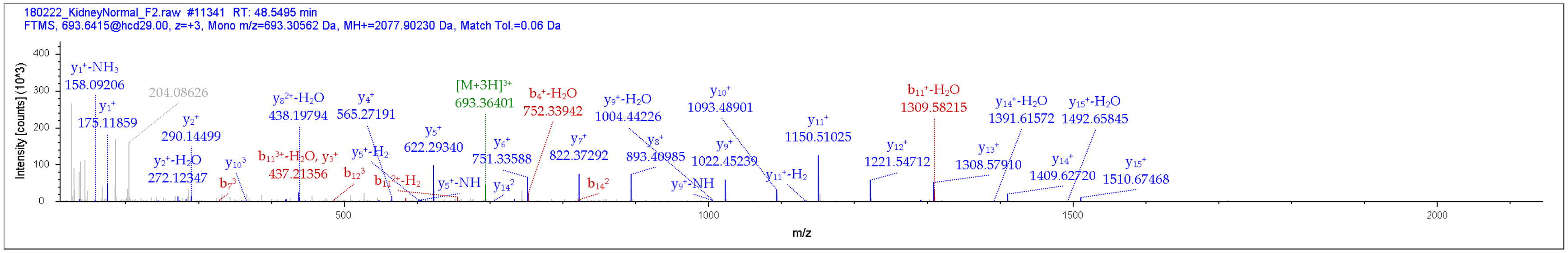

Sequence: TTTLLETVFHHV, T1-Hex(1)HexNAc(1) (365.13220 Da), T2-Hex(1)HexNAc(1) (365.13220 Da)

Charge: +3, Monoisotopic m/z: 710.00770 Da (+0.61 mmu/+0.86 ppm), MH+: 2128.00856 Da, RT: 95.1356 min,

Identified with: Sequest HT (v1.17); XCorr:2.68, Percolator q-Value:0.0e0, Percolator PEP:2.6e-3, Ions matched by search engine: 0/0

Fragment match tolerance used for search: 0.06 Da

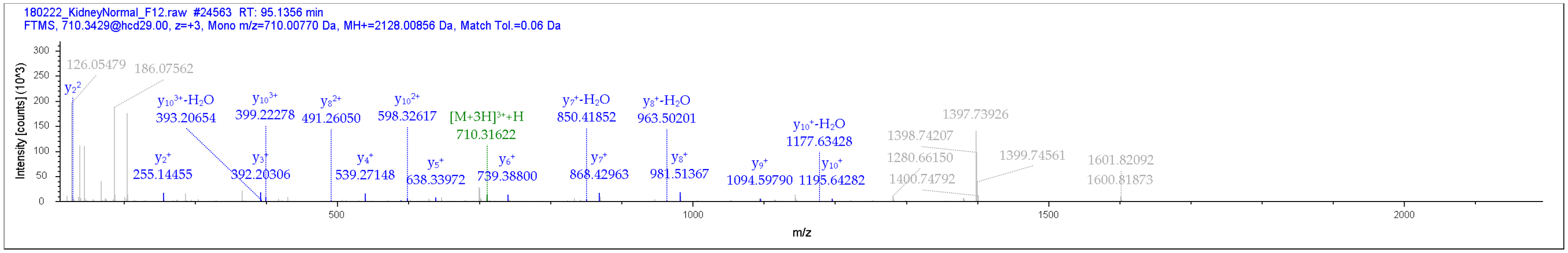

Sequence: TTTIAEPDPGMSGWPDGR, M11-Oxidation (15.99492 Da), T1-Hex(1)HexNAc(1) (365.13220 Da), T2-Hex(1)HexNAc(1) (365.13220 Da), T3-Hex(1)HexNAc(1) (365.13220 Da)

Charge: +4, Monoisotopic m/z: 750.56700 Da (+0.13 mmu/+0.17 ppm), MH+: 2999.24616 Da, RT: 54.4181 min,

Identified with: Sequest HT (v1.17); XCorr:4.00, Percolator q-Value:0.0e0, Percolator PEP:7.6e-6, Ions matched by search engine: 0/0

Fragment match tolerance used for search: 0.06 Da

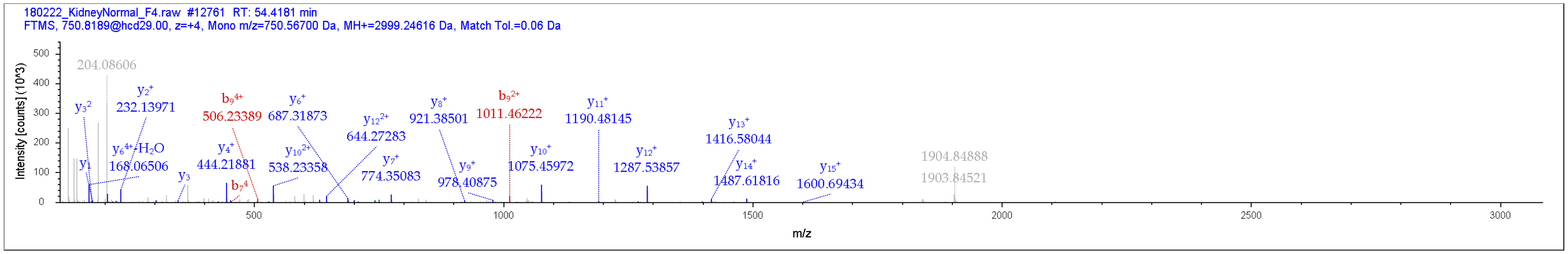

Sequence: TTSSGSGPFTDVR, T2-HexNAc (203.07937 Da), T1-Hex(1)HexNAc(1) (365.13220 Da)

Charge: +3, Monoisotopic m/z: 627.28210 Da (+0.88 mmu/+1.4 ppm), MH+: 1879.83174 Da, RT: 47.6298 min,

Identified with: Sequest HT (v1.17); XCorr:3.55, Percolator q-Value:0.0e0, Percolator PEP:5.4e-4, Ions matched by search engine: 0/0

Fragment match tolerance used for search: 0.06 Da

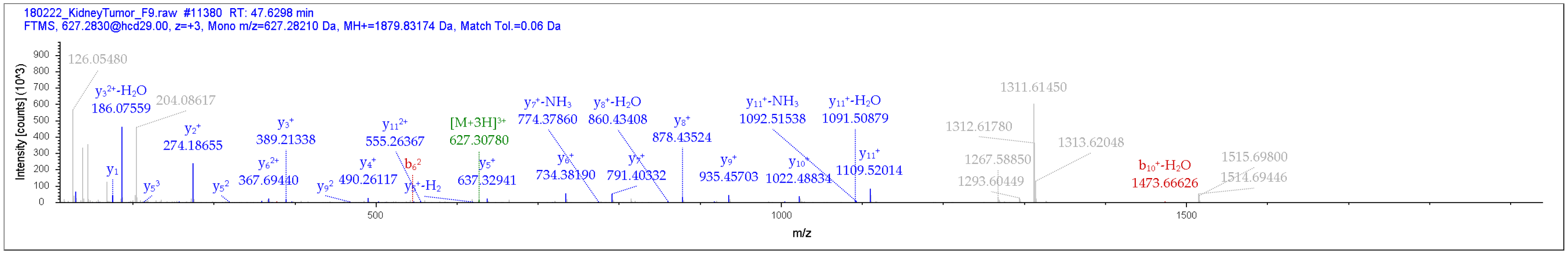

Sequence: TTSSGSGPFTDVR, T2-HexNAc (203.07937 Da), T1-Hex(1)HexNAc(1) (365.13220 Da)

Charge: +3,   Monoisotopic m/z: 627.28210 Da (+0.88 mmu/+1.4 ppm),   MH+: 1879.83174 Da,   RT: 47.6298 min,

Identified with: Sequest HT (v1.17); XCorr:3.55, Percolator q-Value:0.0e0, Percolator PEP:5.4e-4, Ions matched by search engine: 0/0

Fragment match tolerance used for search: 0.06 Da

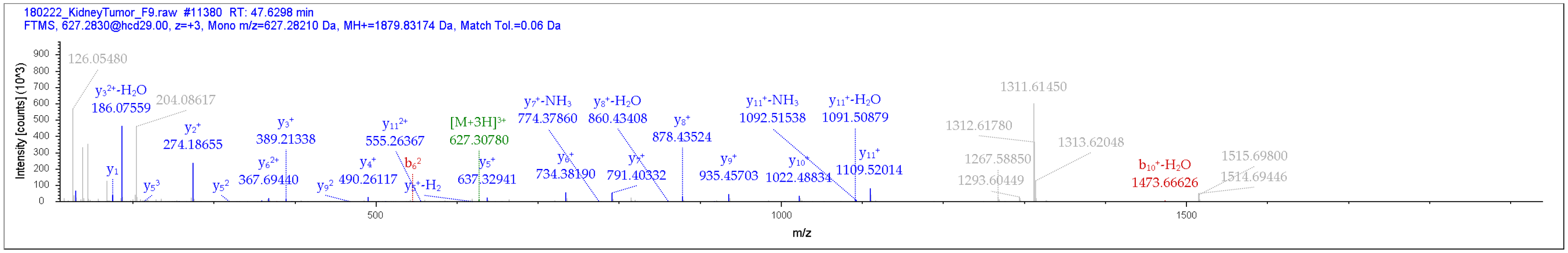

Sequence: TTQTPAPIQAPSAILPLPGQSVER, T4-Hex(1)HexNAc(1) (365.13220 Da)

Charge: +3,   Monoisotopic m/z: 946.49960 Da (+2.13 mmu/+2.26 ppm),   MH+: 2837.48426 Da,   RT: 95.9239 min,

Identified with: Sequest HT (v1.17); XCorr:4.32, Percolator q-Value:0.0e0, Percolator PEP:2.0e-6, Ions matched by search engine: 0/0

Fragment match tolerance used for search: 0.06 Da

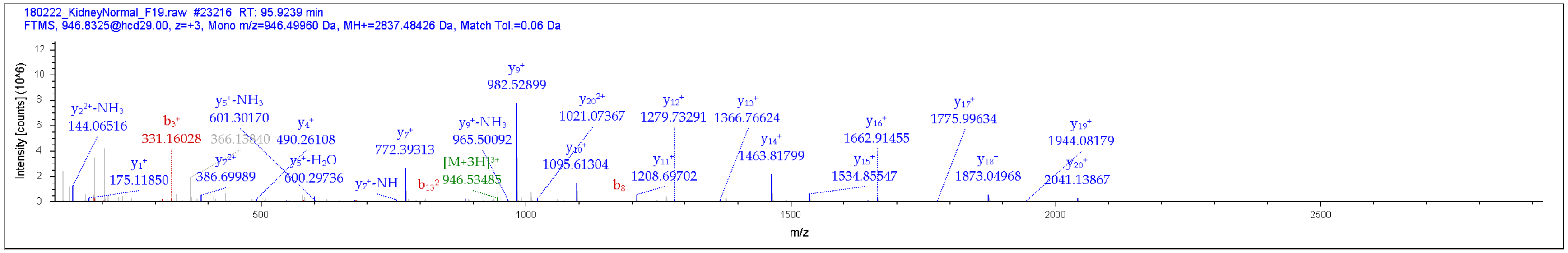

Sequence: TQAPTAQEAGEGPSGILELSGAH, T1-Hex(1)HexNAc(1) (365.13220 Da)

Charge: +3, Monoisotopic m/z: 862.74046 Da (+0.5 mmu/+0.58 ppm), MH+: 2586.20682 Da, RT: 79.0750 min,

Identified with: Sequest HT (v1.17); XCorr:7.16, Percolator q-Value:0.0e0, Percolator PEP:3.7e-7, Ions matched by search engine: 0/0

Fragment match tolerance used for search: 0.06 Da

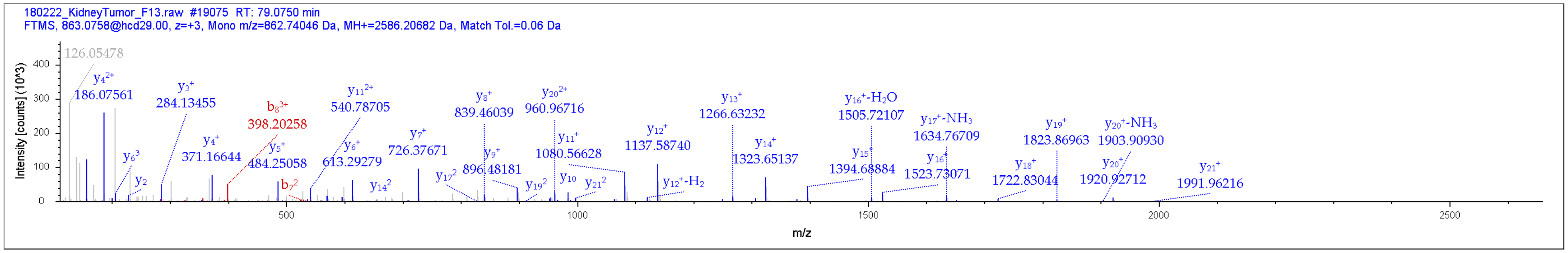

Sequence: TQAFGEWPWGQELGSR, T1-Hex(1)HexNAc(1) (365.13220 Da)

Charge: +3, Monoisotopic m/z: 738.67139 Da (+0.35 mmu/+0.48 ppm), MH+: 2213.99963 Da, RT: 98.9432 min,

Identified with: Sequest HT (v1.17); XCorr:3.64, Percolator q-Value:0.0e0, Percolator PEP:1.3e-4, Ions matched by search engine: 0/0

Fragment match tolerance used for search: 0.06 Da

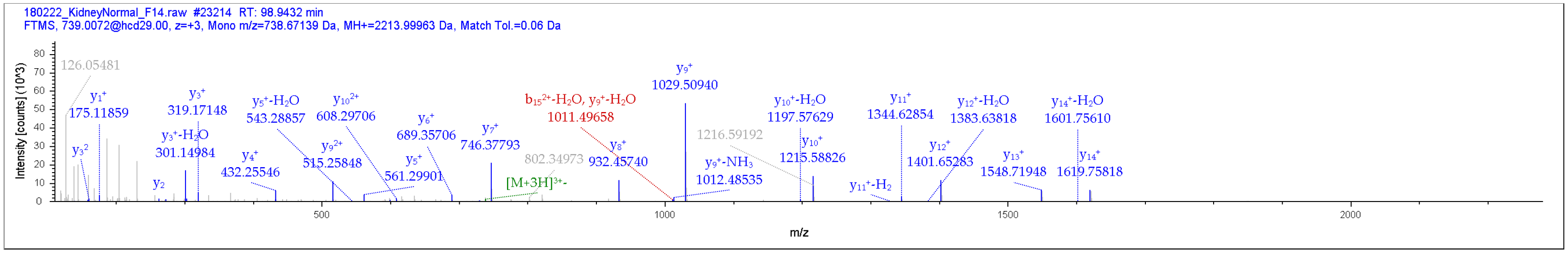

Sequence: TPVVQDCYHGDGQSYR, C7-Carbamidomethyl (57.02146 Da), T1-Hex(1)HexNAc(1) (365.13220 Da)

Charge: +3, Monoisotopic m/z: 749.65603 Da (+0.97 mmu/+1.29 ppm), MH+: 2246.95354 Da, RT: 39.3221 min,

Identified with: Sequest HT (v1.17); XCorr:4.79, Percolator q-Value:0.0e0, Percolator PEP:3.2e-5, Ions matched by search engine: 0/0

Fragment match tolerance used for search: 0.06 Da

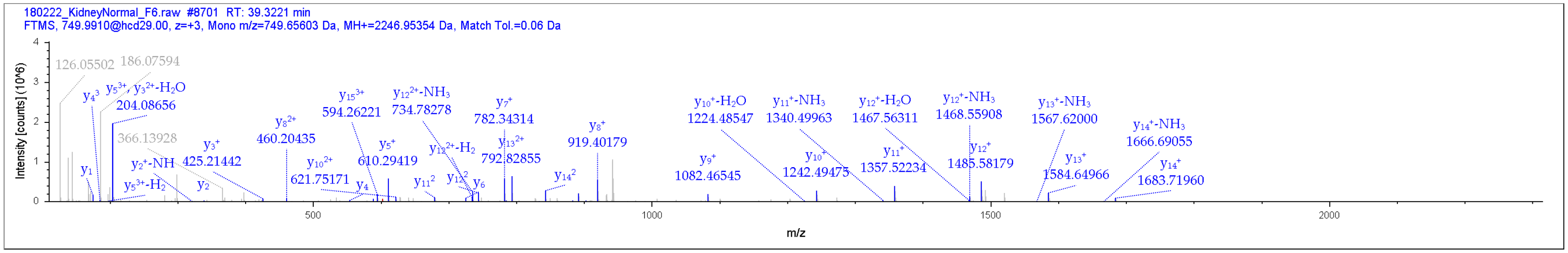

Sequence: TPVVDPDAPPSPPLGAPGLPPAGSPPD, S24-Hex(1)HexNAc(1) (365.13220 Da)

Charge: +3, Monoisotopic m/z: 960.80667 Da (+0.59 mmu/+0.61 ppm), MH+: 2880.40546 Da, RT: 91.4434 min,

Identified with: Sequest HT (v1.17); XCorr:3.67, Percolator q-Value:4.3e-3, Percolator PEP:1.4e-1, Ions matched by search engine: 0/0

Fragment match tolerance used for search: 0.06 Da

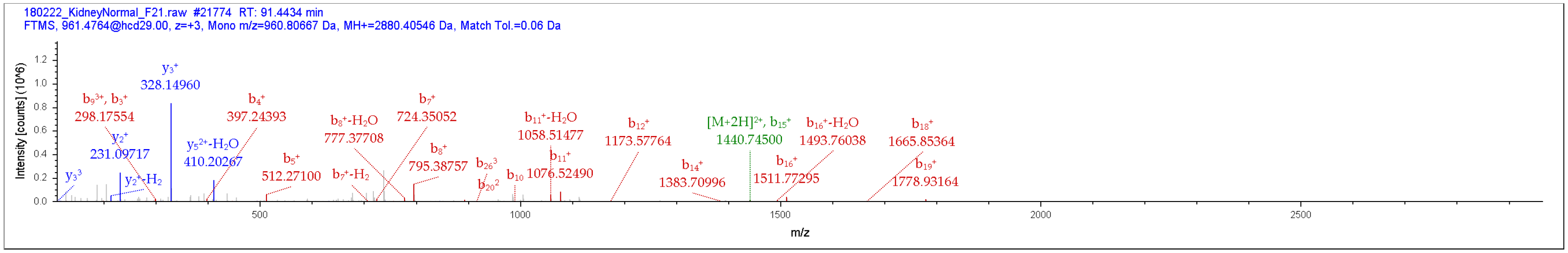

Sequence: TPVSAECPVVNELER, C7-Carbamidomethyl (57.02146 Da), T1-Hex(1)HexNAc(1) (365.13220 Da)

Charge: +3, Monoisotopic m/z: 688.99344 Da (+0.54 mmu/+0.78 ppm), MH+: 2064.96576 Da, RT: 68.9553 min,

Identified with: Sequest HT (v1.17); XCorr:3.51, Percolator q-Value:0.0e0, Percolator PEP:2.8e-4, Ions matched by search engine: 0/0

Fragment match tolerance used for search: 0.06 Da

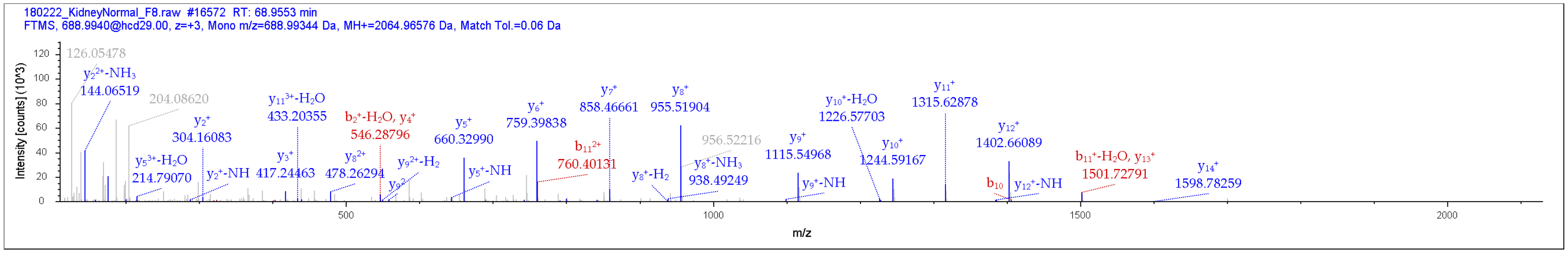

Sequence: TEPPTPPPPPTIPPAR, T5-HexNAc (203.07937 Da), T11-HexNAc (203.07937 Da), T1-Hex(1)HexNAc(1) (365.13220 Da)

Charge: +3, Monoisotopic m/z: 812.73643 Da (+1.05 mmu/+1.3 ppm), MH+: 2436.19473 Da, RT: 62.1815 min,

Identified with: Sequest HT (v1.17); XCorr:2.65, Percolator q-Value:2.5e-4, Percolator PEP:1.9e-2, Ions matched by search engine: 0/0

Fragment match tolerance used for search: 0.06 Da

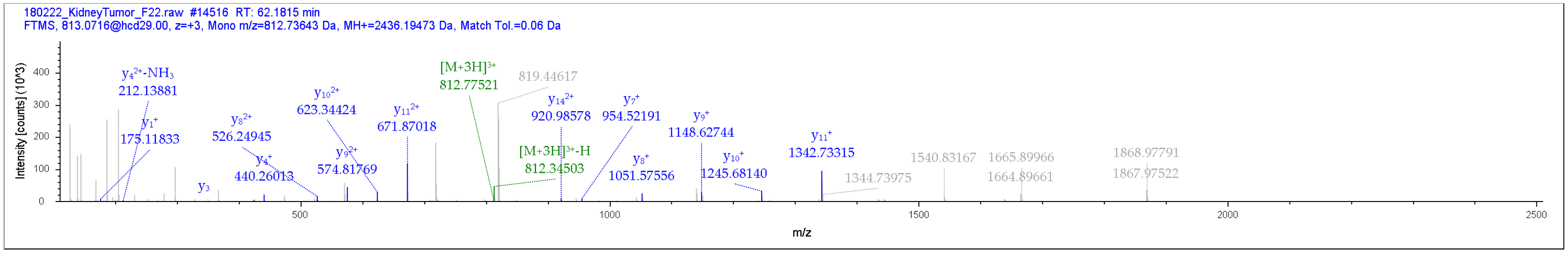

Sequence: TEHSSIHQPGVQEGLT, T16-Hex(1)HexNAc(1) (365.13220 Da)

Charge: +3, Monoisotopic m/z: 695.65872 Da (-0.08 mmu/-0.11 ppm), MH+: 2084.96162 Da, RT: 44.5535 min,

Identified with: Sequest HT (v1.17); XCorr:4.57, Percolator q-Value:1.5e-3, Percolator PEP:4.7e-2, Ions matched by search engine: 0/0

Fragment match tolerance used for search: 0.06 Da

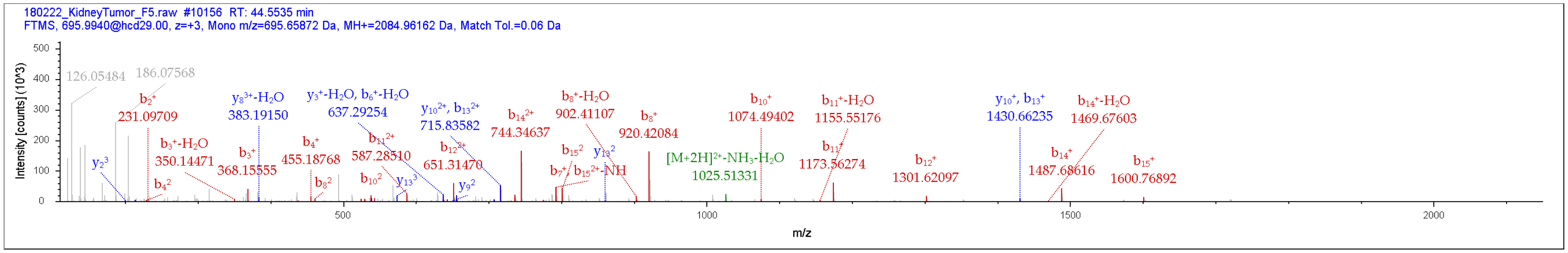

Sequence: TAAAALGPPAPVQAK, K15-Guanidinyl (42.02180 Da), T1-Hex(1)HexNAc(1) (365.13220 Da)

Charge: +2, Monoisotopic m/z: 885.46847 Da (+0.85 mmu/+0.96 ppm), MH+: 1769.92967 Da, RT: 53.5714 min,

Identified with: Sequest HT (v1.17); XCorr:2.62, Percolator q-Value:5.0e-5, Percolator PEP:2.7e-3, Ions matched by search engine: 0/0

Fragment match tolerance used for search: 0.06 Da

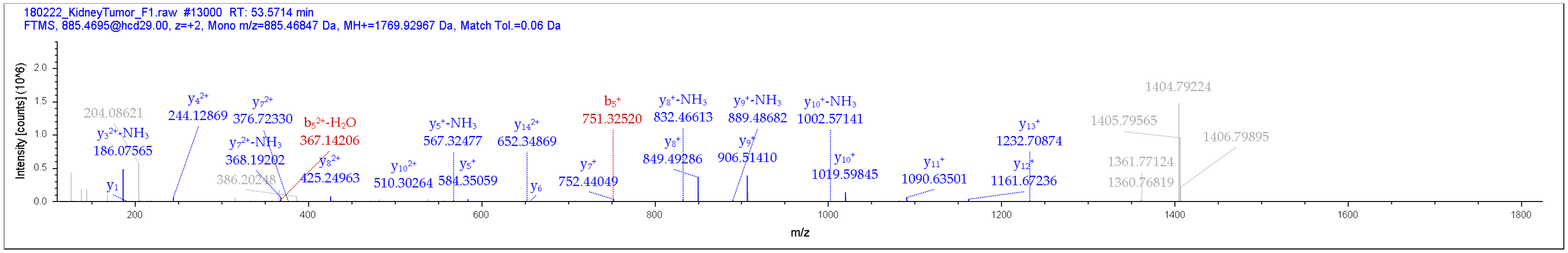

Sequence: SYPQEEATLNEMFR, S1-Hex(1)HexNAc(1) (365.13220 Da)

Charge: +3,   Monoisotopic m/z: 693.97423 Da (+0.61 mmu/+0.87 ppm),   MH+: 2079.90813 Da,   RT: 86.3205 min,

Identified with: Sequest HT (v1.17); XCorr:3.79, Percolator q-Value:0.0e0, Percolator PEP:2.0e-4, Ions matched by search engine: 0/0

Fragment match tolerance used for search: 0.06 Da

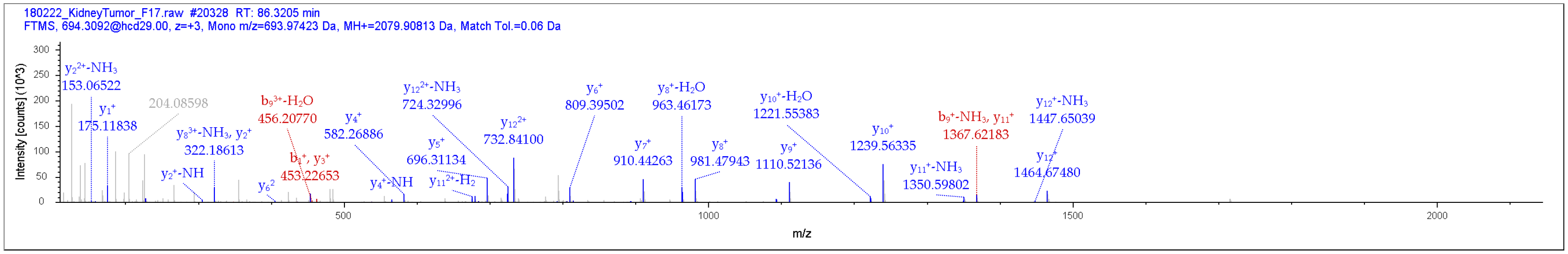

Sequence: SYPQEEATLNEMFR, M12-Oxidation (15.99492 Da), S1-Hex(1)HexNAc(1) (365.13220 Da)

Charge: +3,   Monoisotopic m/z: 699.30589 Da (+0.63 mmu/+0.9 ppm),   MH+: 2095.90311 Da,   RT: 65.9694 min,

Identified with: Sequest HT (v1.17); XCorr:3.83, Percolator q-Value:0.0e0, Percolator PEP:4.2e-4, Ions matched by search engine: 0/0

Fragment match tolerance used for search: 0.06 Da

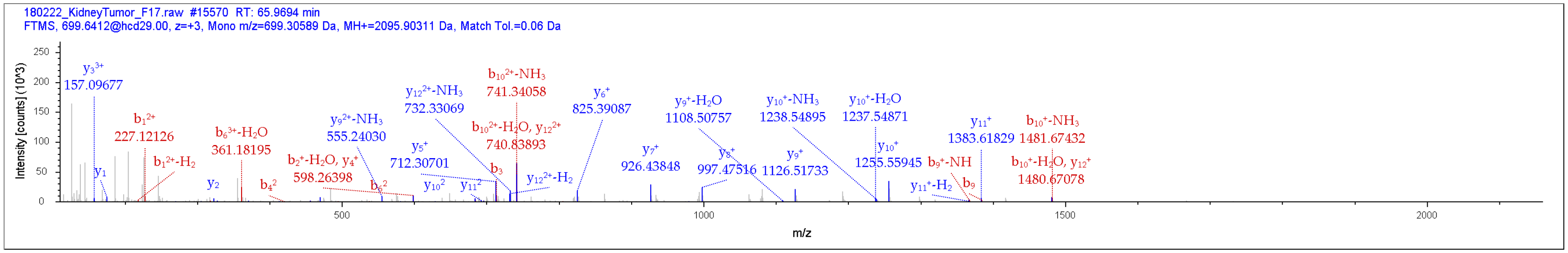

Sequence: SYPASGHTTPLSR, S1-Hex(1)HexNAc(1) (365.13220 Da)

Charge: +3,   Monoisotopic m/z: 580.27513 Da (-0.72 mmu/-1.24 ppm),   MH+: 1738.81084 Da,   RT: 37.0239 min,

Identified with: Sequest HT (v1.17); XCorr:3.39, Percolator q-Value:0.0e0, Percolator PEP:2.4e-4, Ions matched by search engine: 0/0

Fragment match tolerance used for search: 0.06 Da

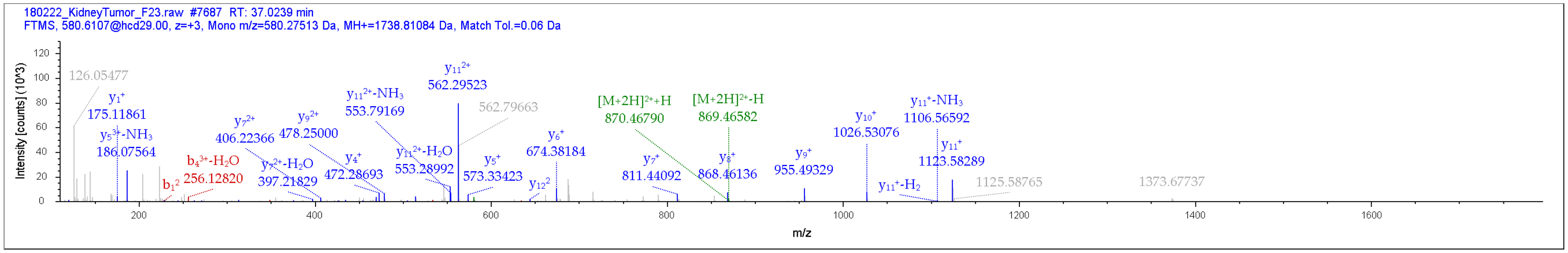

Sequence: SVRPQDAGTYVCTATNR, C12-Carbamidomethyl (57.02146 Da), S1-Hex(1)HexNAc(1) (365.13220 Da)

Charge: +3,   Monoisotopic m/z: 754.35019 Da (+0.33 mmu/+0.44 ppm),   MH+: 2261.03603 Da,   RT: 47.6387 min,

Identified with: Sequest HT (v1.17); XCorr:3.45, Percolator q-Value:0.0e0, Percolator PEP:5.4e-4, Ions matched by search engine: 0/0

Fragment match tolerance used for search: 0.06 Da

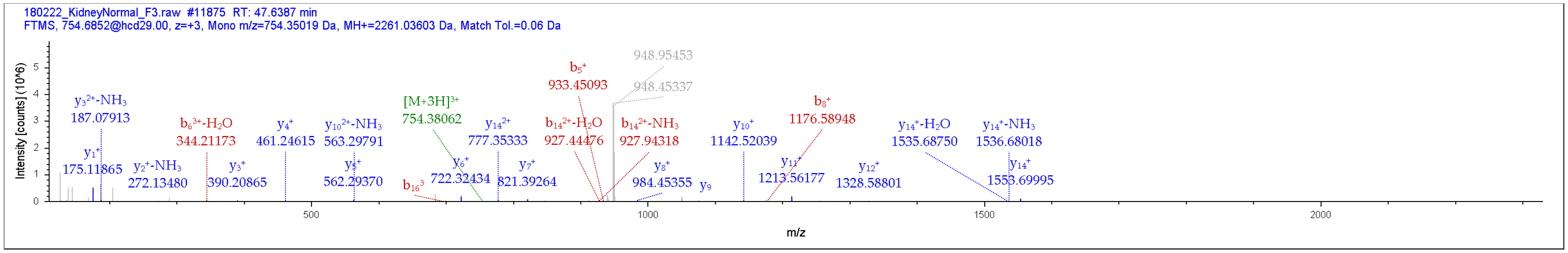

Sequence: SVRPLPCGPQEAACR, C7-Carbamidomethyl (57.02146 Da), C14-Carbamidomethyl (57.02146 Da), S1-Hex(1)HexNAc(1) (365.13220 Da)

Charge: +4, Monoisotopic m/z: 516.49147 Da (-2.3 mmu/-4.45 ppm), MH+: 2062.94404 Da, RT: 43.1550 min,

Identified with: Sequest HT (v1.17); XCorr:2.94, Percolator q-Value:2.5e-4, Percolator PEP:2.0e-2, Ions matched by search engine: 0/0

Fragment match tolerance used for search: 0.06 Da

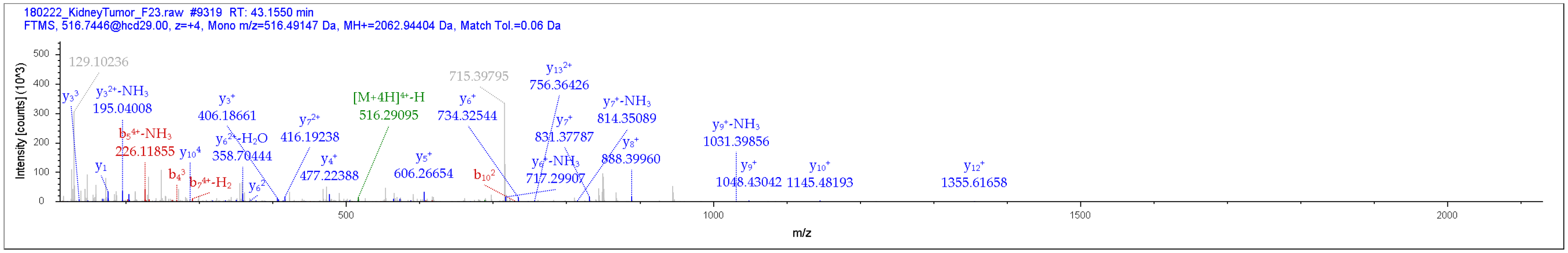

Sequence: SVRPLPCGPQEAACR, C7-Carbamidomethyl (57.02146 Da), C14-Carbamidomethyl (57.02146 Da), S1-Hex(1)HexNAc(1) (365.13220 Da)

Charge: +2, Monoisotopic m/z: 1031.98054 Da (+0.3 mmu/+0.29 ppm), MH+: 2062.95381 Da, RT: 42.5588 min,

Identified with: Sequest HT (v1.17); XCorr:2.14, Percolator q-Value:0.0e0, Percolator PEP:2.1e-3, Ions matched by search engine: 0/0

Fragment match tolerance used for search: 0.06 Da

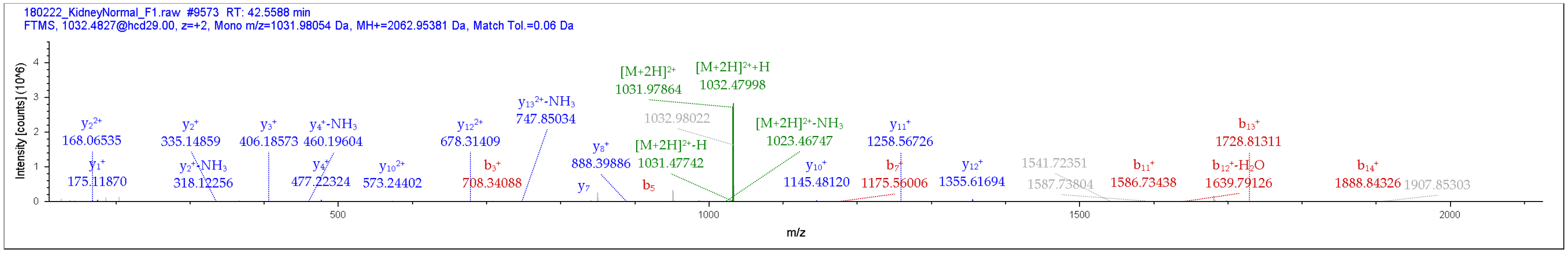

Sequence: SVRPLPCGPQEAACR, C7-Carbamidomethyl (57.02146 Da), C14-Carbamidomethyl (57.02146 Da), S1-HexNAc (203.07937 Da)

Charge: +3, Monoisotopic m/z: 634.30539 Da (+0.41 mmu/+0.64 ppm), MH+: 1900.90161 Da, RT: 44.8190 min,

Identified with: Sequest HT (v1.17); XCorr:3.90, Percolator q-Value:0.0e0, Percolator PEP:5.2e-5, Ions matched by search engine: 0/0

Fragment match tolerance used for search: 0.06 Da

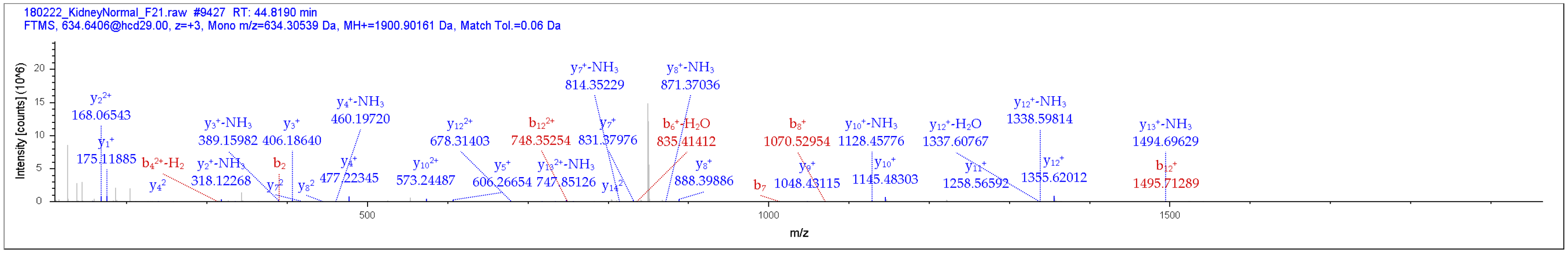

Sequence: SVRPLPCGPQEAACR, C7-Carbamidomethyl (57.02146 Da), C14-Carbamidomethyl (57.02146 Da), S1-HexNAc (203.07937 Da)

Charge: +3, Monoisotopic m/z: 634.30426 Da (-0.72 mmu/-1.14 ppm), MH+: 1900.89823 Da, RT: 43.2919 min,

Identified with: Sequest HT (v1.17); XCorr:3.93, Percolator q-Value:0.0e0, Percolator PEP:8.9e-5, Ions matched by search engine: 0/0

Fragment match tolerance used for search: 0.06 Da

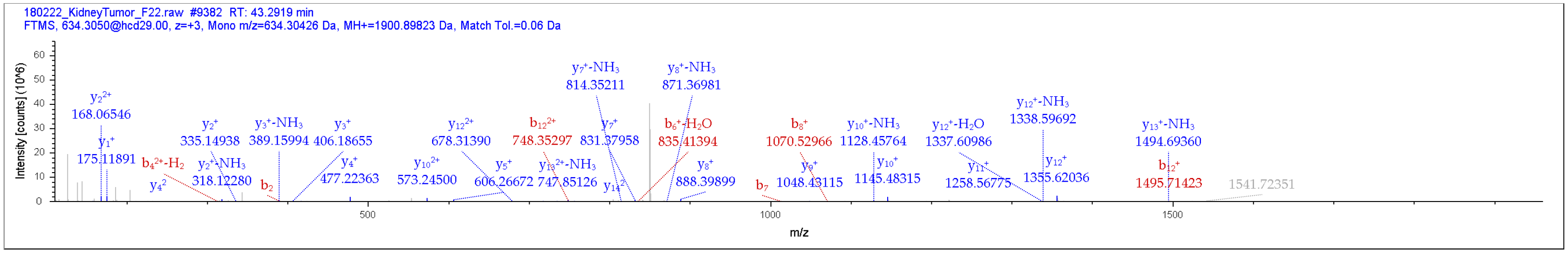

Sequence: SVRPLPCGPQEAACR, C7-Carbamidomethyl (57.02146 Da), C14-Carbamidomethyl (57.02146 Da), S1-Hex(1)HexNAc(1) (365.13220 Da)

Charge: +3, Monoisotopic m/z: 688.32248 Da (-0.11 mmu/-0.16 ppm), MH+: 2062.95289 Da, RT: 43.6830 min,

Identified with: Sequest HT (v1.17); XCorr:2.80, Percolator q-Value:0.0e0, Percolator PEP:2.1e-5, Ions matched by search engine: 0/0

Fragment match tolerance used for search: 0.06 Da

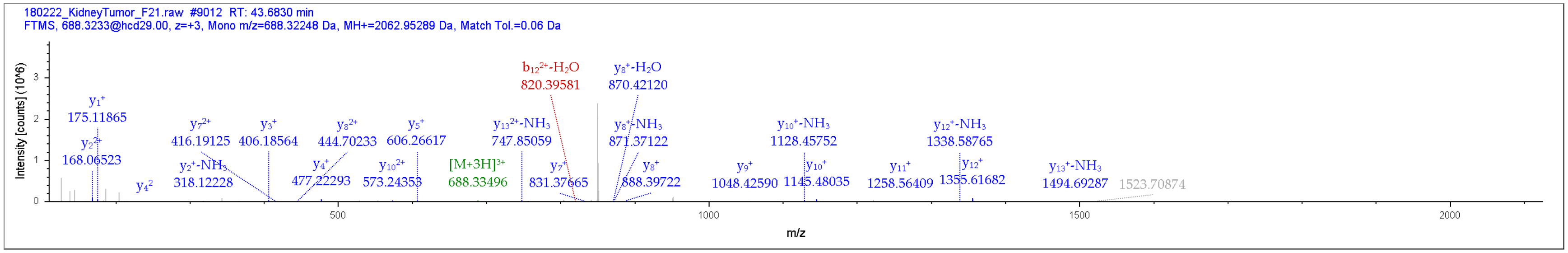

Sequence: SVRPLPCGPQEAACR, C7-Carbamidomethyl (57.02146 Da), C14-Carbamidomethyl (57.02146 Da), S1-HexNAc (203.07937 Da)

Charge: +3, Monoisotopic m/z: 634.30701 Da (+2.03 mmu/+3.2 ppm), MH+: 1900.90647 Da, RT: 41.4657 min,

Identified with: Sequest HT (v1.17); XCorr:4.09, Percolator q-Value:0.0e0, Percolator PEP:7.6e-4, Ions matched by search engine: 0/0

Fragment match tolerance used for search: 0.06 Da

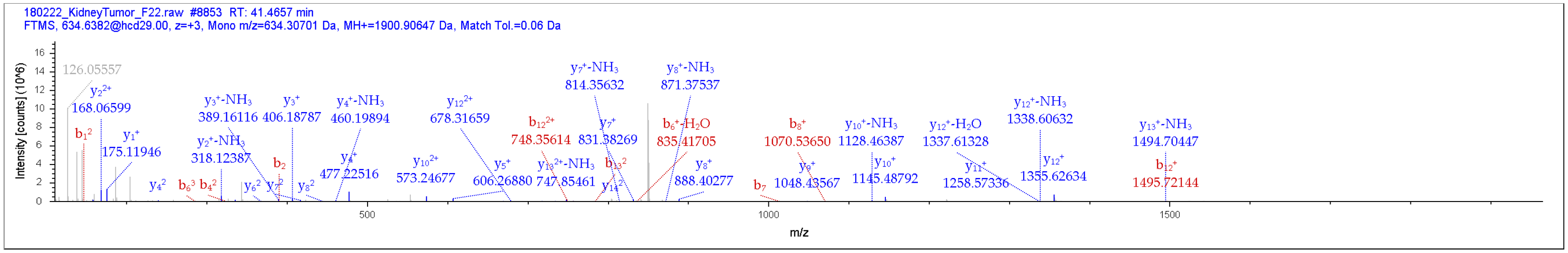

Sequence: SVPSEDEVLGEPSQDILVIDQTR, S1-Hex(1)HexNAc(1) (365.13220 Da)

Charge: +3,   Monoisotopic m/z: 964.46799 Da (+0.08 mmu/+0.08 ppm),   MH+: 2891.38941 Da,   RT: 93.5451 min,

Identified with: Sequest HT (v1.17); XCorr:4.76, Percolator q-Value:0.0e0, Percolator PEP:2.6e-5, Ions matched by search engine: 0/0

Fragment match tolerance used for search: 0.06 Da

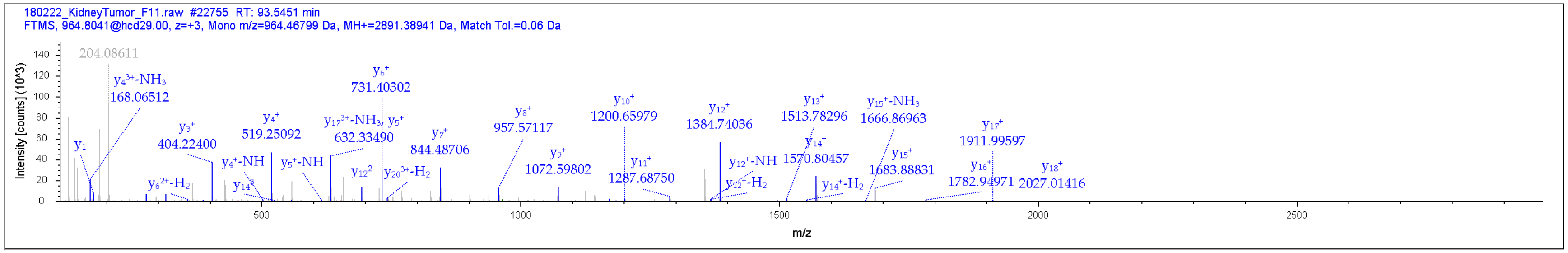

Sequence: SVPPYQHGVSVEVNCR, C15-Carbamidomethyl (57.02146 Da), S1-Hex(1)HexNAc(1) (365.13220 Da)

Charge: +3,   Monoisotopic m/z: 731.67757 Da (+1.77 mmu/+2.42 ppm),   MH+: 2193.01815 Da,   RT: 51.6835 min,

Identified with: Sequest HT (v1.17); XCorr:4.05, Percolator q-Value:0.0e0, Percolator PEP:8.1e-5, Ions matched by search engine: 0/0

Fragment match tolerance used for search: 0.06 Da

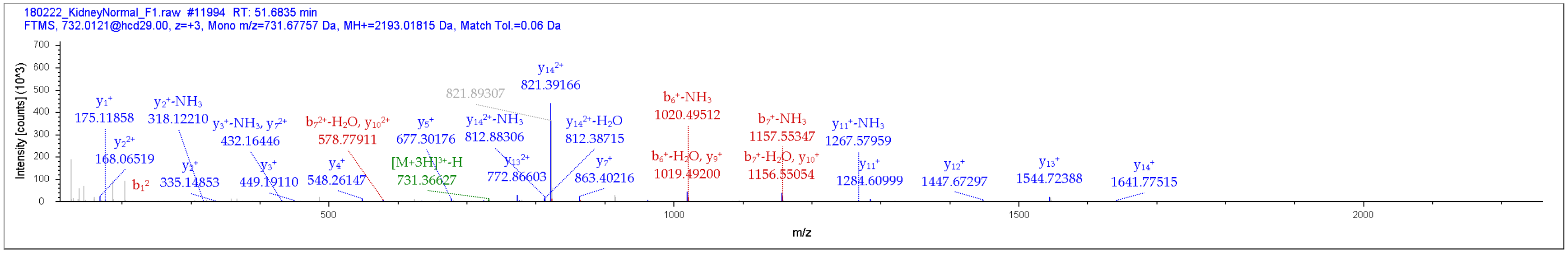

Sequence: SVPPYQHGVSVEVNCR, C15-Carbamidomethyl (57.02146 Da), S1-Hex(1)HexNAc(1) (365.13220 Da)

Charge: +3, Monoisotopic m/z: 731.67538 Da (-0.42 mmu/-0.57 ppm), MH+: 2193.01159 Da, RT: 51.7280 min,

Identified with: Sequest HT (v1.17); XCorr:4.39, Percolator q-Value:0.0e0, Percolator PEP:2.3e-5, Ions matched by search engine: 0/0

Fragment match tolerance used for search: 0.06 Da

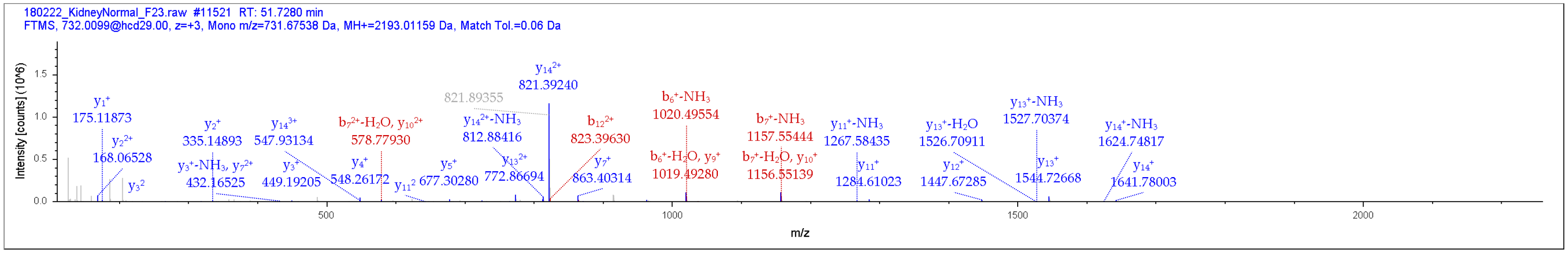

Sequence: SVPALGVTPTVR, S1-Hex(1)HexNAc(1) (365.13220 Da)

Charge: +3, Monoisotopic m/z: 521.28221 Da (+0.05 mmu/+0.09 ppm), MH+: 1561.83208 Da, RT: 62.2974 min,

Identified with: Sequest HT (v1.17); XCorr:2.74, Percolator q-Value:0.0e0, Percolator PEP:2.1e-3, Ions matched by search engine: 0/0

Fragment match tolerance used for search: 0.06 Da

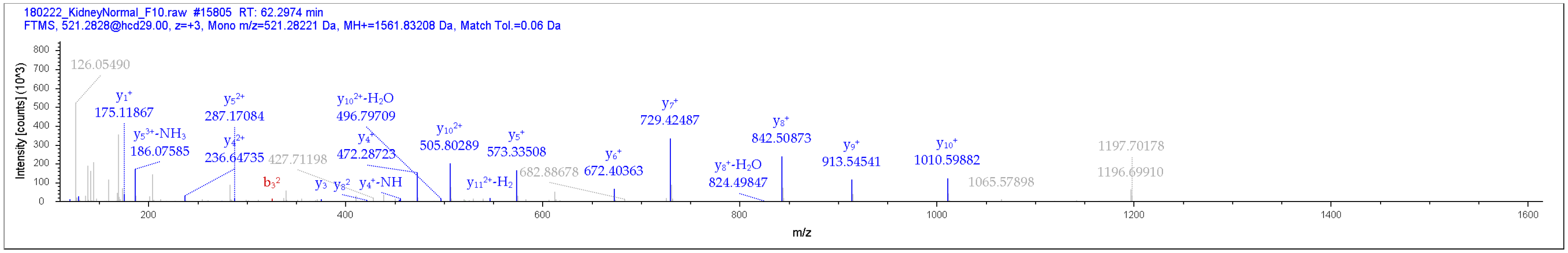

Sequence: SVMSPQDSFK, S1-HexNAc (203.07937 Da), K10-Guanidinyl (42.02180 Da)

Charge: +3, Monoisotopic m/z: 457.54658 Da (-0.16 mmu/-0.35 ppm), MH+: 1370.62518 Da, RT: 53.5138 min,

Identified with: Sequest HT (v1.17); XCorr:2.86, Percolator q-Value:1.6e-3, Percolator PEP:4.9e-2, Ions matched by search engine: 0/0

Fragment match tolerance used for search: 0.06 Da

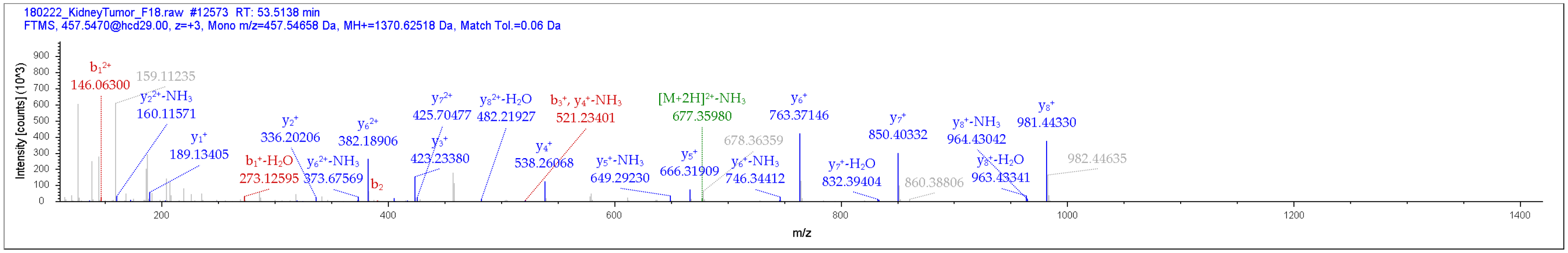

Sequence: SVMSPQDSFK, M3-Oxidation (15.99492 Da), S1-HexNAc (203.07937 Da), K10-Guanidinyl (42.02180 Da)

Charge: +3, Monoisotopic m/z: 462.87799 Da (-0.38 mmu/-0.83 ppm), MH+: 1386.61942 Da, RT: 44.1843 min,

Identified with: Sequest HT (v1.17); XCorr:2.57, Percolator q-Value:3.2e-4, Percolator PEP:2.1e-2, Ions matched by search engine: 0/0

Fragment match tolerance used for search: 0.06 Da

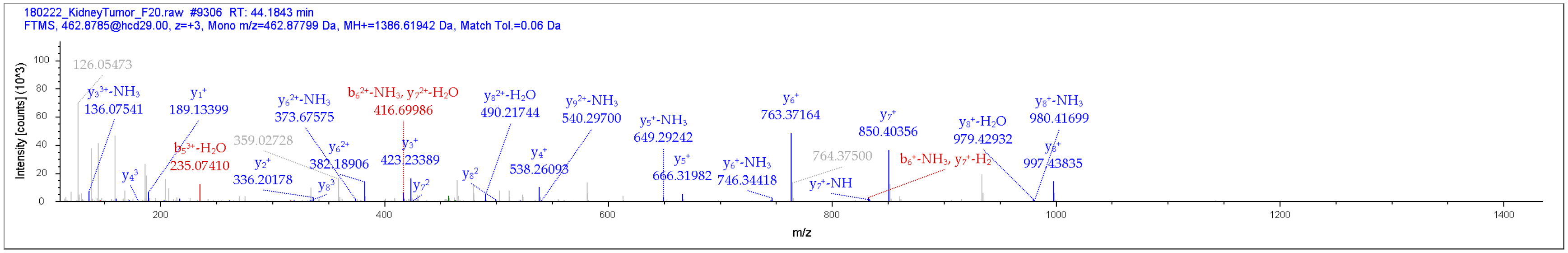

Sequence: SVMSPQDSFK, M3-Oxidation (15.99492 Da), S1-HexNAc (203.07937 Da), K10-Guanidinyl (42.02180 Da)

Charge: +3, Monoisotopic m/z: 462.87787 Da (-0.51 mmu/-1.1 ppm), MH+: 1386.61905 Da, RT: 41.8325 min,

Identified with: Sequest HT (v1.17); XCorr:2.73, Percolator q-Value:2.5e-3, Percolator PEP:6.7e-2, Ions matched by search engine: 0/0

Fragment match tolerance used for search: 0.06 Da

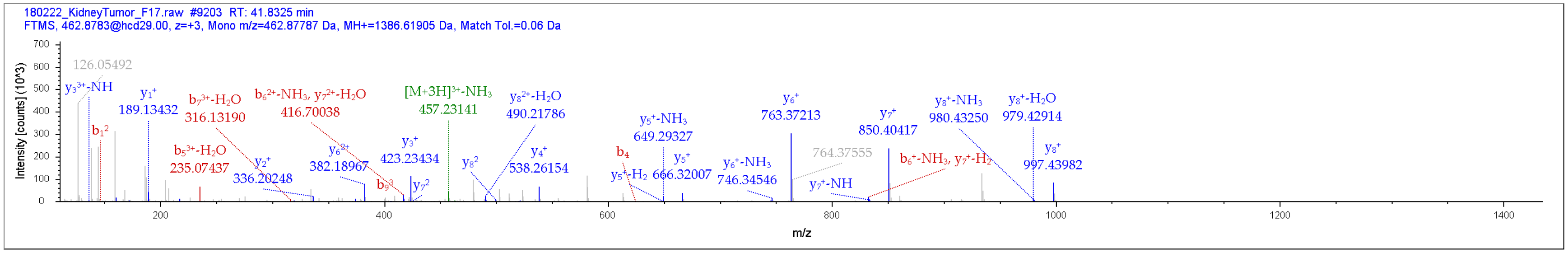

Sequence: SVGQQMIFEEHGFR, S1-Hex(1)HexNAc(1) (365.13220 Da)

Charge: +3, Monoisotopic m/z: 677.31116 Da (+0.59 mmu/+0.88 ppm), MH+: 2029.91893 Da, RT: 69.7721 min,

Identified with: Sequest HT (v1.17); XCorr:4.36, Percolator q-Value:0.0e0, Percolator PEP:1.1e-4, Ions matched by search engine: 0/0

Fragment match tolerance used for search: 0.06 Da

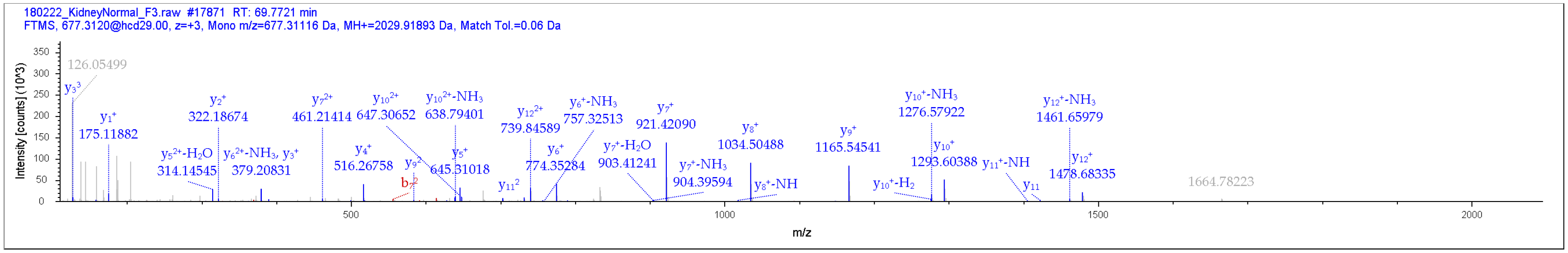

Sequence: SVGQQMIFEEHGFR, S1-Hex(1)HexNAc(1) (365.13220 Da)

Charge: +3, Monoisotopic m/z: 677.30992 Da (-0.65 mmu/-0.96 ppm), MH+: 2029.91519 Da, RT: 69.3487 min,

Identified with: Sequest HT (v1.17); XCorr:4.35, Percolator q-Value:0.0e0, Percolator PEP:3.7e-4, Ions matched by search engine: 0/0

Fragment match tolerance used for search: 0.06 Da

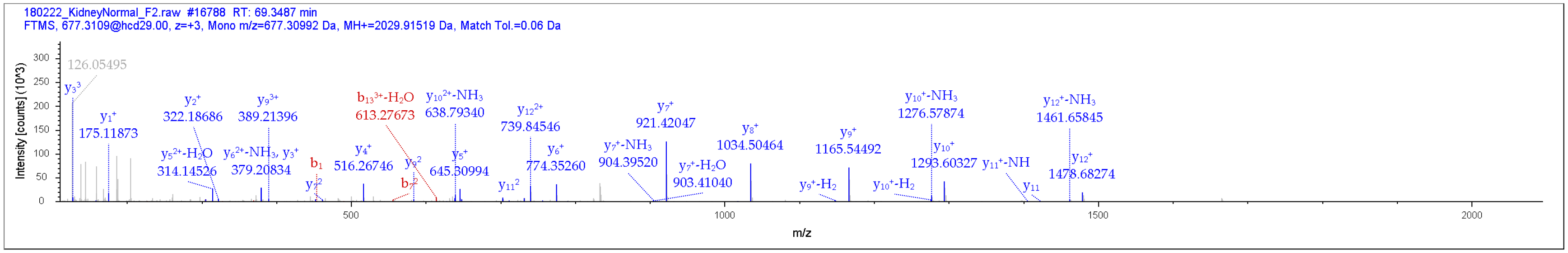

Sequence: SVGQQMIFEEHGFR, M6-Oxidation (15.99492 Da), S1-Hex(1)HexNAc(1) (365.13220 Da)

Charge: +3, Monoisotopic m/z: 682.64259 Da (+0.38 mmu/+0.56 ppm), MH+: 2045.91320 Da, RT: 61.5459 min,

Identified with: Sequest HT (v1.17); XCorr:4.06, Percolator q-Value:0.0e0, Percolator PEP:3.6e-4, Ions matched by search engine: 0/0

Fragment match tolerance used for search: 0.06 Da

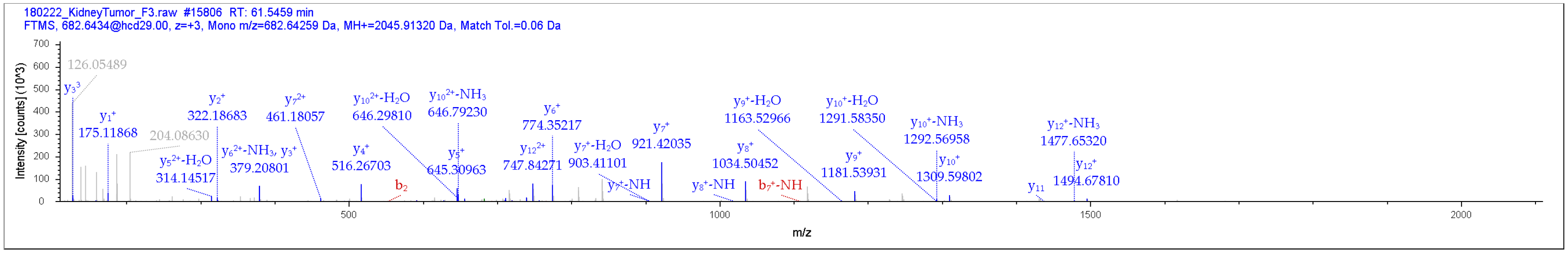

Sequence: SVGQQMIFEEHGFR, M6-Oxidation (15.99492 Da), S1-Hex(1)HexNAc(1) (365.13220 Da)

Charge: +2, Monoisotopic m/z: 1023.45924 Da (-0.43 mmu/-0.42 ppm), MH+: 2045.91120 Da, RT: 61.6403 min,

Identified with: Sequest HT (v1.17); XCorr:2.80, Percolator q-Value:0.0e0, Percolator PEP:1.1e-3, Ions matched by search engine: 0/0

Fragment match tolerance used for search: 0.06 Da

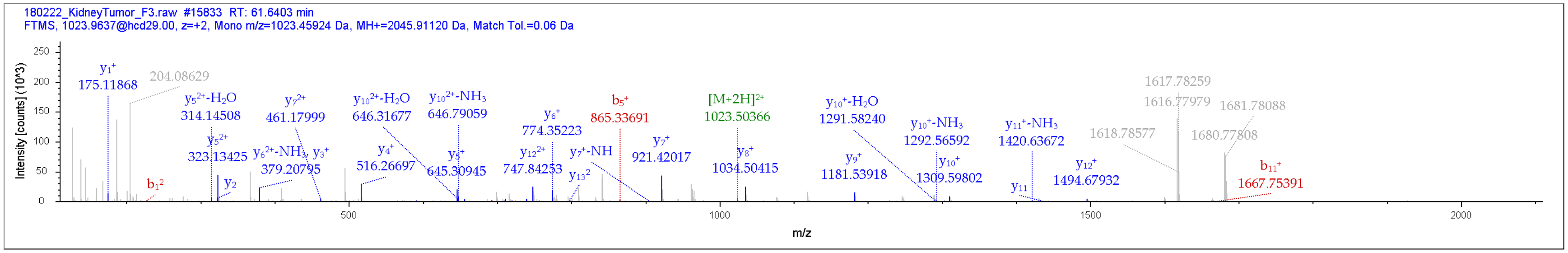

Sequence: SVGAAAGPVVPPCPGR, C13-Carbamidomethyl (57.02146 Da), S1-HexNAc (203.07937 Da)

Charge: +3, Monoisotopic m/z: 565.62215 Da (-0.38 mmu/-0.66 ppm), MH+: 1694.85191 Da, RT: 53.2991 min,

Identified with: Sequest HT (v1.17); XCorr:4.25, Percolator q-Value:0.0e0, Percolator PEP:5.0e-3, Ions matched by search engine: 0/0

Fragment match tolerance used for search: 0.06 Da

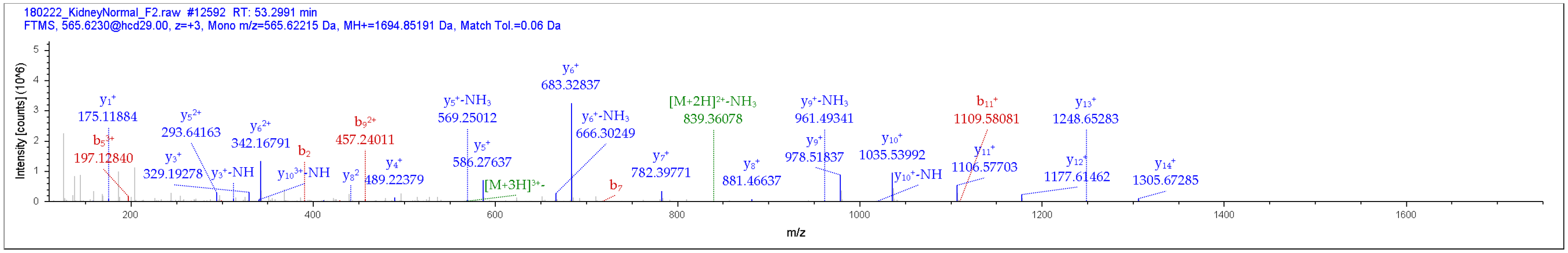

Sequence: SVGAAAGPVVPPCPGR, C13-Carbamidomethyl (57.02146 Da), S1-HexNAc (203.07937 Da)

Charge: +3, Monoisotopic m/z: 565.62191 Da (-0.62 mmu/-1.09 ppm), MH+: 1694.85118 Da, RT: 54.2593 min,

Identified with: Sequest HT (v1.17); XCorr:4.25, Percolator q-Value:5.0e-5, Percolator PEP:5.5e-3, Ions matched by search engine: 0/0

Fragment match tolerance used for search: 0.06 Da

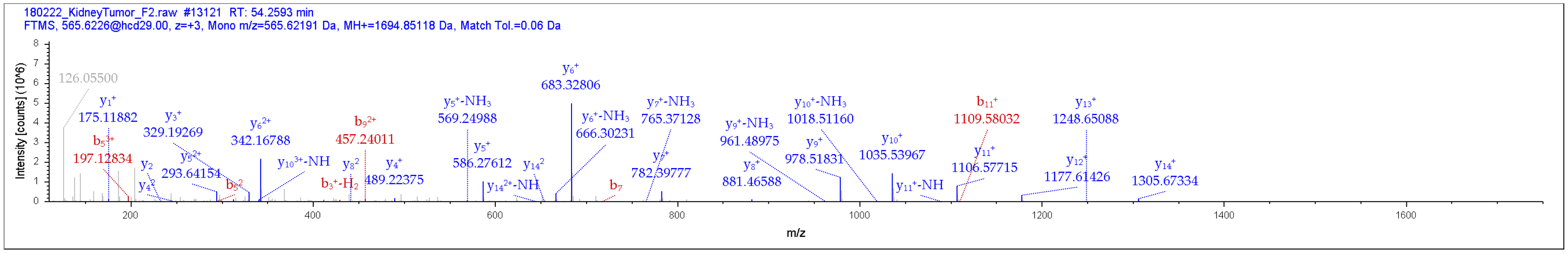

Sequence: SVGAAAGPVVPPCPGR, C13-Carbamidomethyl (57.02146 Da), S1-Hex(1)HexNAc(1) (365.13220 Da)

Charge: +3, Monoisotopic m/z: 619.64023 Da (+0.1 mmu/+0.16 ppm), MH+: 1856.90615 Da, RT: 52.9240 min,

Identified with: Sequest HT (v1.17); XCorr:3.49, Percolator q-Value:1.1e-4, Percolator PEP:9.5e-3, Ions matched by search engine: 0/0

Fragment match tolerance used for search: 0.06 Da

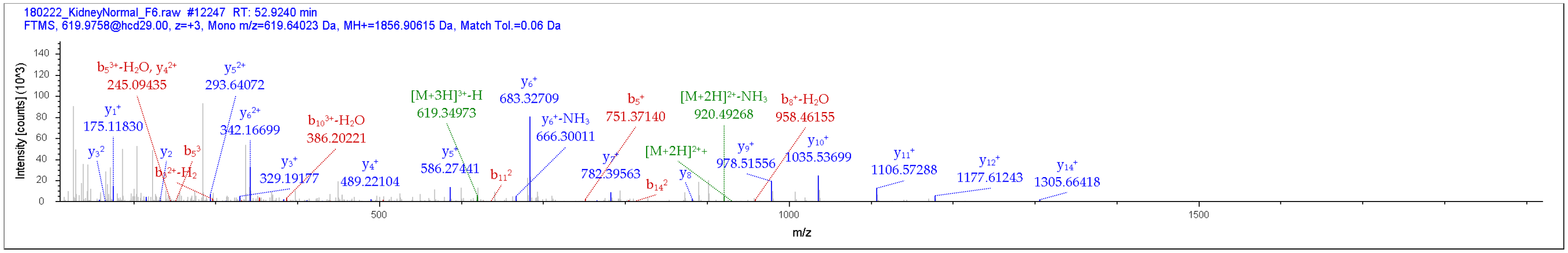

Sequence: SVGAAAGPVVPPCPGR, C13-Carbamidomethyl (57.02146 Da), S1-Hex(1)HexNAc(1) (365.13220 Da)

Charge: +3,   Monoisotopic m/z: 619.64018 Da (+0.05 mmu/+0.08 ppm),   MH+: 1856.90600 Da,   RT: 56.0416 min,

Identified with: Sequest HT (v1.17); XCorr:3.99, Percolator q-Value:5.0e-5, Percolator PEP:2.9e-3, Ions matched by search engine: 0/0

Fragment match tolerance used for search: 0.06 Da

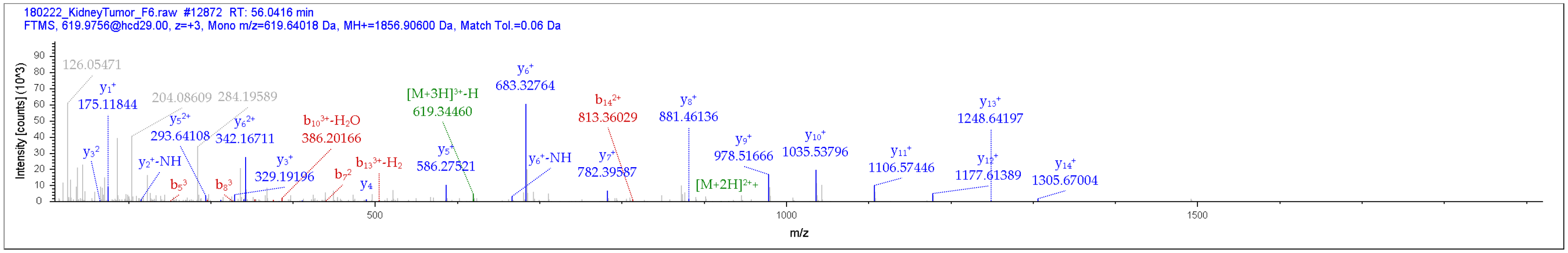

Sequence: SVGAAAGPVVPPCPGR, C13-Carbamidomethyl (57.02146 Da), S1-HexNAc (203.07937 Da)

Charge: +3,   Monoisotopic m/z: 565.62229 Da (-0.24 mmu/-0.42 ppm),   MH+: 1694.85232 Da,   RT: 53.8347 min,

Identified with: Sequest HT (v1.17); XCorr:4.29, Percolator q-Value:0.0e0, Percolator PEP:4.8e-3, Ions matched by search engine: 0/0

Fragment match tolerance used for search: 0.06 Da

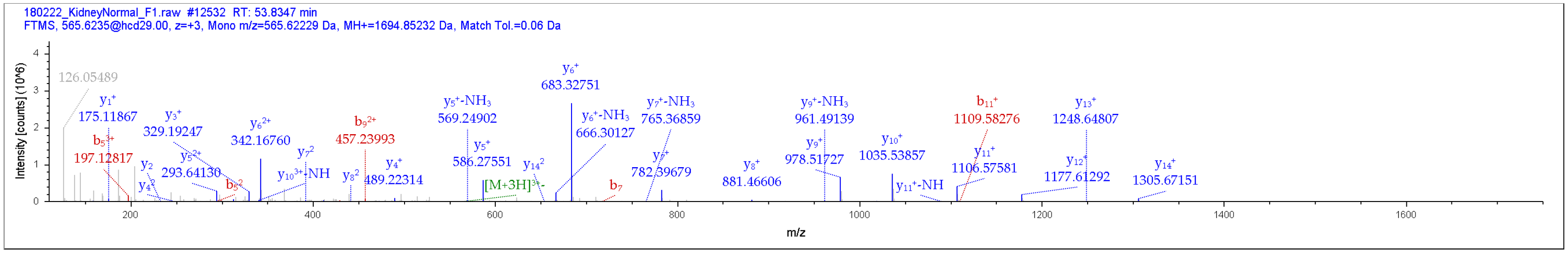

Sequence: SVGAAAGPVVPPCPGR, C13-Carbamidomethyl (57.02146 Da), S1-Hex(1)HexNAc(1) (365.13220 Da)

Charge: +2,   Monoisotopic m/z: 928.95698 Da (+0.41 mmu/+0.44 ppm),   MH+: 1856.90668 Da,   RT: 55.2459 min,

Identified with: Sequest HT (v1.17); XCorr:2.42, Percolator q-Value:0.0e0, Percolator PEP:1.4e-4, Ions matched by search engine: 0/0

Fragment match tolerance used for search: 0.06 Da

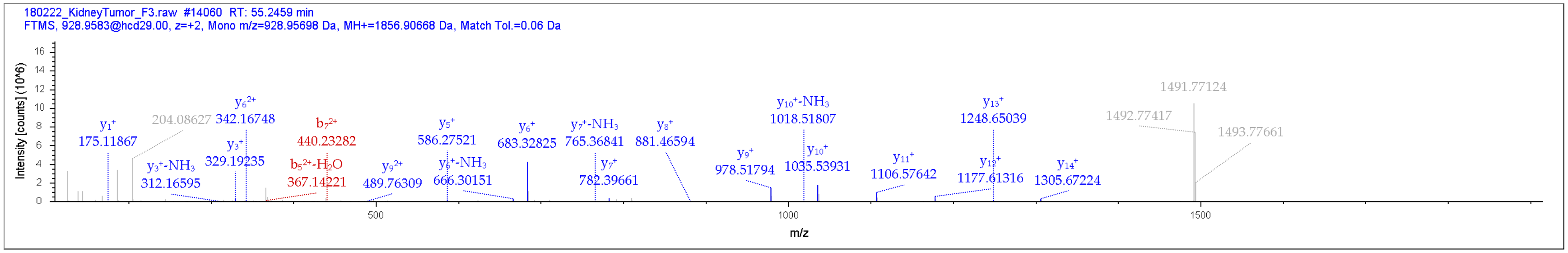

Sequence: SVFPAQDKPGELQATLDHTGHTSSK, K8-Guanidinyl (42.02180 Da), K25-Guanidinyl (42.02180 Da), S1-Hex(1)HexNAc(1) (365.13220 Da)

Charge: +5,   Monoisotopic m/z: 620.90307 Da (+0.9 mmu/+1.45 ppm),   MH+: 3100.48627 Da,   RT: 66.6775 min,

Identified with: Sequest HT (v1.17); XCorr:4.94, Percolator q-Value:0.0e0, Percolator PEP:1.8e-4, Ions matched by search engine: 0/0

Fragment match tolerance used for search: 0.06 Da

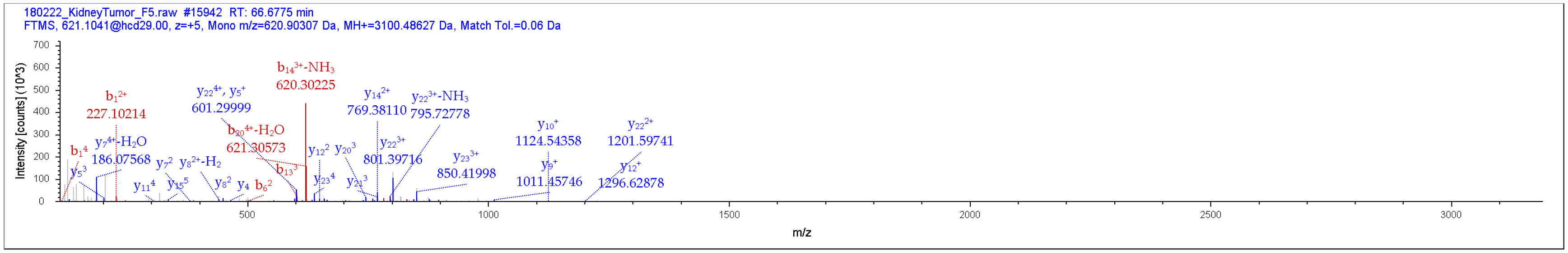

Sequence: SVAAAAGPAPQSLDLHK, K17-Guanidinyl (42.02180 Da), S1-Hex(1)HexNAc(1) (365.13220 Da)

Charge: +3, Monoisotopic m/z: 680.67915 Da (-0.5 mmu/-0.74 ppm), MH+: 2040.02289 Da, RT: 55.5730 min,

Identified with: Sequest HT (v1.17); XCorr:5.21, Percolator q-Value:0.0e0, Percolator PEP:7.6e-6, Ions matched by search engine: 0/0

Fragment match tolerance used for search: 0.06 Da

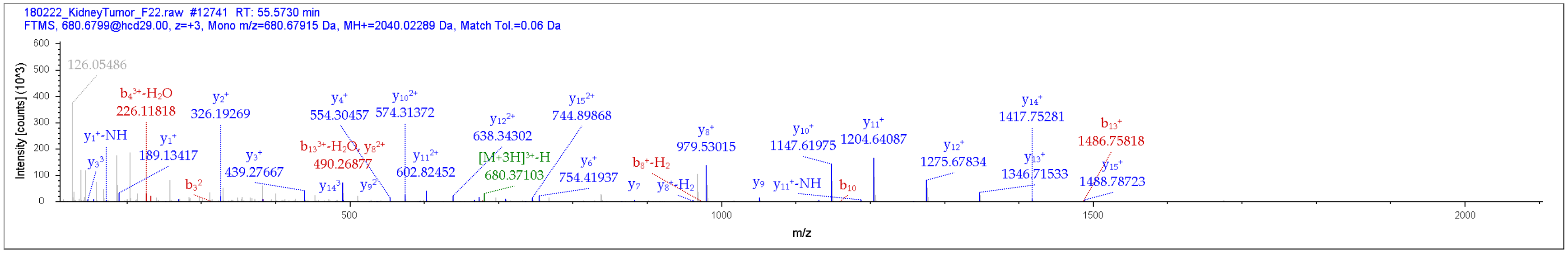

Sequence: STVAPPIHQGPAVP, S1-Hex(1)HexNAc(1) (365.13220 Da), T2-Hex(1)HexNAc(1) (365.13220 Da)

Charge: +2, Monoisotopic m/z: 1051.00854 Da (+1.37 mmu/+1.3 ppm), MH+: 2101.00980 Da, RT: 55.2389 min,

Identified with: Sequest HT (v1.17); XCorr:1.06, Percolator q-Value:6.5e-3, Percolator PEP:1.3e-1, Ions matched by search engine: 0/0

Fragment match tolerance used for search: 0.06 Da

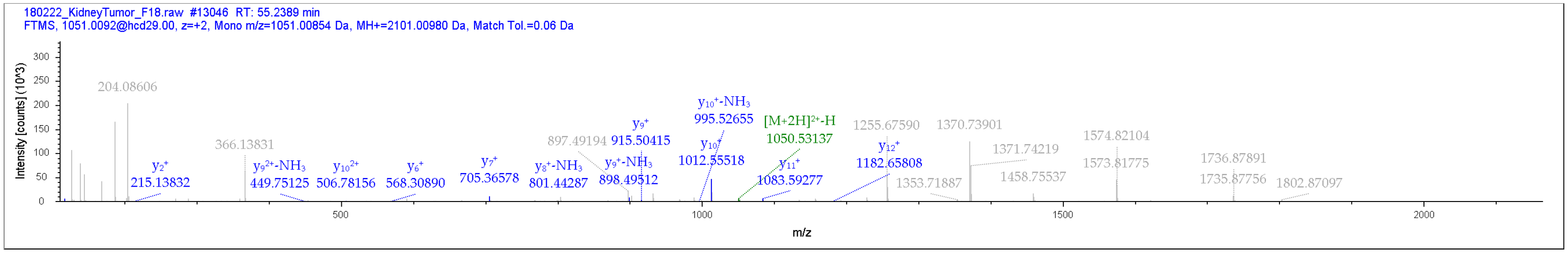

Sequence: STQSPDLEPILR, T2-HexNAc (203.07937 Da), S1-Hex(1)HexNAc(1) (365.13220 Da)

Charge: +3, Monoisotopic m/z: 641.98086 Da (-0.02 mmu/-0.03 ppm), MH+: 1923.92803 Da, RT: 67.1564 min,

Identified with: Sequest HT (v1.17); XCorr:2.42, Percolator q-Value:2.2e-3, Percolator PEP:9.1e-2, Ions matched by search engine: 0/0

Fragment match tolerance used for search: 0.06 Da

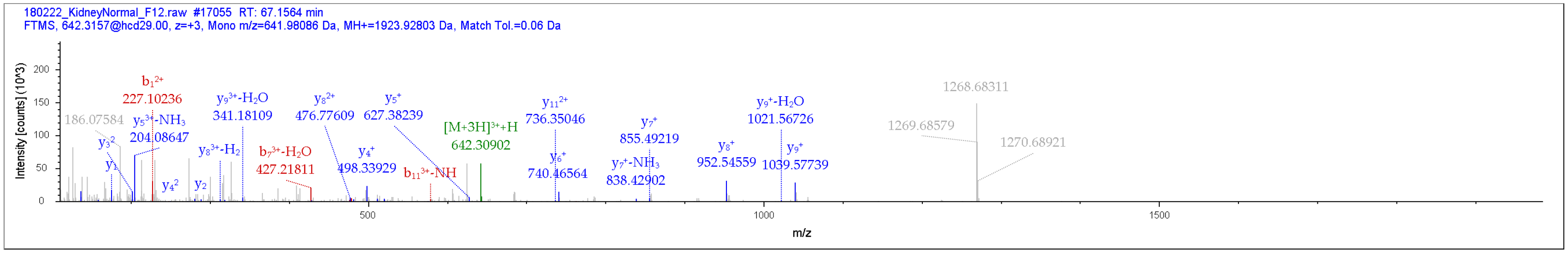

Sequence: STPTVQVTPQLETK, S1-HexNAc (203.07937 Da), K14-Guanidinyl (42.02180 Da), T2-Hex(1)HexNAc(1) (365.13220 Da)

Charge: +3, Monoisotopic m/z: 713.69066 Da (+0.78 mmu/+1.09 ppm), MH+: 2139.05742 Da, RT: 59.1132 min,

Identified with: Sequest HT (v1.17); XCorr:3.38, Percolator q-Value:0.0e0, Percolator PEP:4.3e-3, Ions matched by search engine: 0/0

Fragment match tolerance used for search: 0.06 Da

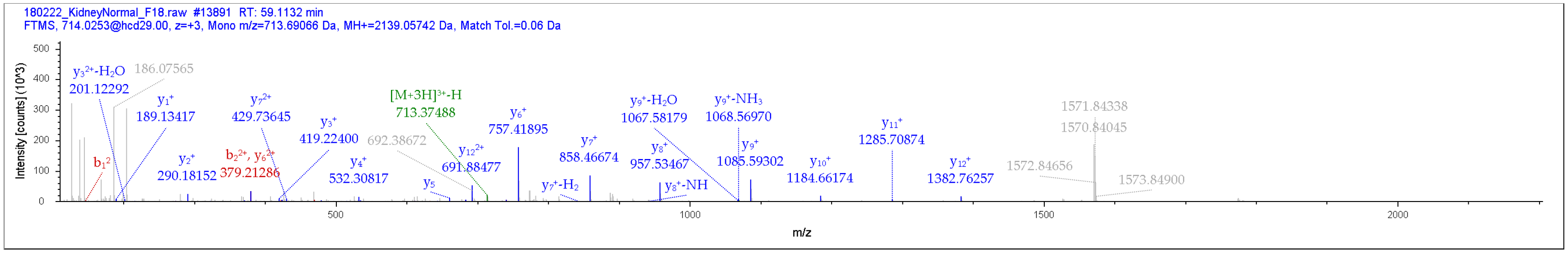

Sequence: STPPTPSPSCCHPR, C10-Carbamidomethyl (57.02146 Da), C11-Carbamidomethyl (57.02146 Da), T2-HexNAc (203.07937 Da)

Charge: +3, Monoisotopic m/z: 595.26220 Da (-0.59 mmu/-0.99 ppm), MH+: 1783.77203 Da, RT: 23.9140 min,

Identified with: Sequest HT (v1.17); XCorr:3.50, Percolator q-Value:0.0e0, Percolator PEP:5.2e-4, Ions matched by search engine: 0/0

Fragment match tolerance used for search: 0.06 Da

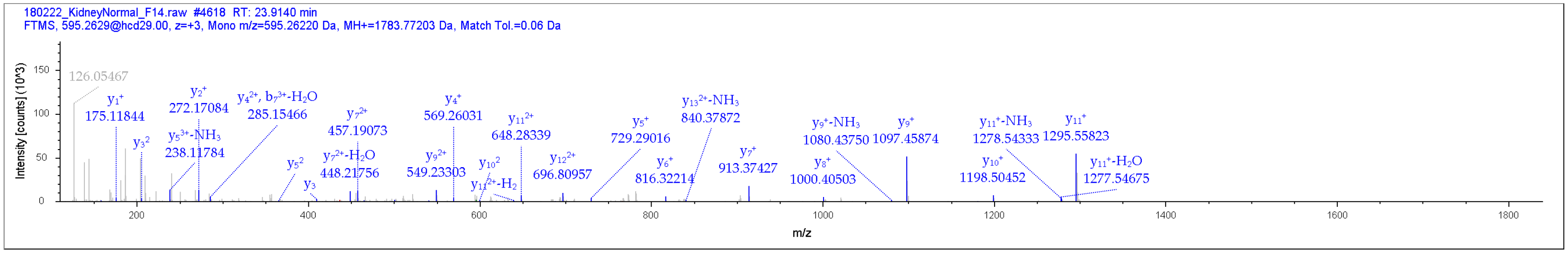

Sequence: STPPTPSPSCCHPR, C10-Carbamidomethyl (57.02146 Da), C11-Carbamidomethyl (57.02146 Da), T2-Hex(1)HexNAc(1) (365.13220 Da)

Charge: +3, Monoisotopic m/z: 649.28271 Da (+2.33 mmu/+3.58 ppm), MH+: 1945.83359 Da, RT: 29.7574 min,

Identified with: Sequest HT (v1.17); XCorr:2.86, Percolator q-Value:0.0e0, Percolator PEP:3.4e-4, Ions matched by search engine: 0/0

Fragment match tolerance used for search: 0.06 Da

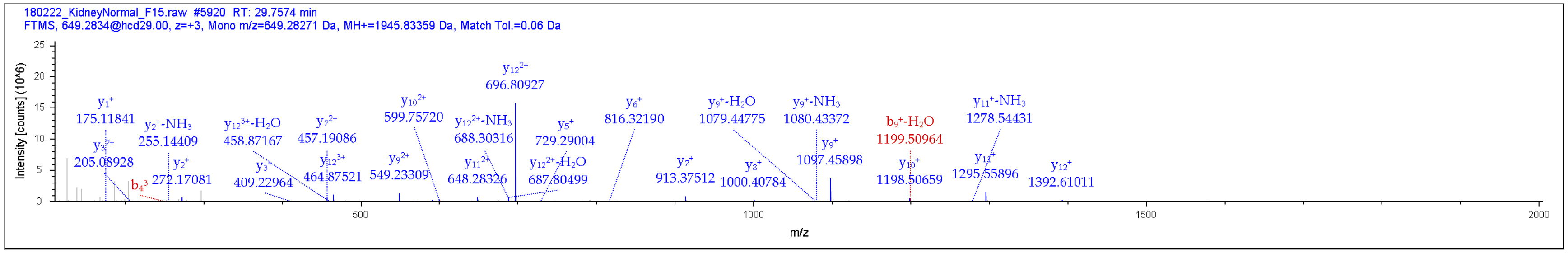

Sequence: STPPTPSPSCCHPR, C10-Carbamidomethyl (57.02146 Da), C11-Carbamidomethyl (57.02146 Da), T2-Hex(1)HexNAc(1) (365.13220 Da)

Charge: +3, Monoisotopic m/z: 649.28014 Da (-0.25 mmu/-0.39 ppm), MH+: 1945.82585 Da, RT: 30.5794 min,

Identified with: Sequest HT (v1.17); XCorr:2.97, Percolator q-Value:0.0e0, Percolator PEP:7.6e-6, Ions matched by search engine: 0/0

Fragment match tolerance used for search: 0.06 Da

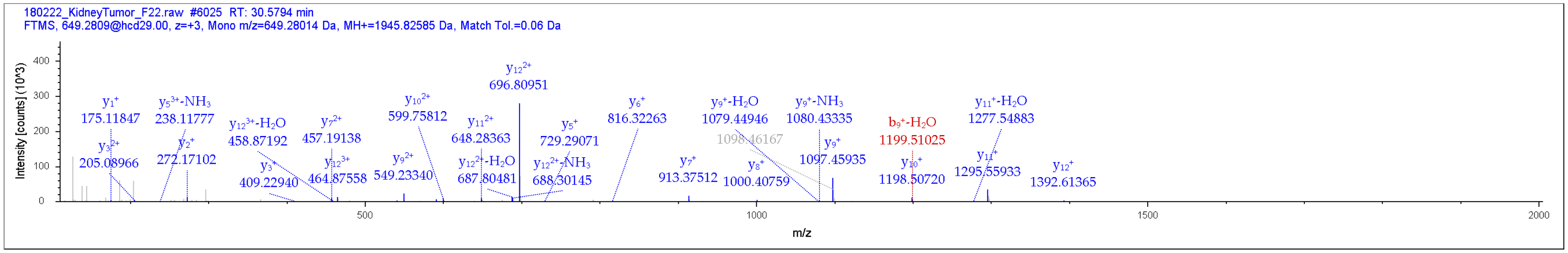

Sequence: STPPTPSPSCCHPR, C10-Carbamidomethyl (57.02146 Da), C11-Carbamidomethyl (57.02146 Da), S1-Hex(1)HexNAc(1) (365.13220 Da)

Charge: +3, Monoisotopic m/z: 649.28142 Da (+1.03 mmu/+1.59 ppm), MH+: 1945.82971 Da, RT: 28.4168 min,

Identified with: Sequest HT (v1.17); XCorr:3.92, Percolator q-Value:0.0e0, Percolator PEP:8.8e-5, Ions matched by search engine: 0/0

Fragment match tolerance used for search: 0.06 Da

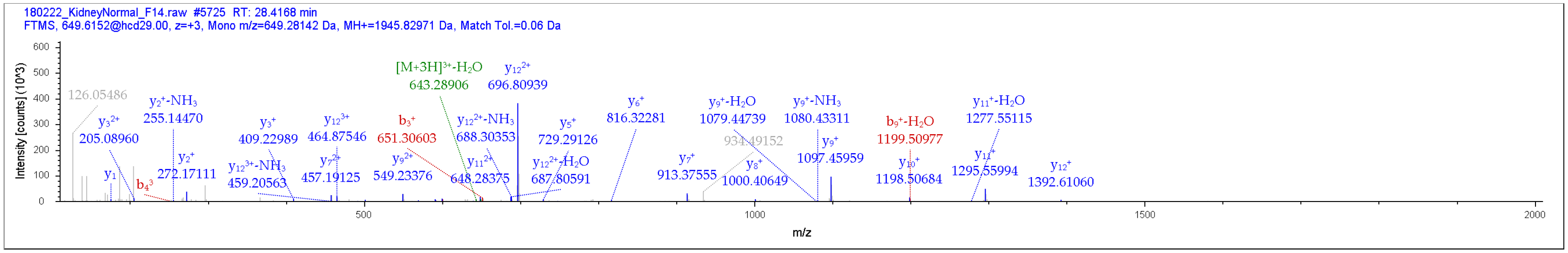

Sequence: STPPTPSPSCCHPR, C10-Carbamidomethyl (57.02146 Da), C11-Carbamidomethyl (57.02146 Da), S1-HexNAc (203.07937 Da)

Charge: +4, Monoisotopic m/z: 446.69771 Da (-1.2 mmu/-2.68 ppm), MH+: 1783.76902 Da, RT: 25.1498 min,

Identified with: Sequest HT (v1.17); XCorr:4.87, Percolator q-Value:0.0e0, Percolator PEP:1.5e-3, Ions matched by search engine: 0/0

Fragment match tolerance used for search: 0.06 Da

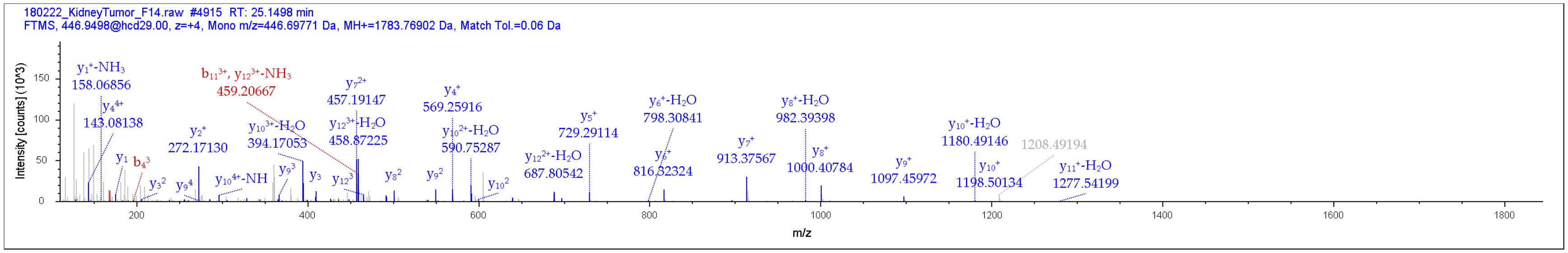

Sequence: STPPTPSPSCCHPR, C10-Carbamidomethyl (57.02146 Da), C11-Carbamidomethyl (57.02146 Da), S1-HexNAc (203.07937 Da)

Charge: +3, Monoisotopic m/z: 595.26337 Da (+0.59 mmu/+0.99 ppm), MH+: 1783.77556 Da, RT: 29.3748 min,

Identified with: Sequest HT (v1.17); XCorr:3.54, Percolator q-Value:0.0e0, Percolator PEP:4.1e-4, Ions matched by search engine: 0/0

Fragment match tolerance used for search: 0.06 Da

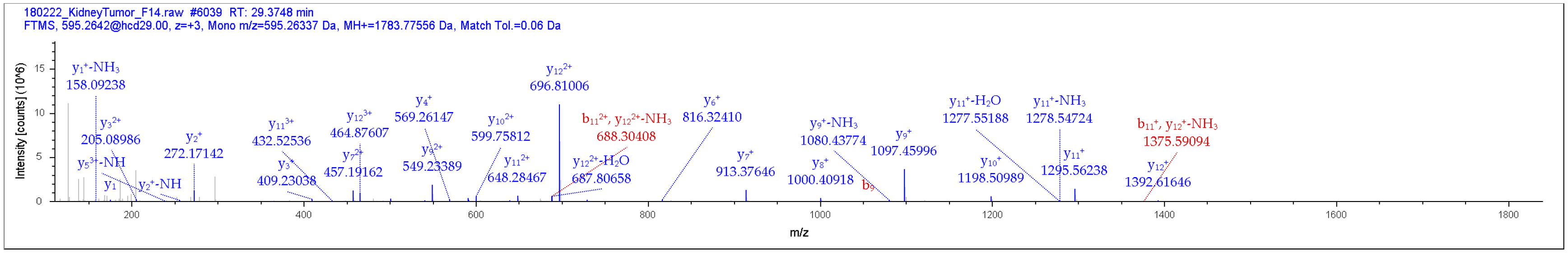

Sequence: SPSTPPTPSPSCCHPR, C12-Carbamidomethyl (57.02146 Da), C13-Carbamidomethyl (57.02146 Da), S1-Hex(1)HexNAc(1) (365.13220 Da)

Charge: +3, Monoisotopic m/z: 710.64181 Da (-0.18 mmu/-0.25 ppm), MH+: 2129.91088 Da, RT: 34.5310 min,

Identified with: Sequest HT (v1.17); XCorr:4.73, Percolator q-Value:0.0e0, Percolator PEP:1.8e-7, Ions matched by search engine: 0/0

Fragment match tolerance used for search: 0.06 Da

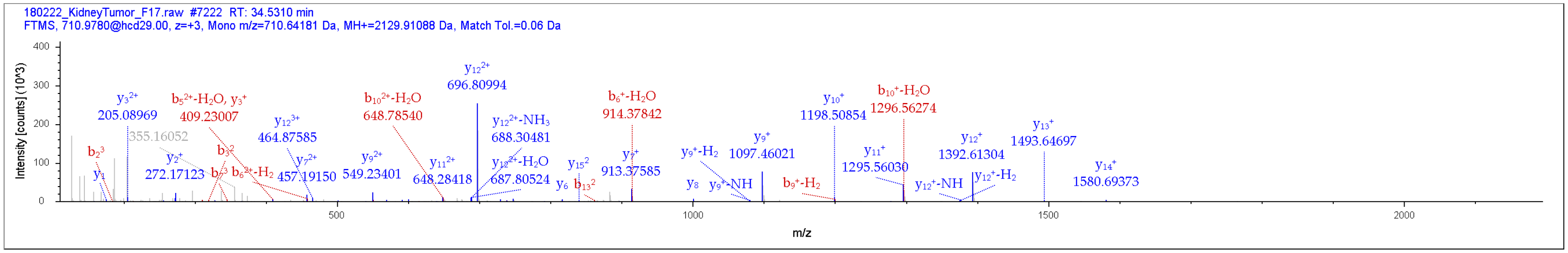

Sequence: SPSTPPTPSPSCCHPR, C12-Carbamidomethyl (57.02146 Da), C13-Carbamidomethyl (57.02146 Da), T4-HexNAc (203.07937 Da), S1-Hex(1)HexNAc(1) (365.13220 Da), S3-Hex(1)HexNAc(1) (365.13220 Da)

Charge: +3, Monoisotopic m/z: 900.04593 Da (+0.08 mmu/+0.09 ppm), MH+: 2698.12323 Da, RT: 32.6138 min,

Identified with: Sequest HT (v1.17); XCorr:2.64, Percolator q-Value:0.0e0, Percolator PEP:1.1e-3, Ions matched by search engine: 0/0

Fragment match tolerance used for search: 0.06 Da

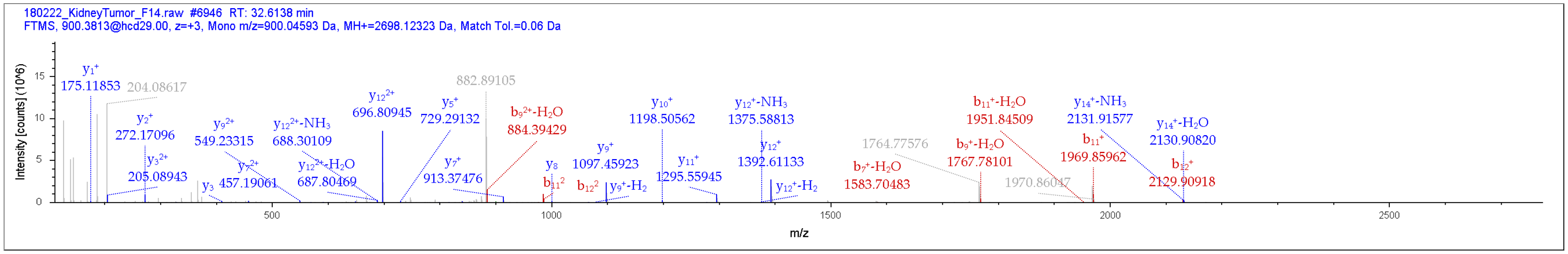

Sequence: SPSTPPTPSPSCCHPR, C12-Carbamidomethyl (57.02146 Da), C13-Carbamidomethyl (57.02146 Da), T4-HexNAc (203.07937 Da), S1-Hex(1)HexNAc(1) (365.13220 Da), S3-Hex(1)HexNAc(1) (365.13220 Da)

Charge: +4, Monoisotopic m/z: 675.28788 Da (+1.68 mmu/+2.49 ppm), MH+: 2698.12971 Da, RT: 31.7167 min,

Identified with: Sequest HT (v1.17); XCorr:3.09, Percolator q-Value:0.0e0, Percolator PEP:2.3e-4, Ions matched by search engine: 0/0

Fragment match tolerance used for search: 0.06 Da

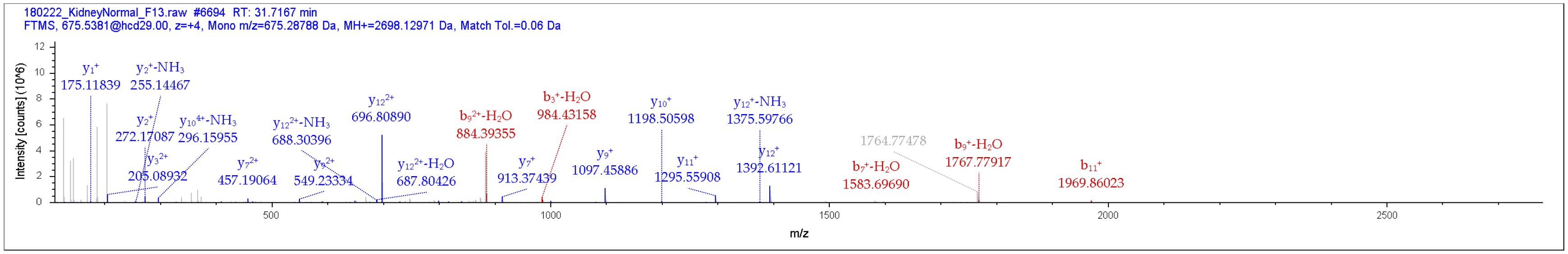

Sequence: SGVLPGGGFVASAAAVAGPEMQTGR, S1-Hex(1)HexNAc(1) (365.13220 Da)

Charge: +3, Monoisotopic m/z: 884.76761 Da (+2.05 mmu/+2.31 ppm), MH+: 2652.28827 Da, RT: 95.7091 min,

Identified with: Sequest HT (v1.17); XCorr:6.59, Percolator q-Value:0.0e0, Percolator PEP:5.8e-8, Ions matched by search engine: 0/0

Fragment match tolerance used for search: 0.06 Da

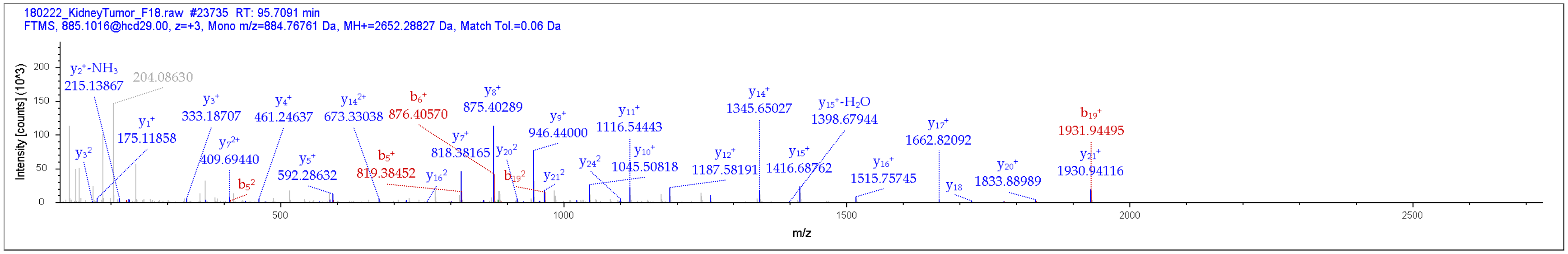

Sequence: SGVLPGGGFVASAAAVAGPEMQTGR, M21-Oxidation (15.99492 Da), S1-Hex(1)HexNAc(1) (365.13220 Da)

Charge: +3, Monoisotopic m/z: 890.09841 Da (+1.21 mmu/+1.36 ppm), MH+: 2668.28067 Da, RT: 87.3296 min,

Identified with: Sequest HT (v1.17); XCorr:5.68, Percolator q-Value:0.0e0, Percolator PEP:2.0e-7, Ions matched by search engine: 0/0

Fragment match tolerance used for search: 0.06 Da

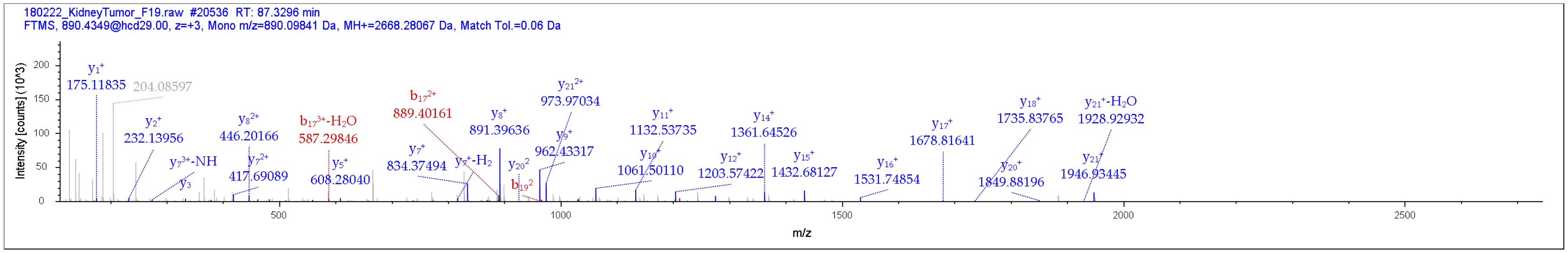

Sequence: SGSPDLSETTSAFHEANLER, S1-Hex(1)HexNAc(1) (365.13220 Da)

Charge: +3, Monoisotopic m/z: 838.37755 Da (+0.64 mmu/+0.77 ppm), MH+: 2513.11810 Da, RT: 68.6861 min,

Identified with: Sequest HT (v1.17); XCorr:5.28, Percolator q-Value:0.0e0, Percolator PEP:5.6e-7, Ions matched by search engine: 0/0

Fragment match tolerance used for search: 0.06 Da

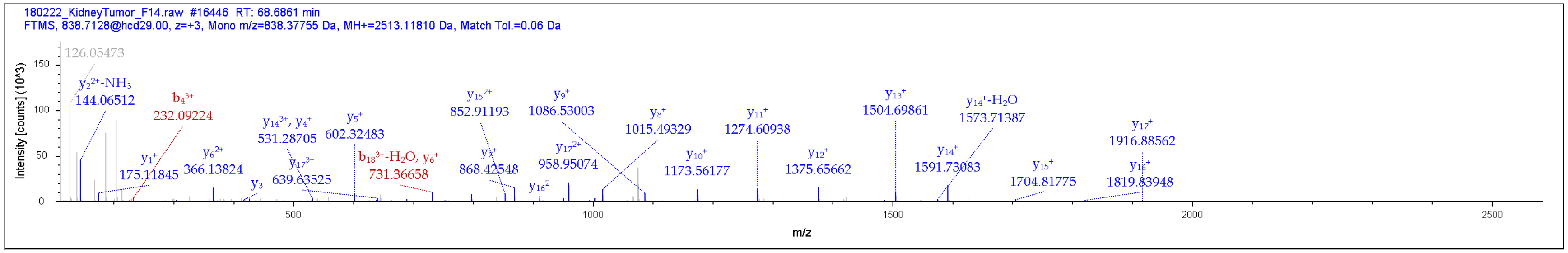

Sequence: SGSGYVPGSVSAAFVTCPNEK, C17-Carbamidomethyl (57.02146 Da), S3-HexNAc (203.07937 Da), K21-Guanidinyl (42.02180 Da), S1-Hex(1)HexNAc(1) (365.13220 Da)

Charge: +3, Monoisotopic m/z: 908.73940 Da (-5.2 mmu/-5.73 ppm), MH+: 2724.20366 Da, RT: 81.6999 min,

Identified with: Sequest HT (v1.17); XCorr:3.70, Percolator q-Value:5.0e-5, Percolator PEP:2.6e-3, Ions matched by search engine: 0/0

Fragment match tolerance used for search: 0.06 Da

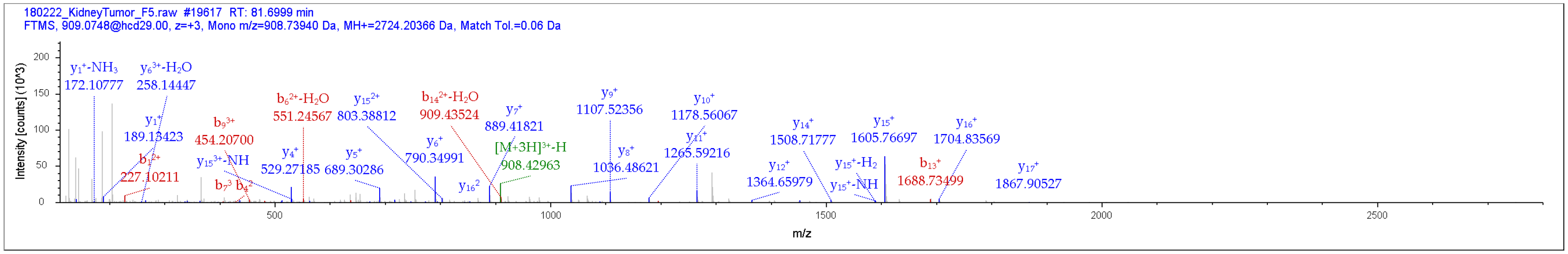

Sequence: SGQQPSVGQQMIFEEHGFR, S1-Hex(1)HexNAc(1) (365.13220 Da)

Charge: +3, Monoisotopic m/z: 843.05193 Da (+0.23 mmu/+0.27 ppm), MH+: 2527.14124 Da, RT: 75.6487 min,

Identified with: Sequest HT (v1.17); XCorr:4.63, Percolator q-Value:0.0e0, Percolator PEP:1.5e-5, Ions matched by search engine: 0/0

Fragment match tolerance used for search: 0.06 Da

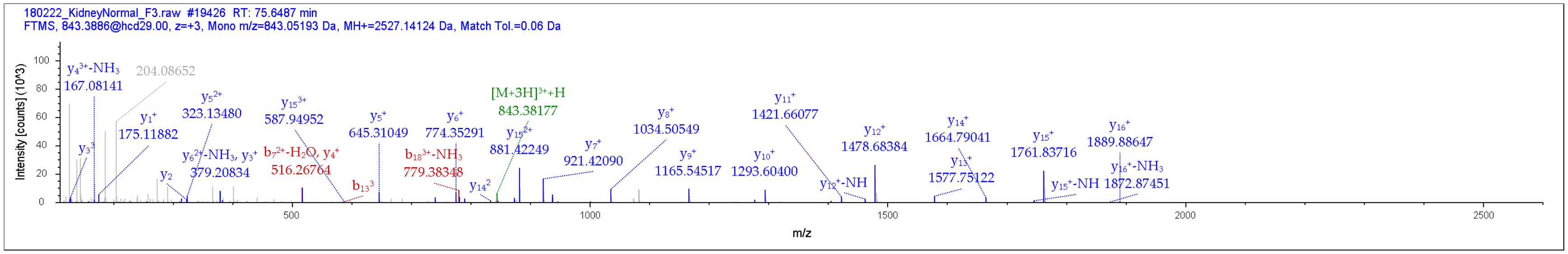

Sequence: SGPPAQDQDICPASEDESGQWK, C11-Carbamidomethyl (57.02146 Da), K22-Guanidinyl (42.02180 Da), S14-Hex(1)HexNAc(1) (365.13220 Da)

Charge: +4,   Monoisotopic m/z: 703.04953 Da (+0.55 mmu/+0.78 ppm),   MH+: 2809.17630 Da,   RT: 62.1231 min,

Identified with: Sequest HT (v1.17); XCorr:4.45, Percolator q-Value:2.1e-4, Percolator PEP:1.4e-2, Ions matched by search engine: 0/0

Fragment match tolerance used for search: 0.06 Da

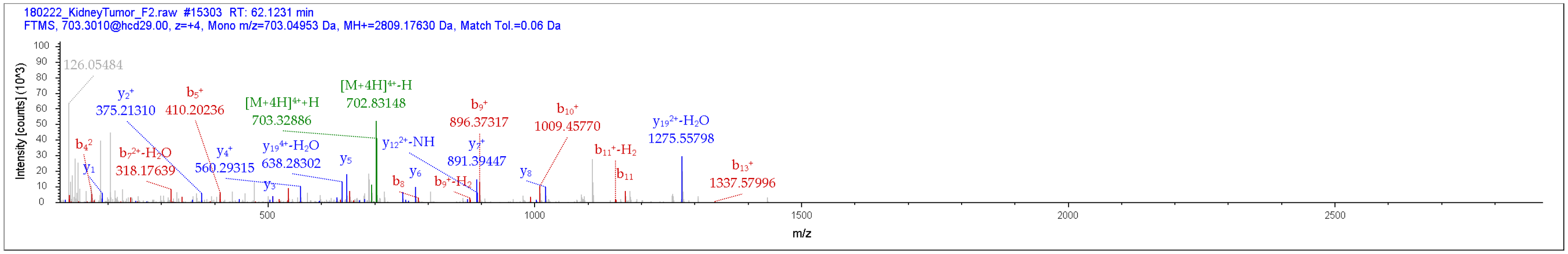

Sequence: SGPPAQDQDICPASEDESGQWK, C11-Carbamidomethyl (57.02146 Da), K22-Guanidinyl (42.02180 Da), S1-Hex(1)HexNAc(1) (365.13220 Da)

Charge: +3,   Monoisotopic m/z: 937.06352 Da (+0.64 mmu/+0.68 ppm),   MH+: 2809.17601 Da,   RT: 62.6727 min,

Identified with: Sequest HT (v1.17); XCorr:6.32, Percolator q-Value:0.0e0, Percolator PEP:3.7e-7, Ions matched by search engine: 0/0

Fragment match tolerance used for search: 0.06 Da

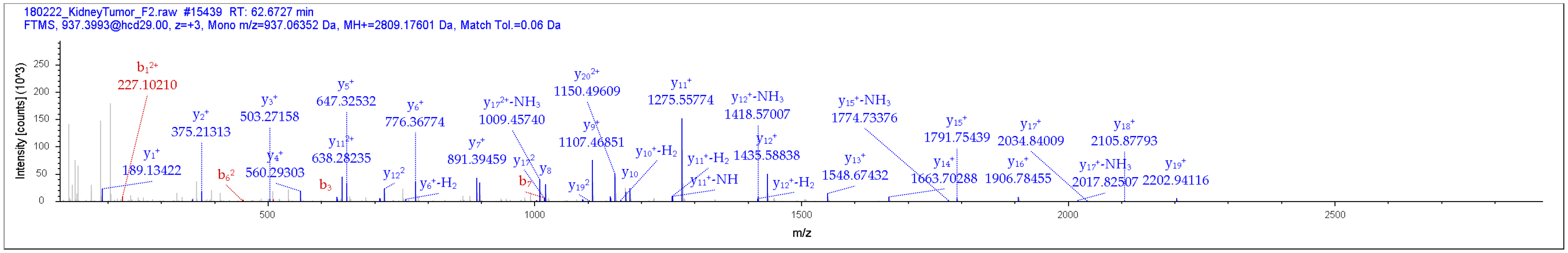

Sequence: SGPPAQDQDICPASEDESGQWK, C11-Carbamidomethyl (57.02146 Da), K22-Guanidinyl (42.02180 Da), S1-Hex(1)HexNAc(1) (365.13220 Da)

Charge: +3, Monoisotopic m/z: 937.06421 Da (+1.32 mmu/+1.41 ppm), MH+: 2809.17806 Da, RT: 61.7246 min,

Identified with: Sequest HT (v1.17); XCorr:6.18, Percolator q-Value:0.0e0, Percolator PEP:4.8e-6, Ions matched by search engine: 0/0

Fragment match tolerance used for search: 0.06 Da

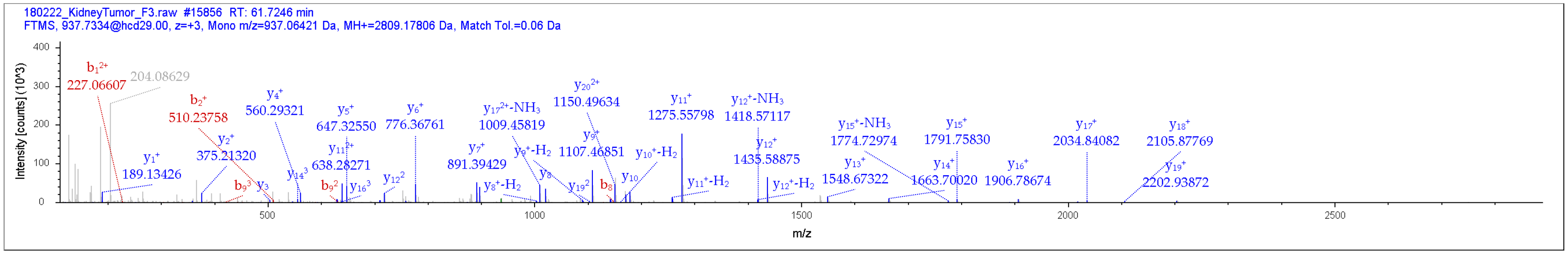

Sequence: SGLPSGIAEVSGESSR, S1-HexNAc (203.07937 Da)

Charge: +3, Monoisotopic m/z: 579.28217 Da (-0.83 mmu/-1.44 ppm), MH+: 1735.83197 Da, RT: 61.5373 min,

Identified with: Sequest HT (v1.17); XCorr:4.25, Percolator q-Value:0.0e0, Percolator PEP:9.8e-4, Ions matched by search engine: 0/0

Fragment match tolerance used for search: 0.06 Da

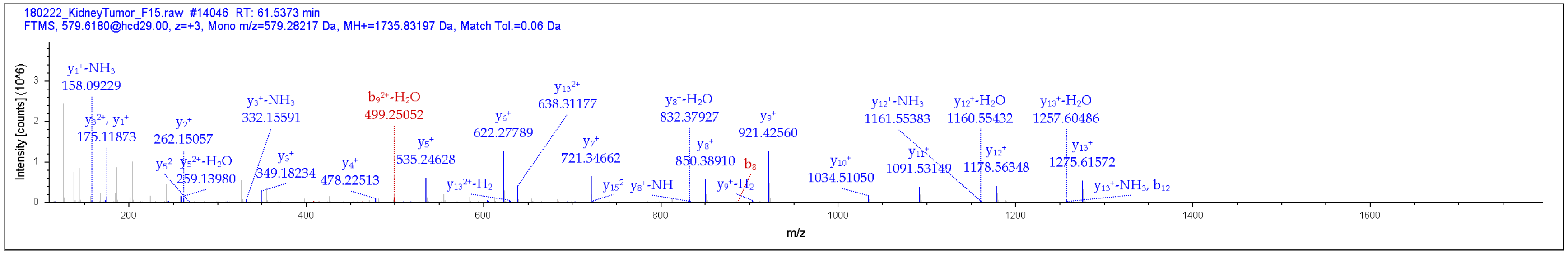

Sequence: SGLPSGFSGEYSGVDLGSGPP, S18-Hex(1)HexNAc(1) (365.13220 Da)

Charge: +2, Monoisotopic m/z: 1166.52259 Da (+1.42 mmu/+1.22 ppm), MH+: 2332.03791 Da, RT: 92.4162 min,

Identified with: Sequest HT (v1.17); XCorr:3.31, Percolator q-Value:8.0e-4, Percolator PEP:3.3e-2, Ions matched by search engine: 0/0

Fragment match tolerance used for search: 0.06 Da

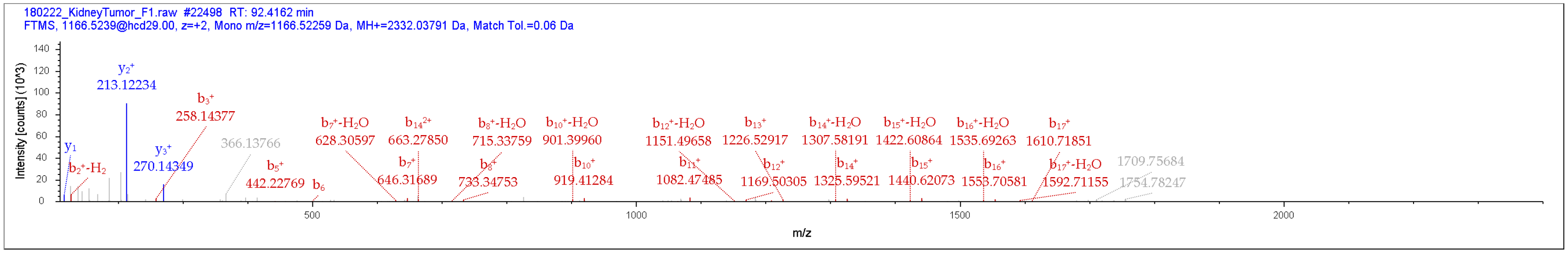

Sequence: SGAPDMSGEHSGFLDLSGLQSGLIEP, M6-Oxidation (15.99492 Da), S21-Hex(1)HexNAc(1) (365.13220 Da)

Charge: +3, Monoisotopic m/z: 994.78533 Da (+0.2 mmu/+0.2 ppm), MH+: 2982.34144 Da, RT: 109.8414 min,

Identified with: Sequest HT (v1.17); XCorr:6.19, Percolator q-Value:5.0e-5, Percolator PEP:1.9e-3, Ions matched by search engine: 0/0

Fragment match tolerance used for search: 0.06 Da

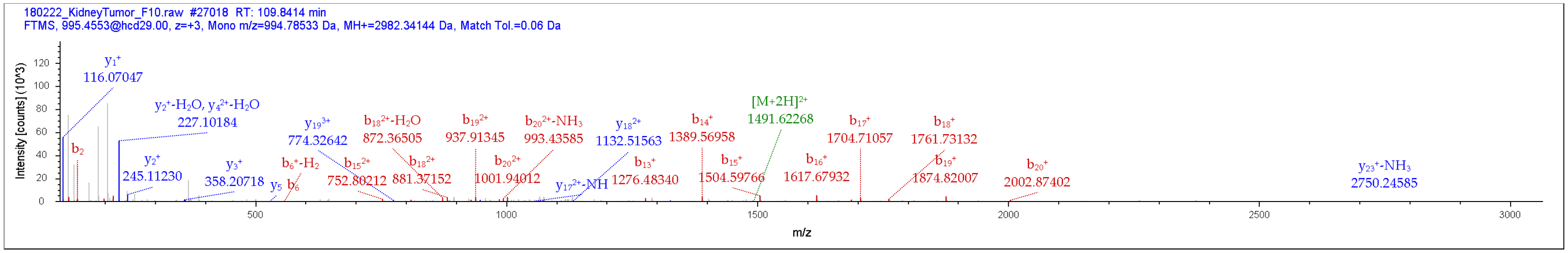

Sequence: SFQPEFSSGAEEALVDH, S1-Hex(1)HexNAc(1) (365.13220 Da)

Charge: +2, Monoisotopic m/z: 1107.98285 Da (+1.17 mmu/+1.05 ppm), MH+: 2214.95843 Da, RT: 82.1249 min,

Identified with: Sequest HT (v1.17); XCorr:2.94, Percolator q-Value:0.0e0, Percolator PEP:2.3e-4, Ions matched by search engine: 0/0

Fragment match tolerance used for search: 0.06 Da

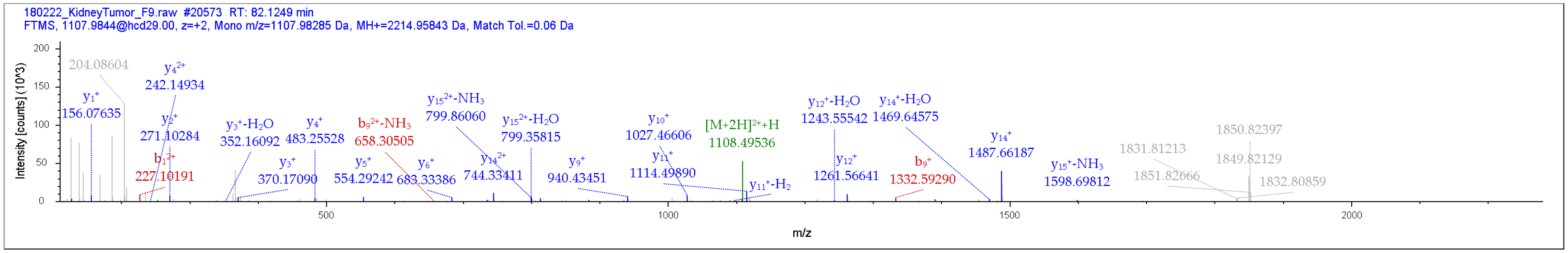

Sequence: SESNNYMNHVAK, M7-Oxidation (15.99492 Da), K12-Guanidinyl (42.02180 Da), S1-Hex(1)HexNAc(1) (365.13220 Da)

Charge: +3, Monoisotopic m/z: 606.26025 Da (+0.27 mmu/+0.44 ppm), MH+: 1816.76620 Da, RT: 24.5959 min,

Identified with: Sequest HT (v1.17); XCorr:1.95, Percolator q-Value:0.0e0, Percolator PEP:3.4e-3, Ions matched by search engine: 0/0

Fragment match tolerance used for search: 0.06 Da

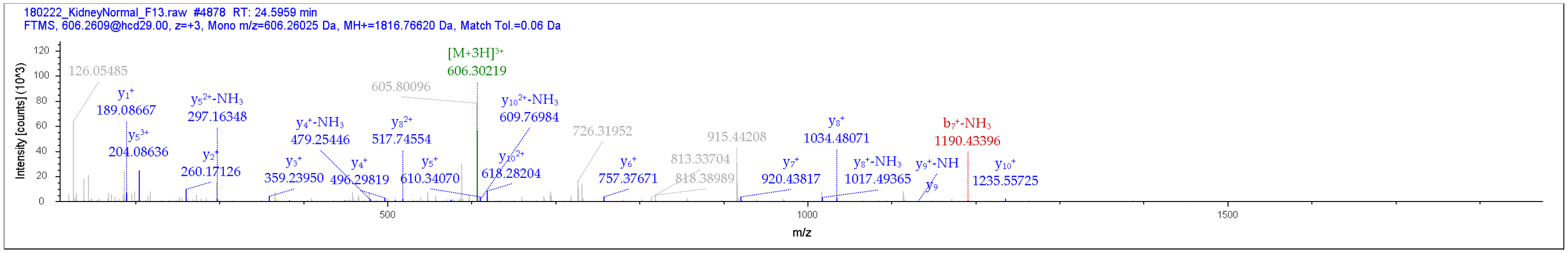

Sequence: SCLDTQYTCDNHQCISK, C2-Carbamidomethyl (57.02146 Da), C9-Carbamidomethyl (57.02146 Da), C14-Carbamidomethyl (57.02146 Da), K17-Guanidinyl (42.02180 Da), S1-Hex(1)HexNAc(1) (365.13220 Da)

Charge: +3, Monoisotopic m/z: 846.34478 Da (-0.9 mmu/-1.07 ppm), MH+: 2537.01979 Da, RT: 37.0392 min,

Identified with: Sequest HT (v1.17); XCorr:3.22, Percolator q-Value:0.0e0, Percolator PEP:1.3e-4, Ions matched by search engine: 0/0

Fragment match tolerance used for search: 0.06 Da

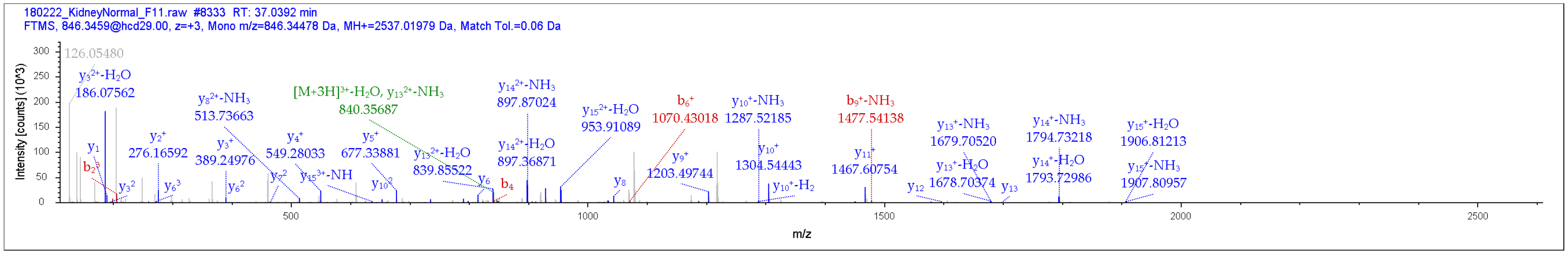

Sequence: SEMDVPPAHPEEEIVLR, M3-Oxidation (15.99492 Da), S1-Hex(1)HexNAc(1) (365.13220 Da)

Charge: +3, Monoisotopic m/z: 777.02925 Da (-0.66 mmu/-0.84 ppm), MH+: 2329.07320 Da, RT: 64.7201 min,

Identified with: Sequest HT (v1.17); XCorr:3.36, Percolator q-Value:0.0e0, Percolator PEP:2.2e-5, Ions matched by search engine: 0/0

Fragment match tolerance used for search: 0.06 Da

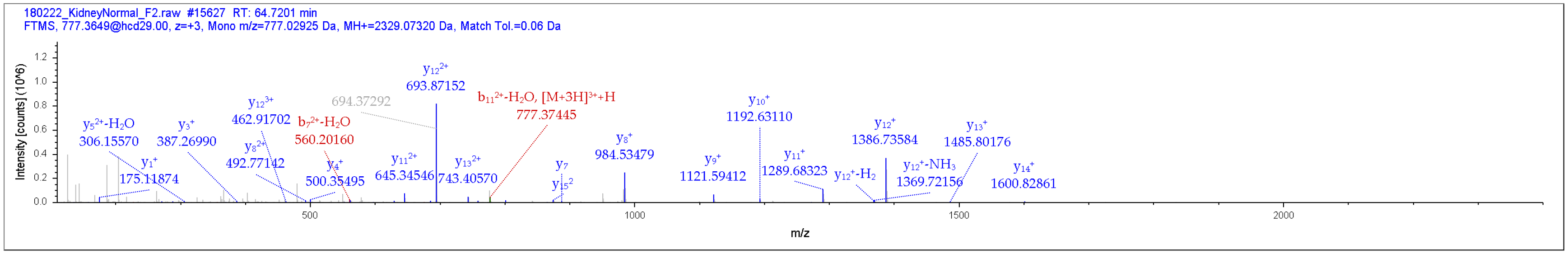

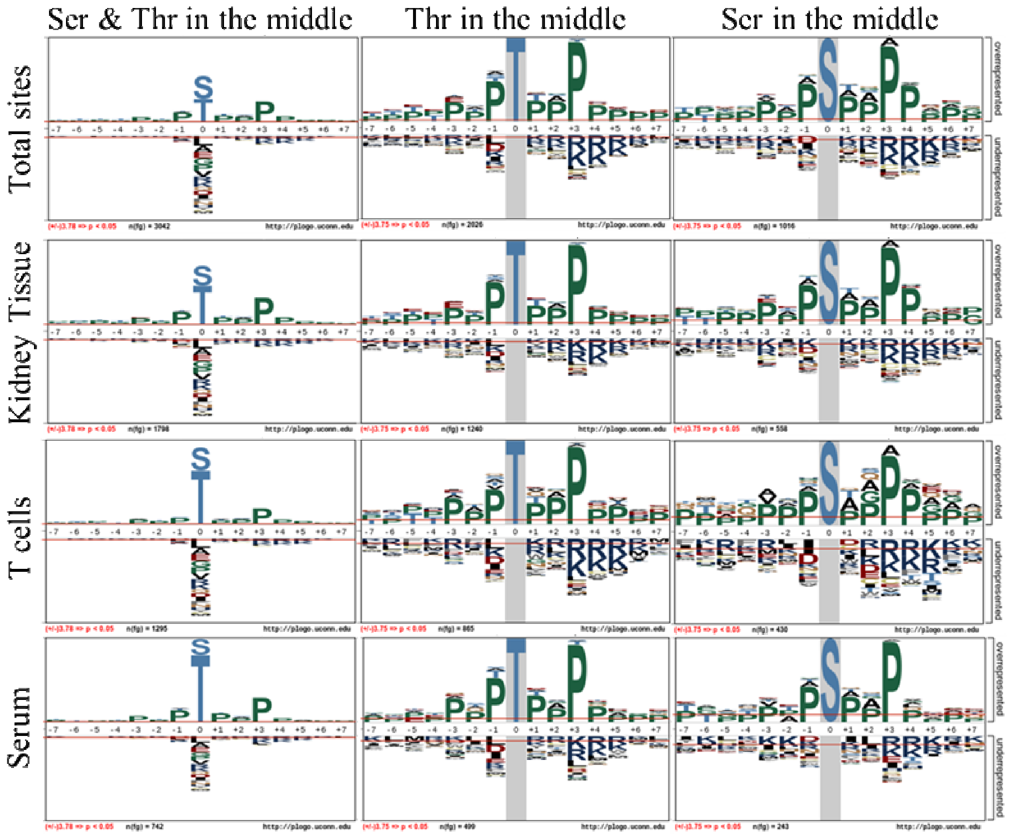

Appendix Figure S3: Motif analysis of the O-linked glycosylation sites identified from different samples.

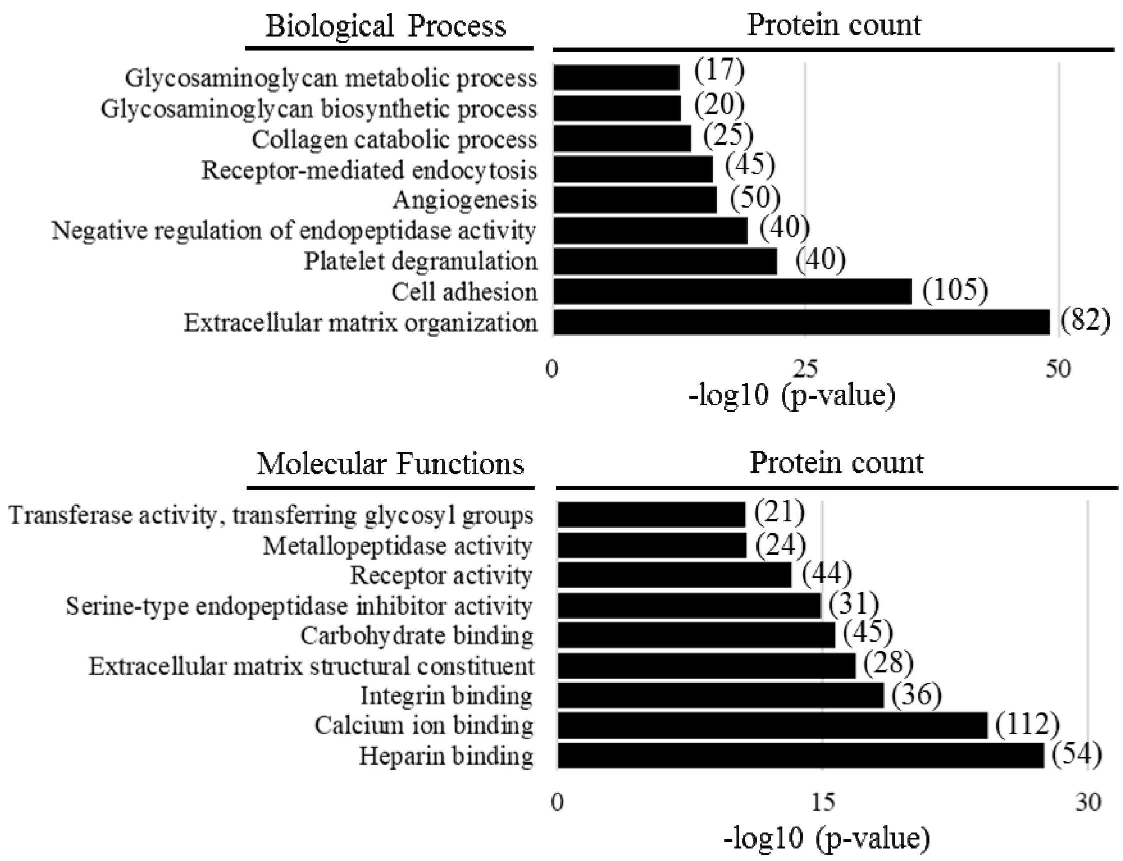

Appendix Figure S4: Biological processes and molecular functions of the identified O-linked glycoproteins.

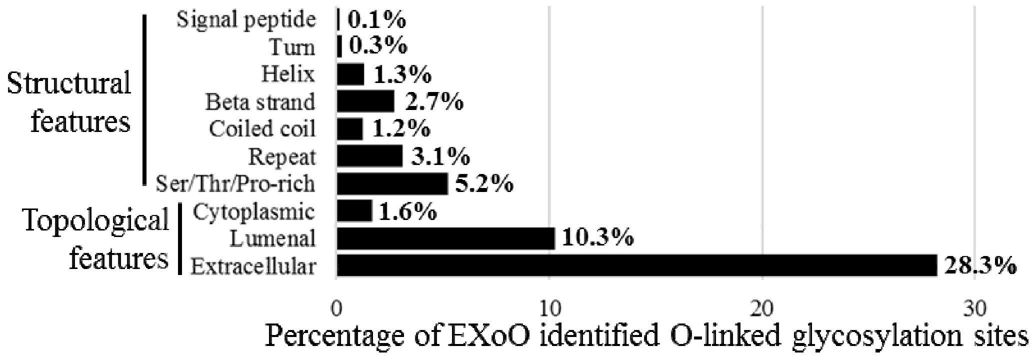

Appendix Figure S5: Localization of O-linked glycosylation sites to protein structural and topological features.

Protein Accession #	Gene Name	Protein Name	Fold Change tumor/normal (log2)	Differential PSM between tumor and normal
P05121	SERPINE1	Plasminogen activator inhibitor 1	Tumor	18
Q16790	CA9	Carbonic anhydrase 9	3.7	12
Q8NHJ6	LILRB4	Leukocyte immunoglobulin-like receptor subfamily B member 4	3.0	14
P27658	COL8A1	Collagen alpha-1(VIII) chain	2.7	127
P48960	CD97	EGF-like module-containing mucin-like hormone receptor-like 2	2.5	14
P19823	ITIH2	Inter-alpha-trypsin inhibitor heavy chain H2	2.5	415
P50281	MMP14	Matrix metalloproteinase-14	2.5	18
Q68BL8	OLFML2B	Olfactomedin-like protein 2B isoform 2	2.3	15
Q99715	COL12A1	Collagen alpha-1(XII) chain	2.3	412
P15502	ELN	Elastin	2.2	13
P49257	LMAN1	Protein ERGIC-53	2.2	14
P98095	FBLN2	Fibulin-2	2.1	247
Q14767	LTBP2	Latent-transforming growth factor beta-binding protein 2	2.0	198
Q96DB9	FXYD5	FXYD domain-containing ion transport regulator 5	1.9	17
Q9BY76	ANGPTL4	Angiopoietin-related protein 4	1.8	96
Q9UKP4	ADAMTS7	A disintegrin and metalloproteinase with thrombospondin motifs 7	1.7	88
P06213	INSR	Insulin receptor	1.6	13
Q12805	EFEMP1	EGF-containing fibulin-like extracellular matrix protein 1	1.6	258
Q92896	GLG1	Golgi apparatus protein 1	1.5	12
Q9BXX0	EMILIN2	EMILIN-2	1.5	58
P16112	ACAN	Aggrecan core protein	1.5	832
Q9UBP4	DKK3	Dickkopf-related protein 3	1.4	32
Q9NS15	LTBP3	Latent-transforming growth factor beta-binding protein 3	1.4	12
Q12797	ASPH	Aspartyl/asparaginyl beta-hydroxylase	1.3	42
P23470	PTPRG	Receptor-type tyrosine-protein phosphatase gamma	1.3	12
Q9H8L6	MMRN2	Multimerin-2	1.3	14
P13611	VCAN	Versican core protein	1.3	1216
Q8IUX7	AEBP1	Adipocyte enhancer-binding protein 1	1.2	42
Q8TDQ1	CD300LF	CMRF35-like molecule 1	1.2	11
Q14515	SPARCL1	SPARC-like protein 1	1.1	40
Q14766	LTBP1	Latent-transforming growth factor beta-binding protein 1	1.1	13
Q14314	FGL2	Fibroleukin	1.1	27
Q6UXD5	SEZ6L2	Seizure 6-like protein 2	1.1	19
P02751	FN1	Fibronectin	1.1	218
P16070	CD44	CD44 antigen	1.0	17
Q6IEE7	TMEM132E	Transmembrane protein 132E	-1.0	14
P15144	ANPEP	Aminopeptidase N	-1.1	181
O60494	CUBN	Cubilin	-1.2	66
Q8N474	SFRP1	Secreted frizzled-related protein 1	-1.2	47
O75309	CDH16	Cadherin-16	-1.2	14
Q9NR34	MAN1C1	Mannosyl-oligosaccharide 1,2-alpha-mannosidase IC	-1.2	11
P98164	LRP2	Low-density lipoprotein receptor-related protein 2	-1.2	818
O43451	MGAM	Maltase-glucoamylase	-1.3	62
P02724	GYPA	Glycophorin-A	-1.3	40
Q9ULC0	EMCN	Endomucin	-1.4	142
O95428	PAPLN	Papilin	-1.4	79
Q9UGT4	SUSD2	Sushi domain-containing protein 2	-1.6	14
Q9UJW2	TINAG	Tubulointerstitial nephritis antigen	-1.6	60
Q6ZMM2	ADAMTSL5	ADAMTS-like protein 5	-1.7	13
P36269	GGT5	Gamma-glutamyltransferase 5	-1.7	13
Q5IJ48	CRB2	Protein crumbs homolog 2	-1.9	22
Q96SM3	CPXM1	Probable carboxypeptidase X1	-2.0	13
Q96A83	COL26A1	Collagen alpha-1(XXVI) chain	-2.1	23
Q2UY09	COL28A1	Collagen alpha-1(XXVIII) chain	Normal	15
P13866	SLC5A1	Sodium/glucose cotransporter 1	Normal	20
Q8N695	SLC5A8	Sodium-coupled monocarboxylate transporter 1	Normal	26

Appendix Table S1: Differentially expressed O-linked glycoproteins in tumor vs normal kidney tissues.
